# Bumble Bee Watch community science program increases scientific understanding of an important pollinator group across Canada and the USA

**DOI:** 10.1371/journal.pone.0303335

**Published:** 2024-05-22

**Authors:** Victoria J. MacPhail, Richard Hatfield, Sheila R. Colla

**Affiliations:** 1 Faculty of Environmental and Urban Change, York University, Toronto, Ontario, Canada; 2 The Xerces Society for Invertebrate Conservation, Portland, Oregan, United States of America; University College London, UNITED KINGDOM

## Abstract

In a time of increasing threats to bumble bees (Hymenoptera: Apidae: *Bombus*), it is important to understand their ecology and distribution. As experts are limited in resources to conduct field surveys, there is potential for community scientists to help. The Bumble Bee Watch (BBW) community science program involves volunteers taking photos of bumble bees in Canada and the USA and submitting them, along with geographic and optional plant information, to a website or through an app. Taxon experts then verify the bee species identification. The Bumble Bees of North America database (BBNA) stores data (no photographs) collected and identified by more traditional scientific methods over the same range. Here we compared BBW data to BBNA data over all years and just 2010–2020 to understand the scientific contribution of community scientists to the state of the knowledge about native bumble bees. We found that BBW had similar geographic and species coverage as BBNA. It had records from all 63 provinces, states, and territories where bumble bees occur (including four more than BBNA in 2010–2020), and represented 41 of the 48 species in BBNA (with ten more species than BBNA in 2010–2020). While BBW contributed only 8.50% of records overall, it contributed 25.06% of all records over 2010–2020. BBW confirmed the persistence of species and identified new locations of species, both inside and outside of the previously known extent of occurrences. BBW also contributed a wealth of ecological information, such as unique plant genera and species data for almost all the bee species. Thus, while BBW had fewer bee records than the BBNA database overall, it helped to fill in data gaps and provided novel information, complementing the traditional methods. This community science program is valuable in helping to inform conservation management for bumble bee species.

## Introduction

Bumble bees (Hymenoptera: Apidae: *Bombus*) are a relatively recognizable genus of bees, with their often hairy bodies, large size, and frequent visits to flowers throughout the growing season. Found across the Northern Hemisphere and in South America [1], they are important pollinators of agricultural crops and wild plants [2–5]. While we know that approximately a quarter to a third of our bumble bees in North America are in decline, knowledge gaps on stressors, ranges, population dynamics and basic natural history remain [6–10].

One impediment to wildlife conservation is the lack of knowledge about their natural history (such as habitat needs and species associations) and current status (such as current distribution and changes in their population size and distribution over time) [11–16]. Conservation scientists often lacks the resources (funding, staff time, expertise) to adequately gather this information, particularly for species with broad distributions [6,17–21]. This is particularly an issue with invertebrates, which are largely unstudied compared to vertebrates like birds or mammals [22–25].

Community science (also called citizen science or participatory-based science) [26] involves volunteers designing, collecting, analyzing and/or interpreting scientific data to answer a research question [18,27–31]. Volunteers can collect data similarly to experts (depending on skill level, training provided), often with greater coverage than researchers could cover alone and with a lower cost [18,32–36] (but see [37–40]). Participants benefit in a variety of ways including increasing their knowledge of species and field techniques and becoming more environmentally aware and engaged [15,18,29,41,42]. The data can be used independently, or combined with other programs and data sources, to increase knowledge about wildlife or habitat, inform environmental policies, aid in conservation management, and even help achieve United Nations Sustainable Development Goals [15,18,19,43–46].

The Bumble Bee Watch community science program (herein “BBW”) [47] was launched on January 22, 2014 in an effort to collect information on bumble bees in Canada and the USA, using a non-lethal survey method. Participants take photos of bumble bees and submit them through a web-interface or app (free for iOS and Android smart devices) along with other information such as the date observed, the geographic coordinates, and optionally, the plant it was observed foraging on. Regional experts then verify the bee identifications, if possible, using identification keys and their own expert knowledge in conjunction with the photograph(s) provided [48]. Plant identifications are not verified. Records from before the program launch date are accepted if all required data are also submitted. Records have been submitted from every province, state, and territory, including remote, rural, suburban, and urban areas [49]. While previous work has been done on the accuracy and breadth of the BBW data [48], and the social impacts on participants [49], it is valuable to see if BBW is contributing new or additional information than that collected by researchers. Previous research has done comparisons of other community science programs to more traditional programs [32,46,50–56]. In many cases, the studies discovered important information on topics such as range expansions and abundances from the community science data and could learn more about changes over time using these data.

The Bumble Bees of North America database (herein “BBNA”) [57] was initially created to collate known information about bumble bees for the Bumble Bees of North America Identification Guide [1]. It is now maintained by Dr. Leif Richardson, and is used by different organizations and individuals for research and conservation purposes. As of April 2019, it had more than 587,000 observations across the United States and Canada, contributed by over 250 individuals or institutions, with records dating back to 180 5(see https://www.leifrichardson.org/bbna.html for a full description of the contributors). Most of these records come from inventorying insect collections or surveying insects in the field (both studies targeting bumble bees specifically or for broader research). Floral information exists for some records but is not a mandatory field; although contributors may have had photographs, these are not kept in BBNA. Data is submitted directly to Dr. Richardson by known regional bumble bee experts or researchers known to experts, often in spreadsheet format. Bee and plant identifications provided in these files are treated as accurate although some quality control reviews are done on new datasets and the whole database (e.g. for records that seem to have erroneous aspects). While not all bumble bee researchers submit their data to this database (or to the Global Biodiversity Information Facility (GBIF), https://www.gbif.org/, which is an international portal for data on all species that BBNA also contributes to and pulls data from), it is the single most populated database for bumble bees in North America. To obtain a copy of all or part of the database, please contact Dr. Richardson at the e-mail addresses provided at https://www.leifrichardson.org/.

Here we ask the following questions to determine the amount of novel information a new community science program can generate as compared to the long-term professional data set:

1. Does BBW show a higher number of species records, richness, or diversity?
2. Does BBW show examples of points outside a species known Extent Of Occurrence?
3. Does BBW show increases in species range sizes?
4. Does BBW show examples of species range infill?
5. Does BBW confirm species presence or persistence?
6. Does BBW provide new forage plant information for bumble bee species?

## Materials and methods

### Dataset exports and cleaning

We obtained an export of all BBNA records (individual bee observations or specimens) (excluding those with permissions prohibiting sharing) on May 28, 2020 (n = 666,194) [57] and all BBW records on August 19, 2020 (both nest and bee records, including those marked private, n = 69,496). After cleaning (see below), a second copy of the BBNA data set was made that only contained records recorded from 2010–2020. This ten year time period was chosen for two reasons: 1) it includes all the years since BBW was launched (and about 99.4% of the verified BBW data in total), so allows for a comparison of data over a similar time period between datasets, and 2) a decade of data is often used in species conservation status assessments (e.g. to determine if a species is in decline)[58,59].

The data were cleaned to remove any records that were not verified by experts (i.e. still pending identification or determined to be non-*Bombus*) (BBW) or that were flagged as having identification problems (BBNA). An exception was one record in BBW (bee id# 33843) that had its identification updated post-export from “unknown species” (status pending) to *B*. *jonellus* (status verified), as it had been identified as such by an expert but at the time that name was not available for use in the database.

We removed all bee records that had missing or imprecise geographic coordinates (e.g. those appearing out in oceans, in the wrong federal jurisdiction as compared to the text field, not in Canada or the United States, etc.). For BBNA, we removed records from community science programs to concentrate the comparison on more traditional researcher data and remove duplicate records. These included BBW, BeeSpotter, BugGuide, iNaturalist, Maine Bumble Bee Atlas, Nebraska Bumble Bee Boosters, Texas Bumble Bees, and the 2012 Xerces Society Citizen Science project. The final datasets included 475,159 records for BBNA all years, 132,001 for BBNA 2010–2020, and 44,134 for BBW.

### Species identification

While the current primary identification guide for bumble bees in North America north of Mexico lists 46 species [1], new species have been described and other taxonomies changed or challenged since that book was published, with no consensus for the new number of species (Table 1 in S1 File). It was outside the scope of this paper to reidentify specimens and update species names, and thus we used the species names as provided in the data obtained.

While BBNA only contained identifications at the species level, some BBW records were only determined to genus by the expert verifiers (i.e. *Bombus* sp., unknown bumble bee species) or were considered as one of two types of species groups: "*vagans*, *sandersoni* or *perplexus*"(two-striped yellow group) and "*vosnesenskii* or *caliginosus*" (yellow-faced group). While some authors removed records from their analyses that were not identified to a specific species (e.g. [54]), we felt that there was still value in including these data points. For example, it shows that at least one of the species in the “group” were present in the area (e.g. for the “two-striped yellow group”, at least one of *B*. *perplexus*, *B*. *sandersoni*, or *B*. *vagans* was present), which is more information than omitting the record completely. Similarly, an unidentified bumble bee record still shows that someone was at that site and saw at least one bee species at that time. Thus we have followed the lead of BBW administrators in keeping the data, and include the three “groups” in our analyses as full “species”.

Finally, although some authors removed records of species that did not have the same corresponding species in the comparison dataset (e.g. [50]), we again kept the points in and used them where applicable. Table 2 in S1 File contains a list of all the species found in this study, their taxonomic authorities, and conservation statuses.

To compare plant forage information, we collated, cleaned, and standardized the information provided in each data set. For example, we converted common plant names to scientific ones to most detailed level possible when scientific names were not provided, fixed spelling errors, and updated synonyms where new ones were known (e.g. joe pye weed, *Eupatorium maculatum* (old) vs *Eutrochium maculatum* (new); crown vetch, *Coronilla varia* (old) vs *Securigera varia* (new)). Taxonomic authorities were generally not provided with the plant names so interpretations involved the most common usages of the names.

### Spatial data

We obtained shapefiles of US and Canada federal jurisdiction boundaries [60,61] merged them into one file, removed any regions from outside the study area, and merged the two Maryland polygons, resulting in 63 total provinces, territories, and states (including District of Columbia but excluding Hawaii, which has no native bumble bees).

We imported BBW and BBNA data into ArcGIS [62] using the geographic projection GCS_NAD83, which we later projected to use GCS_ArcInfo and Sphere Azimunthal. (Note that the original records may have been collected using different projection systems; this information was not provided with the data). Records that fell at or within 5km of the US Canada boundary file were snapped to the nearest point of land; spot-checks using Google Maps and additional data (e.g. site name, city name) confirmed that this seemed to be an appropriate process (records >5km away were removed). Some records had uncertainties noted regarding the exact location (often due to deliberate obscuring of coordinates for privacy or other restrictions), but records were left in when the uncertainty was <100km; however, this does mean that the actual location of some records may not be in the correct 10km x 10km or 100km x 100km grid for analyses in this study.

### Creation of grids

Various scales and methods have been used to determine the number of populations of insects in an area, and for comparisons between datasets, such as by ecoregion, province/state/territory, 100km x 100km grids, 50km x 50km grids, 10km x 10km grids, and 1km x 1km grids, as well as through population genomics [32,50,63–68]. We use here the definition provided in a US Fish and Wildlife Service report [64] that states “A population is a collection of tens to hundreds of colonies, and the health (long- term productivity) of populations is affected by the quantity and quality (a diversity of floral resources) of nectar and pollen available and the proximity of these resources to nesting sites. For monitoring the number of populations over time, a population is a single 10 kilometer (km) x 10km grid.”. The 10km x 10km grid is based on the average foraging and dispersal distances of a bumble bee colony that are usually less than 10 kilometers (although there are exceptions) and the expert knowledge that colonies are often densely concentrated [64,69–74].

**Fig 1:**
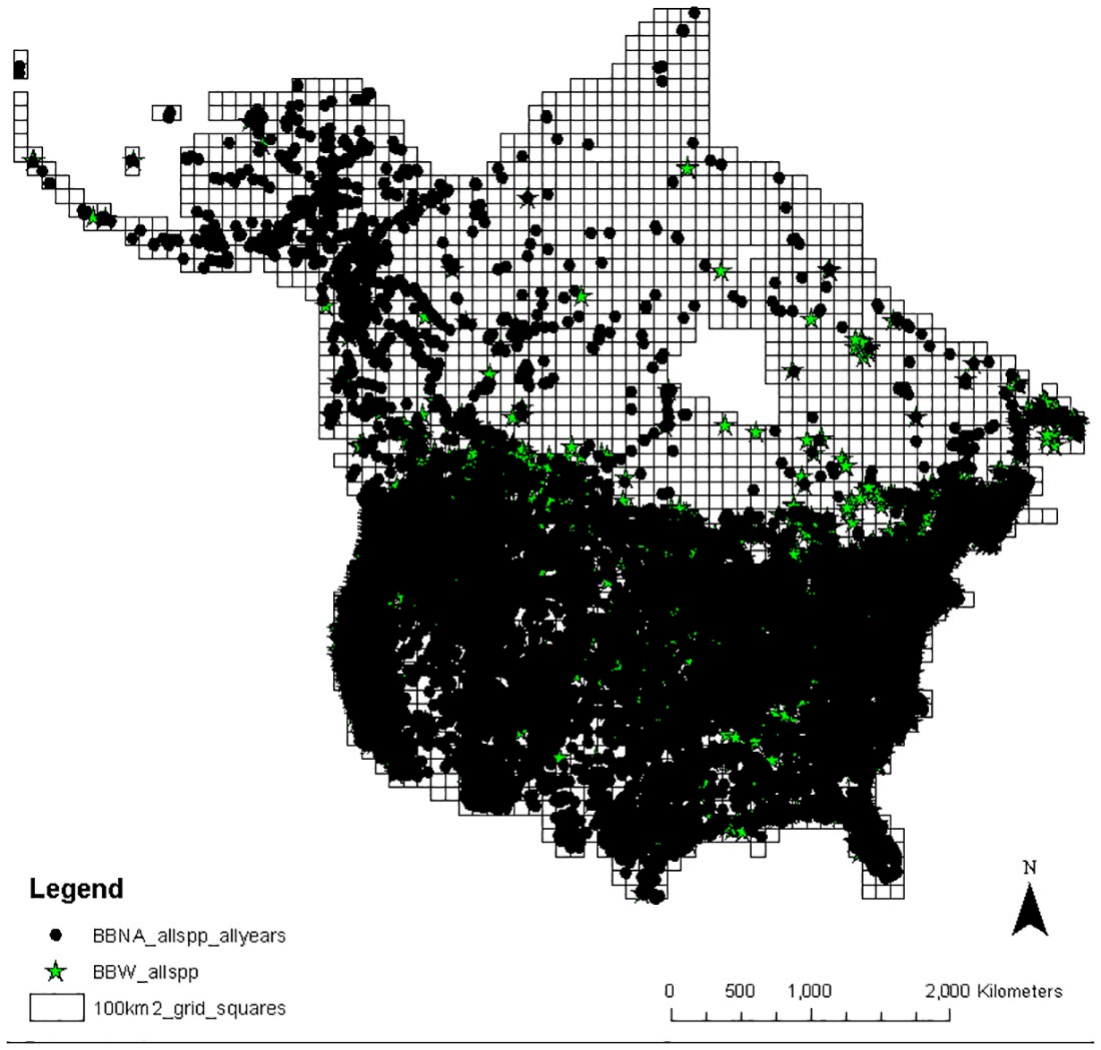
Location of individual BBW records (green stars) and BBNA all years (black circles) on the 100km x 100km grid system applied to a map of Canada and the continental United States. Note that records located at the same or nearby locations may have overlapping symbols, so the number of symbols visible may not equal the number of records. The underlying maps contain information [14,15] licensed under the Open Government Licence–Canada [16] and the United States National Weather Service [17], neither of which are subject to copyright protection.

We concentrated our analyses on the 100km x 100km grid cell to facilitate analyses on the broader North American scale while trying to avoid spatial biases, but also on the 10km x 10km grid cell to capture new populations. Continental wide grids of 10km x 10km and 100km x 100km in size were created using the fishnet polygon option in ArcGIS, and all species records were spatially joined with the relevant boundary files to associate province/state/territory name and 10km x 10km and 100km x 100km grid numbers. “Unique grids”, as used in this paper, refer to those grid cells where only one dataset had records present in the comparison being investigated, as opposed to two datasets (e.g. number of grids where a particular species was only found by BBW vs BBNA all years). See Fig 1 for an example of the 100km x 100km grid with the BBW and BBNA all year and 2010–2020 data points.

### Calculating Extent Of Occurrence, infill, presence, persistence

One way to determine the outer limits of a species geographic distribution is through the application of minimum convex polygons (mcp) around all records in ArcGIS: this area is known as the species Extent of Occurrence (EOO) [12,50,75]. After creating the EOO layers, we then investigated the number of BBW points outside the BBNA all years and BBNA 2010–2020 EOOs by first selecting all BBW points that fell within the BBNA EOO, and then doing an inverse selection: these points show extensions of species geographic range limits due to BBW. The total area of each EOO was also calculated in ArcGIS and the difference between the datasets recorded using MS Excel. Another method of looking at range size per species was calculated by counting the number of 100km x 100km occupied grids in each individual and combined dataset.

The percent infill (i.e. new locations or occurrences within a species geographic range) contributed by BBW was calculated by dividing the number of unique or novel 10km x 10km and 100km x 100km grids contributed by BBW by the total number of grids in the BBNA all years and BBW combined datasets, and the BBNA 2010–2020 and BBW combined datasets, for each species.

To investigate if BBW confirms the presence of a species found by researchers, we looked at the proportion of all 100km x 100km grid cells in the BBNA all years dataset that also had at least one BBW record. For confirmation of recent species persistence (i.e. occurrences in last decade vs historical), we looked at the proportion of 100km x 100km grid cells in the BBNA all years dataset that were not in the BBNA 2010–2020 dataset but had at least one BBW record (i.e. proportion of all BBNA grids with observations in the last decade contributed solely by BBW).

### Statistics

Summary statistics (e.g. totals, means ± se), species richness (standard and Menhinick’s index), species diversity (Shannon-Weiner), and unique values (e.g. number of species, records) were calculated using MS Excel [76] on a per species, province/state/territory, 100km x 100km grid and 10km x 10km grid basis. Species richness is the total number of species but does not consider sample size/sampling effort; Menhinick’s index for species richness helps to take into account sampling effort and sample size and is calculated as the number of species divided by the square-root of the total number of individuals. Species diversity was measured using the Shannon-Weiner Index (H) is calculated as H = -1*sum(pi*lnpi), where pi is the proportional abundance of each species. Note that these statistics excluded zero values/missing species where applicable (i.e. averages and range of numbers of plant genera, plant species, and total grids per species).

Statistical comparisons were carried out using JASP Version 0.14.1 [77]. Independent student t-tests (statistic abbreviated as T), or the non-parametric Mann-Whitney U-tests (U) when assumptions of normality and equal variances could not be met, were used to compare differences in the mean numbers of bee species records, richness, and diversity, sizes of EOOs, number of 100km x100km and 10km x 10km grids, and the number of plant genera and plant species, between BBW and BBNA datasets on various scales. One-sample t-tests, or the non-parametric Wilcoxon signed rank test (W) when the assumption of normality was not met, were used to determine if the mean numbers of BBW points outside of the EOO of species in both BBNA datasets, or the mean numbers of unique 10km x 10km and 100km x 100km grids contributed by BBW per species in both BBNA datasets, were significantly different than zero (i.e. contributed significantly more points or grids overall than zero). Chi-square (χ ^2^) goodness-of-fit tests compared the observed BBW frequency of occupied grids vs the expected BBNA all years or BBNA 2010–2020 occupied grids to see if number of BBW grids was significantly different between the datasets being compared. Statistical tests are significant when p<0.05.

## Results

### 1. Does BBW show a higher number of species records, richness or diversity?

**Fig 2:**
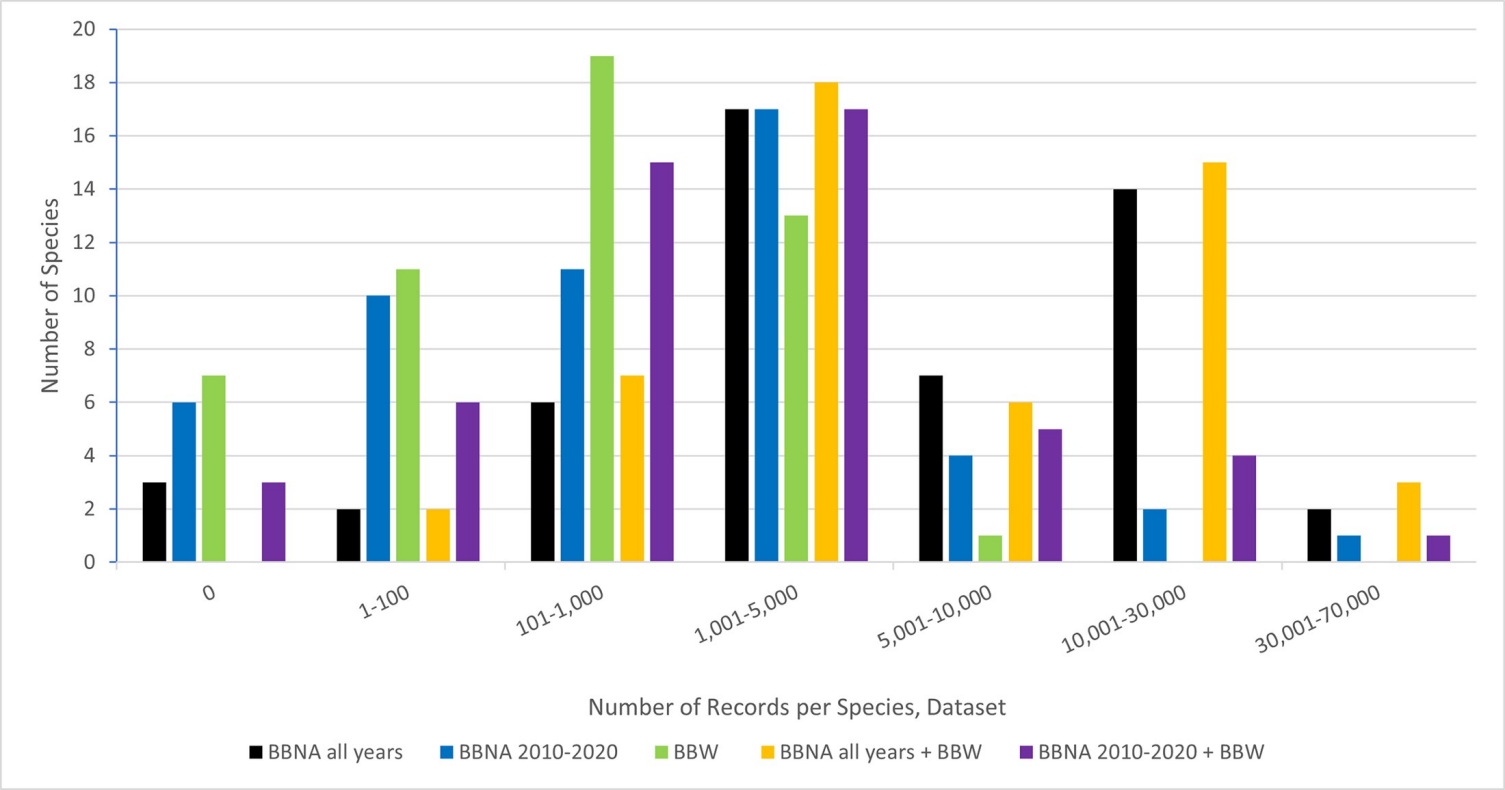
A comparison amongst the number of bumble bee (*Bombus* spp.) species and the numbers of records per species and dataset.

BBW contributed 8.50% of all records (individual bee observations or specimens) in the BBW and BBNA all years combined dataset and 25.06% of all records in the BBW and BBNA 2010–2020 dataset (Table 1 and Table 3 in S1 File). See Table 1 for the total and average numbers of species per dataset, Fig 2 for the number of species per bin of total records, and Table 3 in S1 File for a complete list of records per species and dataset.

**Table 1:** Total number, average number, and species richness of bumble bee (*Bombus* spp.) records from the BBNA all years, BBNA 2010–2020, and BBW datasets, and from the BBW + BBNA all years and BBW + 2010–2020 combined data sets.

*Bombus* species	BBNA all years	BBNA 2010–2020	BBW	BBNA all years + BBW	BBNA 2010–2020 + BBW
**total # records**	475,159	132,001	44,134	519,293	176,135
**average # of records ± se**	9,316.8 ± 1,609.7	2,588.3 ± 714.8	865.4 ± 191.2	10,182.2 ± 1,768.8	3,453.6 ± 876.5
**species richness (excluding species groups)**	48	45	41	48	45
**species richness (including species groups)**	48	45	44	51	48

BBW had 41 full species and 3 species groups of the 51 total species represented across the combined dataset (Table 1, Tables 2 and 3 in S1 File), with the 3 species groups not in either BBNA all years or BBNA 2010–2020. (As BBNA did not record any records where one specific species identification was not recorded, these groups are only found in BBW). BBW had more records for 3 species (the three species groups) than the BBNA all years dataset and 13 species than BBNA 2010–2020 (*affinis*, *appositus*, *auricomus*, *caliginosus*, *crotchii*, *fraternus*, *morrisoni*, *nevadensis*, *vandykei*, and *vosnesenskii*, *plus the three species groups*).

**Fig 3:**
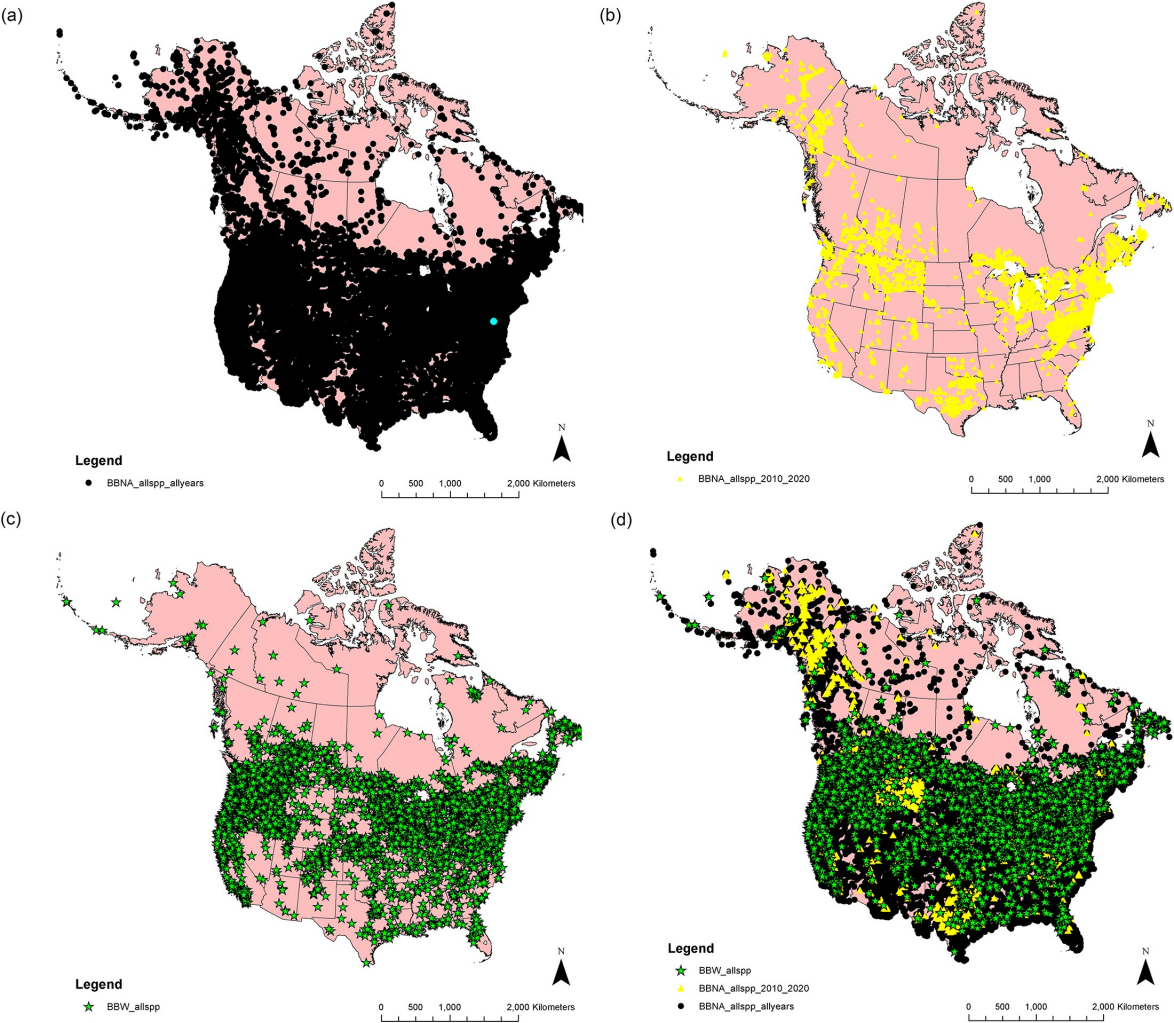
Distribution of bumble bee (*Bombus* spp.) records across North America from A) BBNA all years dataset, B) BBNA 2010–2020 dataset, C) BBW dataset, D) the three datasets together. The underlying map contains information [60,61] licensed under the Open Government Licence–Canada [78] and the United States National Weather Service [79], neither of which are subject to copyright protection.

All 63 provinces, territories, and states were represented in both the BBNA all years dataset and the BBW dataset; however, BBW had four more provinces and states represented than BBNA 2010–2020 (Fig 3, Tables 3 and 4 in S1 File). BBW provided observations of at least one unique species for 60 provinces and states vs BBNA all years and 63 vs BBNA 2010–2020 (Table 4 in S1 File), and more records for one province or state vs BBNA all years and 19 vs BBNA 2010–2020 (Table 5 in S1 File).

Mean values of number of bee records, species richness, and species diversity, were significantly greater in BBNA all years than BBW for all comparisons, except for number of bee records and Menhinick’s Index on the province/state basis, where BBW was greater, and species diversity, where there was no significant difference (Tables 5–7 in S1 File, Table 1 in S2 File). Mean values of BBW were significantly greater than BBNA 2010–2020 for all comparisons except the number of bee records per province/state and 10km x 10km grid basis, where BBNA 2010–2020 was greater, and Menhinick’s Index or species richness on the 100km x 100km grid and province/state basis, respectively, where there was no significant difference (Tables 5–7 in S1 File, Table 1 in S2 File).

### 2. Does BBW show examples of points outside a species known Extent Of Occurrence?

**Fig 4:**
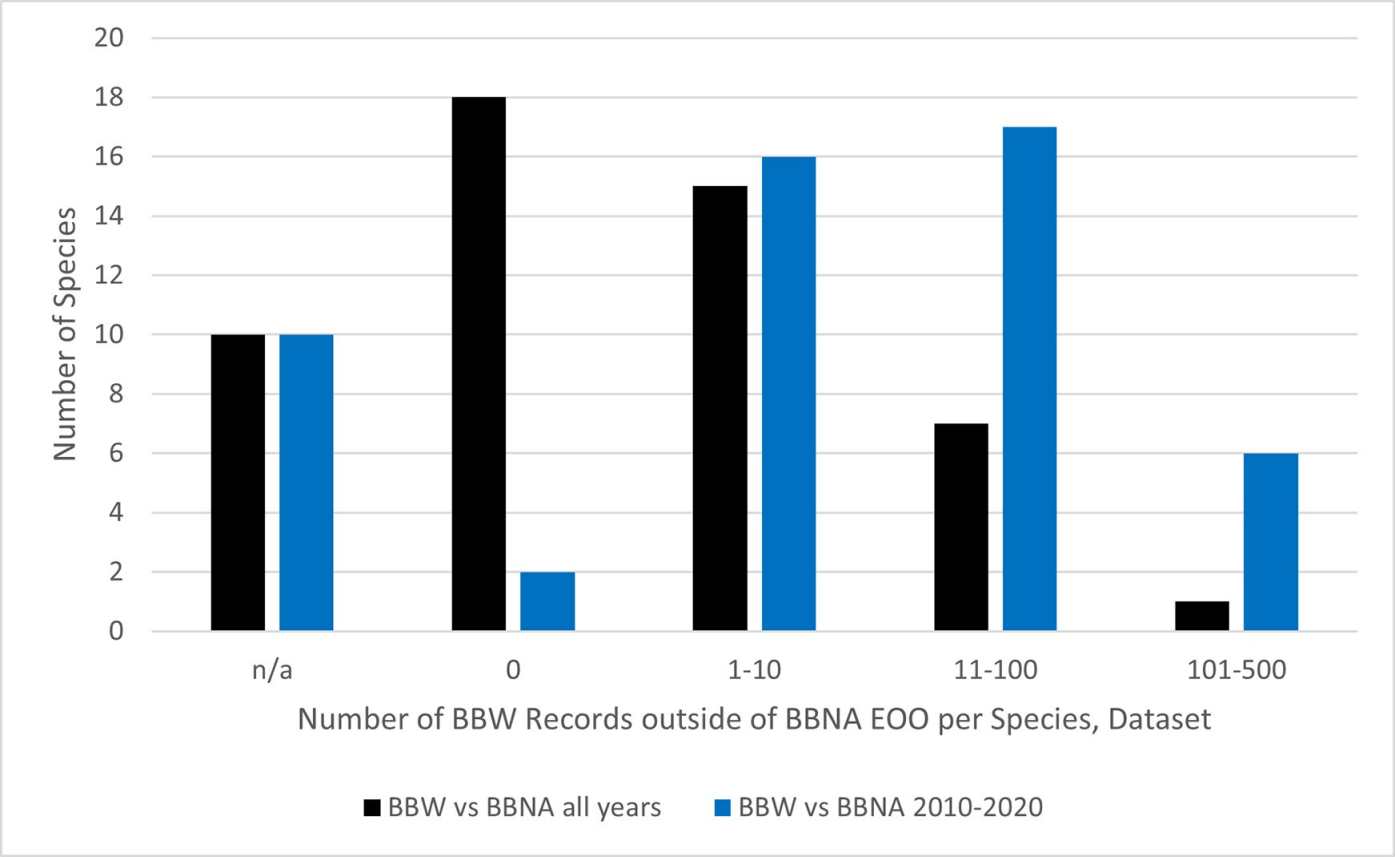
A comparison amongst the number of bumble bee (*Bombus* spp.) records per species from the BBW dataset found outside of the BBNA all years and BBNA 2010–2020 Extent of Occurrence (EOO).

On a per species basis, 23 of the 41 species had records (individual bee observations) in BBW outside of the corresponding BBNA all years species EOO, ranging from 1 record (several spp.) to 121 records (*B*. *impatiens*) (Fig 4 and Fig 1 in S1 File, Table 12 in S1 File). When compared to the BBNA 2010–2020 data, 39 of 41 species had records in BBW outside of the corresponding species EOO, ranging from one record (several spp.) to 456 records (*B*. *vosnesenskii*) (Fig 1 in S1 File, Table 8 in S1 File). The mean number of BBW records outside of each species EOO is significantly different than 0 for BBNA all years (n = 41, mean = 9.439 ± 3.716 SE, W = 276.000, p<0.001) and BBNA 2010–2020 (n = 41, mean = 48.366 ± 13.787 SE, W = 780.000, p<0.001).

### 3. Does BBW show increases in species range sizes?

While BBW had a greater EOO than BBNA 2010–2020 for 15 species individually, there was no significant difference in EOOs between BBW and BBNA 2010–2020 in general (U = 1048, p = 0.055) (Table 9 in S1 File). There were no species in BBW with a greater EOO than in BBNA all years; indeed, BBNA all years EOOs are significantly greater than BBW EOOs (U = 1322, p<0.001)(Table 9 in S1 File). The difference in EOOs were significantly larger in the BBW minus BBNA all years comparison than the BBW minus BBNA 2010–2020 one (U = 426.5, p<0.001)(Table 9 in S1 File). The total EOO values for each species in each dataset, means ± standard error, and the difference between them, can be found in Table 9 in S1 File.

The average number of 100km x 100km grids with records per species, per dataset, when zeros are excluded, was 224.38 ±21.22, 74.40 ± 8.32, and 98.66 ± 14.13 for BBNA all years, BBNA 2010–2020, and BBW, respectively. BBW did not meet the expected frequency distribution of grids per species as compared to BBNA all years (χ^2^ = 1249.579, df = 40, p<0.001) or BBNA 2010–2020 (χ^2^ = 857.386, df = 40, p<0.001) on the 100km x 100km grid scale.

The approximate range size per species, based on occupied 100km x 100km grids, can be calculated from Table 10 in S1 File (total number of grids need to be multiplied by 10,000km^2^ to get the area). While the BBNA all years dataset always provided significantly larger range sizes (i.e. more occupied grids) than BBW (except for the three species groups only in BBW) (U = 1947, p<0.001), BBW did provide a larger range size for 24 species (plus three species groups) as compared to BBNA 2010–2020 (although this was not significant overall, U = 1232, p = 0.649) (Table 10 in S1 File). Similar trends were found for the 10km x 10km grids (U = 1937, p<0.001 and U = 1173, p = 0.395, respectively) (Table 11 in S1 File).

### 4. Does BBW show examples of species range infill?

**Fig 5:**
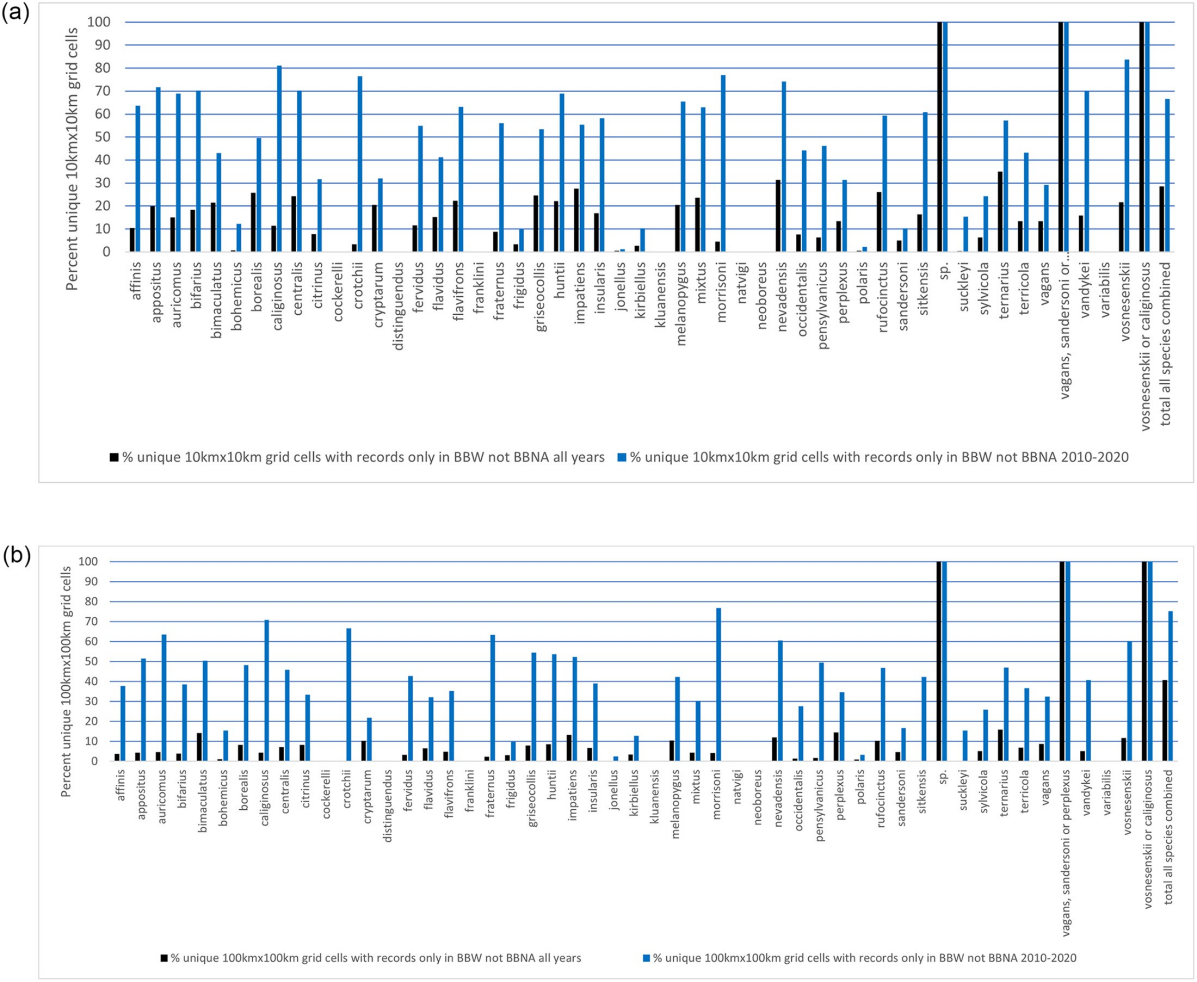
Percent unique A) 10km x 10km and B) 100km x 100km grid cells where records are only found in BBW and not in BBNA all years (black) or BBNA 2010–2020 (blue) datasets, per species. See Tables 10–11 in S1 File for the number of records per species used to calculate these values.

We found multiple examples of BBW infilling the known range of up to 44 species, both on the 10km x 10km and 100km x 100km grid square bases, and especially when compared to the BBNA 2010–2020 dataset (Table 2, Tables 10–11 in S1 File; Fig 5). While it was variable on a per species basis, the maximum number of unique grids contributed by BBW ranged from 1447 and 1524 10km x 10km grids when compared to BBNA all years and BBNA 2010–2020, respectively (no significant differences, U = 1217, p = 0.578), and 419 100km x 100km grids when compared to each of the two BBNA datasets (significantly more unique grids over the 2010–2020 time period than all years (U = 821.5, p = 0.001)) (Table 2, Tables 10–11 in S1 File; Fig 5). The mean number of unique grids contributed by BBW per species (n = 51) was significantly greater than 0 for both scales and dataset comparisons (Table 2).

**Table 2:** Comparison of the 10km x 10km and 100km x 100km grids with records that are unique to BBW as compared to BBNA all years and BBNA 2010–2020 datasets.

Combined datasets for comparisons	10km x 10km grids		100km x 100km grids	
	BBNA all years (n = 15,727) + BBW (n = 6,169)	BBNA 2010–2020 (n = 3,464) + BBW (n = 6,169)	BBNA all years (n = 1,434) + BBW (n = 842)	BBNA 2010–2020 (n = 619) + BBW (n = 842)
**Total # grids in combined data**	19,314	8,922	1,484	1,016
**Number (%) of completely unique grids from BBW**	3,587 (18.57%)	5,458 (61.17%)	50 (3.37%)	398 (39.13%)
**Number (%) of grids unique for at least one species from BBW**	5,513 (28.54%)	5,948 (66.67%)	604 (40.70%)	764 (75.12%)
**Number of species with at least one unique grid from BBW**	44	44	40	44
**Average number ± SE unique grids for all species overall (including 0’s)**	237.529 ± 42.939	274.569 ± 48.193	27.039 ± 8.597	59.741 ± 10.168
**Statistical test result**	W = 990.000, p<0.001	W = 990.000, p<0.001	W = 820.000, p<0.001	W = 990.000, p<0.001

n = number of grid cells per original uncombined dataset. “Species” includes both individual species and species groups (i.e. “sp", "vagans, sandersoni or perplexus", "vosnesenskii or caliginosus"). The first average presented is the average of all grids per species, calculated based on averages per species excluding grids with no unique records; the second average looks at the total number of grids per species, while including species with no unique grids. W = Wilcoxon signed rank test statistic.

### 5. Does BBW confirm species presence or persistence?

**Fig 6:**
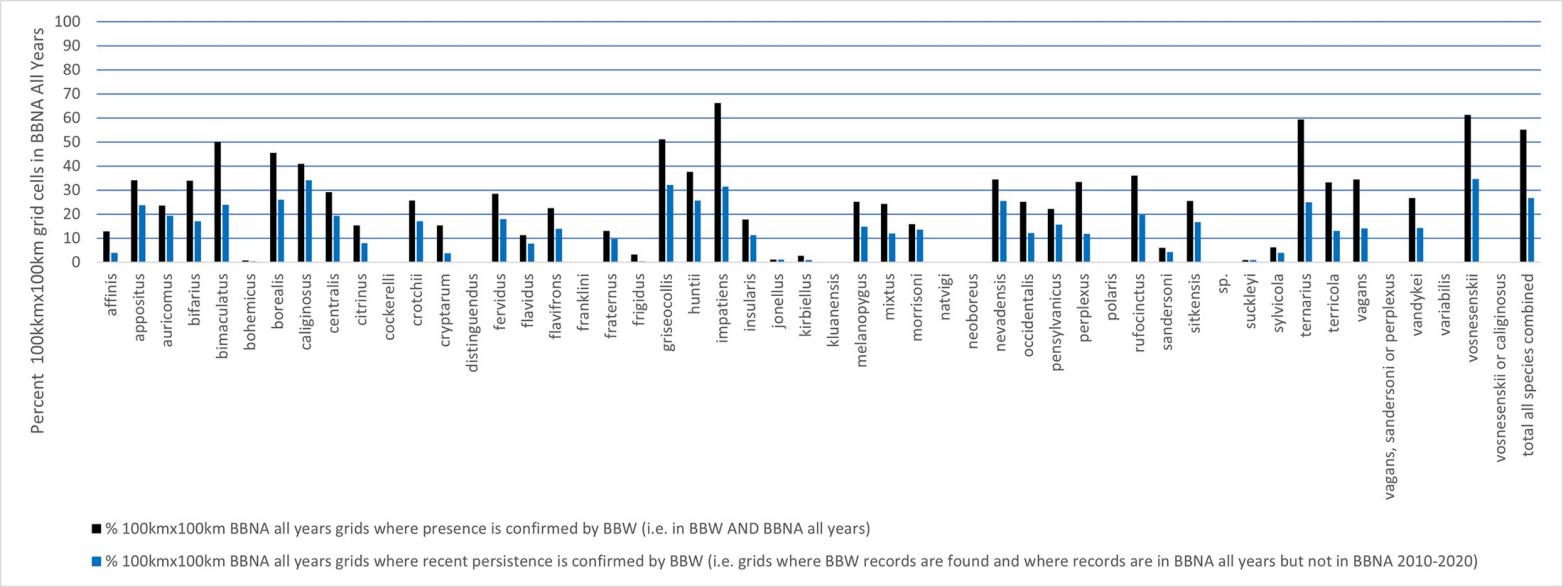
Percent of the BBNA all years occupied 100km x 100km grids where BBW shows confirmation of each species presence (dark blue bars) or unique persistence of each species in the last decade (light purple bars) (i.e. grids where a record was found in both BBNA all years and in BBW and grids where a record was not found in BBNA 2010–2020 but was found in BBW and BBNA all years, respectively). See Table 8 in S1 File for the number of records per species used to calculate these values.

BBW confirmed the presence of at least one species in 717 of the 1434 100km x 100km grid cells that had records in BBNA all years (50.00%), with an average of 26.65% ± 2.72% of the grids containing records on a per species basis (Fig 6, Table 10 in S1 File). It uniquely confirmed the recent persistence of a species (i.e. found in BBNA all years but not BBNA 2010–2020) in 543 of the 1434 100km x 100km grids (37.87%), with an average of 15.34% ± 1.55% of the grids with records per species (Fig 6, Table 10 in S1 File). The mean number of these 100km x 100km grids is significantly different than zero, even when species with no new grids are included (presence mean = 58.078 ± 9.358, W = 820.000, p<0.001; persistence mean = 32.431 ± 5.135, W = 820.000, p<0.001).

### 6. Does BBW provides new forage plant information?

Although BBW had much fewer records than BBNA all years or BBNA 2010–2020 (Table 1), it provided more total plant genera (Table 3) and plant species (Table 4) than either of the BBNA datasets. While this was not significant in the all years comparison using the total numbers per bee species (plant genera: U = 1194.500, p = 0.480; plant species: U = 1235.000, p = 0.663), it was significant for the 2010–2020 comparison (plant genera: U = 864.500, p = 0.003; plant species: U = 894.500, p = 0.007). Indeed, 26.24% and 31.56% of the plant genera (Table 3), and 36.84% and 56.56% of the plant species (Table 4) were only provided by BBW records when compared to the BBNA all years and BBNA 2010–2020 datasets, respectively. The numbers of total and unique records per plant genera and plant species varied per bee species, but 42 bee species in the BBW and BBNA all years and 43 bee species in the BBW and BBNA 2010–2020 datasets had unique plant forage information provided by BBW (Tables 3 and 4, Figs 2–3 in S1 File). A full list of all plant species names per bee species, and numbers of bee records for each, is available in Table 10 in S1 File for all datasets combined.

**Table 3:** A comparison of the total number of plant genera per data set and data set comparison, and records for the same.

Data evaluated	Calculation	BBNA all years	BBNA 2010–2020	BBW	BBNA all years + BBW	BBNA 2010–2020 + BBW
**all bee species combined**	**# total plant genera**	652	414	653	884	735
**all bee species combined**	**# (%) unique plant genera from BBW**	n/a	n/a	n/a	232 (26.24%)	231 (31.56%)
**all bee species combined**	**average # records per plant genera**	127.81 ± 22.48	116.78 ± 19.72	55.05 ± 6.70	134.94 ± 19.88	114.69 ± 15.87
**separate bee species**	**# bee species with unique plant genera from BBW**	n/a	n/a	n/a	42	43
**separate bee species**	**range in # plant genera per bee species**	2–312	1–228	1–330	2–449	1–391
**separate bee species**	**average # plant genera per bee species**	82.57±10.16	48.58±7.60	109.35±12.55	133.27 ± 14.79	118.52 ±13.74
**separate bee species**	**range in # unique plant genera from BBW**	n/a	n/a	n/a	1–308	1–308
**separate bee species**	**avg # unique plant genera from BBW**	n/a	n/a	n/a	65.17 ± 8.73	78.21 ± 9.41

Note: the number of plant genera include the total number of plants identified to the Genus level; when that was not possible, the next closest, higher, equivalent (e.g. family) were recorded. These numbers exclude non-plant forage visitation records (e.g. flying by, sitting on a leaf). Average and range values exclude plant genera where no records were present for each bee species.

**Table 4:** A comparison of the total number of plant species per data set and data set comparison, and records for the same.

Data evaluated	Calculation	BBNA all years	BBNA 2010–2020	BBW	BBNA all years + BBW	BBNA 2010–2020 + BBW
**all bee species combined**	**# total plant species**	1699	920	1755	2688	2117
**all bee species combined**	**# (%) unique plant species from BBW**	n/a	n/a	n/a	991 (36.84%)	1198 (56.56%)
**all bee species combined**	**mean # records per plant species**	137.04 ± 18.87	72.72 ± 13.03	176.07 ± 22.93	240.41 ± 30.40	202.33 ± 26.83
**separate bee species**	**# bee species with plant species information**	46	43	43	49	46
**separate bee species**	**# bee species with unique plant species from BBW**	n/a	n/a	n/a	42	43
**separate bee species**	**range in # plant species per bee species**	2–599	1–413	1–592	2–936	1–788
**separate bee species**	**mean # plant species per bee species**	137.04 ± 18.87	72.72 ± 13.03	176.07 ±22.93	240.41 ± 30.40	202.33 ±26.83
**separate bee species**	**mean # unique plant species from BBW**	n/a	n/a	n/a	1–558	1–558
**separate bee species**	**mean # unique plant species from BBW**	n/a	n/a	n/a	130.45 ± 17.94	143.77 ± 19.05

Note: Number of plant species include the total number of plants identified to the species level. When that was not possible, the next closest, higher, equivalent (e.g. Genus) were recorded. These numbers exclude non-plant forage visitation records (e.g. flying by, sitting on a leaf). Means ± SE and range values exclude plant species where no records were present for each bee species.

## Discussion

Community science programs have immense potential to contribute to scientific understanding and wildlife conservation efforts. After just six years of operation, Bumble Bee Watch (BBW) has demonstrated its value for bumble bee conservation. While the total number of bee records and species contributed by community scientists is not higher than those of researchers to the BBNA database overall, the records are still filling critical knowledge gaps, particularly in recent years and for specific species. Species richness and diversity were often higher in BBW for most analyses. While the overall BBW range sizes for species were usually smaller than from BBNA, often BBW records were found outside of the BBNA range. BBW also increased the number of known populations for species inside of their ranges and vastly increased the number of forage plant genera and species known to be visited. These records all represent unique occurrences in space and time that will never occur again and cannot be recreated. Not only can they be used to help answer questions now when there is an urgent need for pollinator data [80], the data can be used indefinitely into the future, as new questions are always developed.

The underlying foundation of BBW is the ability to use photos for insect identification. With the increase in photo quality and widespread use of digital photography, as well as encouragement of non-lethal data collection, the identification of species by photo is becoming more common in both community science and in the greater academic fields. The usage ranges from photos confirming an observation, as with BBW, through to using photos in lieu of the traditional physical “type” specimen (although the latter is currently accepted only in certain specific rare situations) [81]. However, there are some drawbacks to using photos. For example, the validity of this method does depend on the species being identified (such as visibility of key identifying features), the angles shown in the photo(s), and the quality of the photo(s) (e.g. lighting, focus) [48,52,81–83]. A challenge with the BBW program is that cryptic species cannot be identified from photos alone, particularly the type and quality submitted by community scientists (e.g. from a distance on a flower vs under a dissecting microscope).

The number of extant species in the genus *Bombus* has changed over time, which can explain why some species did not appear in one dataset or the other. Many of the recent species changes in the genus *Bombus* are a result of DNA barcoding and re-examination of morphological characters, particularly for cryptic species [1,84–88]. Some of these new species do appear in the BBNA datasets (e.g. *B*. *kluanensis* and *B*. *natvigi*), but not all (e.g. *B*. *johanseni*); none were found in BBW. It is likely that some of these species, particularly those in the *Alpinobombus* sub-genus, have been submitted to BBW but are still pending verification or verified as an unknown species due to the difficulty in assigning an identification [48]. It may be useful for BBW administrators to follow up with the submitters of these specific and uncommon records, to ask them to gain a physical voucher specimen or portion thereof (e.g. antenna or tarsal (final segment in the leg of the insect) clip) of a similar specimen to the one photographed for a researcher to identify in person or through DNA barcoding, and/or add in more species groups to account for these northern cryptic bees. While this would not be expected to be a full solution to the problems associated with identifications by photo, and specific instructions would need to be given, it would help with some identifications, and increase our knowledge of species in these regions. In addition to the northern bees that are both difficult to identify and found in areas with fewer participants, other species missing from BBW but present in BBNA (e.g. *B*. *franklini*, *B*. *variabilis*) are those that are likely extirpated or very uncommon [1,48]. With these caveats, it can be said that BBW generally has captured all the confirmed and extant species for North America.

Our results were consistent with previous findings that while the total numbers of community science records may or may not be lower than those from expert databases, it often makes up for it with more species per 1,000 observations [54] and/or observations in areas unique only to the community science databases, in some cases in areas unexpected by researchers [32,50,54]. Indeed, opportunistic observations can help to document new species, locations of species and/or changes in range, whether through natural or human-mediated causes [32,50,54,63,83,89,90]. As climate change is a major component, affecting range expansion and contraction for many species, including bumble bees [91–96], this information will be increasingly important.

BBW has shown its value in detecting species at-risk of extinction. The BBW dataset provided observations and unique populations (unique presence in 10km x 10km grids versus BBNA) for 11 at-risk and 5 data-deficient species over both the BBNA all years and 2010–2020 datasets. As an example, *Bombus affinis* and *B*. *occidentalis* have become the “poster-children” for bumble bee declines in eastern North America, and many members of the public are keen to find them, using BBW as the tool to identify the bees they see [48,49]. This has resulted in more populations becoming known to researchers and allowing for more specialized research.

Another notable example was the discovery of *B*. *bohemicus* in eastern Canada in 2020 by a BBW participant at two areas in Rimouski, Quebec. This species is assessed as Endangered in Canada (COSEWIC [97], Endangered in Ontario [98], and Critically Imperiled across most of its range but Possibly Extirpated in Quebec [99]. Previously, the last observations of this species in Ontario and Quebec, as represented in the BBNA all years dataset, were in 2008 despite extensive surveys by researchers. Without community science programs, these occurrences may have gone unrecorded and the species was closer to being deemed extirpated in these regions.

In this paper, a focus was made on comparing BBW to two different BBNA datasets. to evaluate the contributions of BBW overall and over a similar temporal scale. The results clearly showed that while BBW contributed novel information compared to the all years BBNA dataset, its real power was in the higher amount and type of information contributed over the 2010–2020 time period.

BBW did not have any species not also documented in BBNA; this is not surprising due to the greater number of records and geographic coverage in the BBNA all years dataset, and the difficulties identifying cryptic species solely by photo. However, BBW did have more records for 13 species than BBNA 2010–2020, including five at-risk species (*affinis*, *caliginosus*, *crotchii*, *fraternus*, and *morrisoni*) and one species group that contains an at-risk species (*vosnesenskii or caliginosus*). For all species combined, BBW reported observations in 50 and 398 unique 100km x 100km grids not in BBNA all years and 2010–2020, respectively, with many (2–126) unique 100km x 100km grids for 8 at-risk species compared to BBNA all years and 10 at-risk species for 2010–2020. BBW also provided records for 23 species outside of their known distribution as calculated through the BBNA records.

These examples confirm the value of community science data for determining species presence and persistence, particularly for at-risk species and in areas that may not be known to or surveyed by researchers [32,50,100,101]. It also validates the value of community science being for documenting species distributions, including changes in range, similar to or beyond that of traditionally collected data [19,32,50,54,56,102,103] (but also see [104]). Other examples beyond BBW include how the range size estimations of three saproxylic beetle species from two years of community scientist records in the European LIFE Project, “Monitoring of Insects with Public Participation”, was approximately equal to ten years of a formal national inventory data [32], new distribution information was provided for over 80% of the butterfly species in Canada by community scientists with eButterfly [50], and the inclusion of eBird records helped improve predicted species occurrence models for seven of twelve rare birds in Canada [103].

Biases in community science data include uneven geographical distribution (e.g. urban centres or along roadsides), overrepresentation of particular species (e.g. a focus on documenting rare or unusual species), variation in detectability of species (e.g. influenced by skill of the observer, quality of habitat, weather conditions, time of day, size of population, period of colony growth cycle [16,19,48,104–108]. These biases, and differing patterns of data collection between different surveyors, can occur in both community science data and in other data (e.g. museum collections, researcher surveys), and should be controlled for when possible; results should be treated with care and evaluated for an effect of the bias(es) [8,16,19,104,106,108–110]. However, biased data can still be used to complement less biased, more standardized data collection methods; indeed, the strengths of one can offset the weaknesses of others [19,104,106,108,111,112].

Both of the BBW and BBNA datasets may contain biases related from the surveying/recording/collecting (or non-recording/collecting) of rare species vs common species, as well as biases related to geography, the expertise of identifiers, ease of differentiation of species, etc. (see discussion above and [16,113]). Findings often need to be interpreted with care, and indeed, data sources and methods reviewed prior to use. Soroye et al. [50] cautions that, when other more suitable data exists, it may be better to not use opportunistic data in analyses related to species or regions, especially when it makes the analyses more complex. As well, more data does not always mean more power for analyses, and are not always better if they introduce more uncertainly into analyses or obscure results [50]. In some cases, only part of the data may not be suitable for use; for instance, Graves et al. [114] excluded BBW data from the BBNA dataset used in their models as the former database is generally composed of incidental or opportunistic observations as compared to complete inventories.

Opportunistic data can be used in many analyses, and indeed, in some cases (with particular species, regions, and/or methods) can provide more reliable results than other types of formal surveys, such as with bumble bees in areas where the study area is small and historical baseline data are available for comparisons [115]. At the very least these data can confirm historic observations and yield new locations or other novel information, as well as complementary and confirmatory data [32,50,56,101].

By adding together existing datasets, such as BBNA and BBW, and by carefully designing both traditional research methods and community science programs, researchers and conservationists can potentially achieve a greater understanding and closer approximation of “real” conditions [15,16,46,50,56,90,114,116]. BBW data can be viewed in real-time via the website or app platforms, and exports can be requested via the program’s data request form. The data are also routinely accepted into the BBNA database and the USGS BISON (Biodiversity Information Serving Our Nation) database (https://bison.usgs.gov), and has been added to other national and regional databases, so the data are increasingly becoming available to researchers.

BBW data has already been used by researchers for species conservation assessments [74,100,117] and regional checklists [118], and by land managers and conservation professionals to identify sites for habitat conservation, restoration, and linkage efforts, or for further research or advocacy efforts [48,100,119,120]. Similarly, data from other community science programs have helped assess the distribution, diversity, and environmental associations of bumble bees and other bee species [52,53,116,121,122]. As these datasets grow and are compiled with others, many more future uses will be likely. As stated in McKinley et al [15]: “The best science does not necessarily come from the best peer-reviewed scientific publications with the most robust designs and inferences; rather, it is the best scientific information available to answer a specific question”.

For effective wildlife conservation, we need to consider the whole spectrum of actions and tasks, from understanding the current status of a species population or conservation target to identifying and understanding the underlying drivers of the issues to describing the actions needed to support the species and testing the impacts and responses of those actions [13,14,23,116]. We also need to raise public awareness of these species or targets and their interest in supporting and meeting them, as this increases the probability of actions actually happening by the public, conservation groups, and governments [14,123–131].

Community science programs can help with these needs, as their participants are involved in collecting data and/or answering questions with that data, learning about how science works to answer questions and about the specific study focus, while gaining more awareness of the current status, threats, and needed actions, often getting reconnected with nature in the process [15,18,29,32,56,83]. This awareness and connectedness is achieved not only through hands on experiences but also through education components associated with the program; indeed, other members of the public also gain exposure to these topics through general media coverage and program outreach activities, even if they are not participants themselves [15,18,32,34,83,132]. Participants and the public in turn provide feedback to community science organizations and conservation professionals about their priorities and understandings [15,18,83,133–135]. Past Bumble Bee Watch participants have noted the positive impacts the program has had on them, including their increased knowledge and willingness to take action [49], and this may lead to more public support for broader actions in the future.

In conclusion, our findings show the potential for community scientists to contribute to scientific knowledge and conservation management. As noted by Ellwood et al (2017), the intersections of community science and conservation can help address the large challenges we face. In this example, Bumble Bee Watch benefits from the public’s desire to learn more about and protect pollinators [49,124], and researchers gain valuable data that would not have been possible otherwise. Coupled with the widespread availability of high-quality images and technology provided by digital cameras, smart devices, and the internet, the potential for this and other community science data collection programs to help mitigate species decline and other environmental challenges will grow in the future.

## Supporting information

S1 File Supplementary Information File 1 Contains Tables 1–11 in S1 File and Figs 1–3 in S1 File. (DOCX)

S2 File Supplementary Information File 2 Contains Tables 1–3 in S2 File. (XLSX)
