# Supplementary material for: Bumble Bee Watch community science program increases scientific understanding of an important pollinator group across Canada and the USA
Source: PLoS One. 2024 May 22;19(5):e0303335. doi: 10.1371/journal.pone.0303335 (PMC11111064; doi:10.1371/journal.pone.0303335)
Supplement: S1 File — (DOCX) [file pone.0303335.s001.docx]

**Supplementary Information File 1 for “Bumble Bee Watch community science program increases scientific understanding of an important pollinator group across Canada and the USA”**

**by Victoria J. MacPhail, Richard Hatfield, Sheila R Colla, PLOSOne 2024**

Note: this is the first of two supplementary files. It excludes S2 File Tables 1-3; please see the Supplementary Information File 2 (Excel file) for those data.

S1 File Table 1. Current Bombus species that are considered as valid species for North America north of Mexico but that were not included in Williams et al. (2014). These species are newly described, recently re-validated, or still a point of disagreement for taxonomists.

S1 File Table 2. The common and scientific names and taxonomic authorities for the 51 species and groups of bumble bees (*Bombus* spp.) found in either the BBNA all years or BBW datasets, as well as their IUCN Red List or NatureServe conservation status for their North American range.

S1 File Table 3. Total number of bumble bee (*Bombus* spp.) records per species from BBNA all years, BBNA 2010-2020, and BBW datasets, and from the BBW + BBNA all years and BBW + 2010-2020 combined data sets.

S1 File Table 4. The number of total and unique bumble bee species (including three species groups: "sp", "vagans, sandersoni or perplexus", and "vosnesenskii or caliginosus") per province, state, and territory, by dataset.

S1 File Table 5. The total number of bumble bee records (all species combined) per province, state, and territory by dataset.

S1 File Table 6. A comparison of the mean numbers of bee records, species richness, Menhinick’s Index, and Shannon-Weiner species diversity index values in BBW as compared to BBNA all years and BBNA 2010-2010 at three scales: province (including states and territories)(n=63), 100km x 100km grids (n=1485), and 10km x 10km grids (n=19314).

S1 File Table 7. The total Menhinick’s and Shannon-Weiner diversity indices (all species combined) per province, state, and territory, by dataset

S1 File Table 8. A comparison of the number of BBW records that fall outside of the Extent of Occurrence (EOO) for each bumble bee (*Bombus*) species in the BBNA all years and BBNA 2010-2020 datasets.

S1 File Table 9. A comparison of the Extent of Occurrence (EOO) geographic range area and difference in EOO areas per species between BBW dataset and the BBNA all years or BBNA 2010-2020 datasets.

S1 File Table 10. A comparison of the number of 100km x 100km grids: with records per species per dataset; where BBW confirms the presence of a species in BBNA all years (i.e. in both BBNA and BBW); where BBW shows novel recent persistence (i.e. present historically in BBNA all years but only found in the last decade through BBW); with unique grids over the combined all years and 2010-2020 datasets due to the BBW dataset; and the total number of girds in the combined BBNA all years + BBW and BBNA 2010-2020 + BBW datasets.

S1 File Table 11 A comparison of the number of 10km x 10km grids with records per species per dataset, and the number of unique grids over the combined all years and 2010-2020 datasets due to the BBW dataset.

S1 File Fig 1. Location of individual BBW records (green stars) as compared to the Extent of Occurrence area (EOO) for BBNA all years (black grid, left map) and BBNA 2010-2020 (blue grid, right map), for each of the 41 bumble bee (Bombus) species (Figs S1:a-pp) that were present in all three datasets. Note that records located at the same or nearby locations may have overlapping stars, so the number of stars visible may not equal the number of records. The underlying maps contain information [1,2] licensed under the Open Government Licence – Canada [3] and the United States National Weather Service [4] , neither of which are subject to copyright protection.

S1 File Fig 2. A comparison of A) the total number of plant genera recorded per bumble bee species in BBNA all years (black bars), BBNA 2010-2020 (yellow bars), and BBW (green bars) datasets, and B) the percent unique plant genera recorded from BBW as compared to BBNA all years (blue bars) and BBNA 2010-2020 (orange bars).

S1 File Fig 3. A comparison of A) the total number of plant species recorded per bumble bee species in BBNA all years (black bars), BBNA 2010-2020 (yellow bars), and BBW (green bars) datasets, and B) the percent unique plant species recorded from BBW as compared to BBNA all years (blue bars) and BBNA 2010-2020 (orange bars).

**S1 File Table 1. Current *Bombus* species that are considered as valid species for North America north of Mexico but that were not included in Williams et al. (2014)**.

| ***Bombus* species** | **Status in Williams et al. (2014)** | **Reference** |
| --- | --- | --- |
| ***californicus* Smith, 1954**  **(but this species status is still uncertain)** | was considered a conspecific of *B. fervidus* (Fabricius, 1798)  but note that although Koch et al. (2018) found evidence for two lineages in this complex, they could not assign valid names due to the cryptic nature of the bees and the original descriptions | Koch et al. [5] |
| ***cockerelli* Franklin, 1913**  **(but this species status is still uncertain)** | was considered a conspecific of *B. vagans* Smith, 1854 | Yanega [6] |
| ***interacti* Martinet, Brasero and Rasmont, 2019**  **(but this species is now considered to be a synonym)** | not included (new species)  but now considered as conspecific of *B. johanseni* Sladen, 1919 by Sheffield et al. [7] | Martinet et al. [8] |
| ***johanseni* Sladen, 1919** | not included | Sheffield et al. [7] |
| ***kirbiellus* Dahlbom, 1832** | was considered a conspecific of *B. balteatus* Dahlbom, 1832 | Williams et al. [9] |
| ***mckayi* Ashmead, 1902 STAT. REV.** | was considered a subspecies of *B. occidentalis* s. str.  now split to be a separate species (as had originally been described) | Williams et al. [10] |
| ***natvigi* Richards, 1931** | not included | Williams et al. [9] |
| **unnamed species**  **=**  ***kluanensis* Williams and Cannings, 2016** | not included (new species) | Williams et al. [9]  Williams et al. [11] |
| ***vancouverensis* Cresson, 1878** | *B. bifarius* was listed with two possible subspecies; it was split into two species by Ghisbain et al. (2020), and then one, *B. nearcticus* was then itself split into two subspecies, *B. vancouverensis vancouverensis comb.n.* and *B. vancouverensis*  *nearcticus* | Ghisbain et al. [12] |

These species are newly described, recently re-validated, or still a point of disagreement for taxonomists.

**S1 File Table 2. The common and scientific names and taxonomic authorities for the 51 species and groups of bumble bees (*Bombus* spp.) found in either the BBNA all years or BBW datasets, as well as their IUCN Red List or NatureServe conservation status for their North American range.**

| **Species** | **Authority** | **Common Name** | **IUCN Status [NatureServe Status]** |
| --- | --- | --- | --- |
| ***affinis*** | Cresson, 1863 | Rusty-Patched Bumble Bee | CR |
| ***appositus*** | Cresson, 1878 | White-Shouldered Bumble Bee | LC |
| ***auricomus*** | (Robertson, 1903) | Black and Gold Bumble bee | LC |
| ***bifarius*** | Cresson, 1878 | Two Form Bumble Bee | LC |
| ***bimaculatus*** | Cresson, 1863 | Two-spotted Bumble Bee | LC |
| ***bohemicus*** | Seidl, 1838 | Ashton Cuckoo Bumble Bee | DD^+^ |
| ***borealis*** | Kirby, 1837 | Northern Amber Bumble Bee | LC |
| ***caliginosus*** | (Frison, 1927) | Obscure Bumble Bee | VU |
| ***centralis*** | Cresson, 1864 | Central Bumble Bee | LC |
| ***citrinus*** | (Smith, 1854) | Lemon Cuckoo Bumble Bee | LC |
| ***cockerelli**** | Franklin, 1913 | Cockerell's Bumble Bee | [CR] |
| ***crotchii*** | Cresson, 1878 | Crotch Bumble Bee | EN |
| ***cryptarum*** | (Fabricius, 1775) | Cryptic Bumble Bee | DD |
| ***distinguendus*** | Morawitz, 1869 | Northern Yellow Bumble Bee | DD |
| ***fervidus*** | (Fabricius, 1798) | Yellow Bumble Bee | VU |
| ***flavidus*** | Eversmann, 1852 | Fernald Cuckoo Bumble Bee | DD |
| ***flavifrons*** | Cresson, 1963 | Yellow Head Bumble Bee | LC |
| ***franklini*** | (Frison, 1921) | Franklin Bumble Bee | CR |
| ***fraternus*** | (Smith, 1854) | Southern Plains Bumble Bee | EN |
| ***frigidus*** | Smith, 1854 | Frigid Bumble Bee | LC |
| ***griseocollis*** | (DeGeer, 1773) | Brown-belted Bumble Bee | LC |
| ***huntii*** | Greene, 1860 | Hunt Bumble Bee | LC |
| ***impatiens*** | Cresson, 1863 | Common Eastern Bumble Bee | LC |
| ***insularis*** | (Smith, 1861) | Indiscriminate Cuckoo Bumble Bee | LC |
| ***jonellus*** | (Kirby, 1802) | White Tail Bumble Bee | DD |
| ***kirbiellus*** | Curtis, 1835 | High Country Bumble Bee (*B. balteatus* Dahlbom, 1832 in Williams et al. 2014) | DD |
| ***kluanensis**** | Williams and Cannings, 2016 | Kluane Bumble Bee | [VU] |
| ***melanopygus*** | Nylander, 1848 | Black Tail Bumble Bee | LC |
| ***mixtus*** | Cresson, 1878 | Fuzzy-horned Bumble Bee | LC |
| ***morrisoni*** | Cresson, 1878 | Morrison Bumble Bee | VU |
| ***natvigi**** | Richards, 1931 | High Arctic Bumble Bee | DD [VU] |
| ***neoboreus*** | Sladen, 1919 | Active Bumble Bee | DD |
| ***nevadensis*** | Cresson, 1874 | Nevada Bumble Bee | LC |
| ***occidentalis*** | Greene, 1858 | Western Bumble Bee | VU |
| ***pensylvanicus*** | (DeGeer, 1773) | American Bumble Bee | VU |
| ***perplexus*** | Cresson, 1863 | Confusing Bumble Bee | LC |
| ***polaris*** | Curtis, 1835 | Polar Bumble Bee | DD |
| ***rufocinctus*** | Cresson, 1863 | Red-belted Bumble Bee | LC |
| ***sandersoni*** | Franklin, 1913 | Sanderson Bumble Bee | LC |
| ***sitkensis*** | Nylander, 1848 | Sitka Bumble Bee | LC |
| ***sp.*** | n/a | Unknown Bumble Bee | n.s. depends on the species |
| ***suckleyi*** | Greene, 1860 | Suckley Cuckoo Bumble Bee | CR |
| ***sylvicola*** | Kirby, 1837 | Forest Bumble Bee | LC |
| ***ternarius*** | Say, 1837 | Tri-colored Bumble Bee | LC |
| ***terricola*** | Kirby, 1837 | Yellow-banded Bumble Bee | VU |
| ***vagans*** | Smith, 1854 | Half-black Bumble Bee | LC |
| ***vagans, perplexus, or sandersoni*** | n/a | Two-striped Yellow Bumble Bee Group | n.s. but species are LC |
| ***vandykei*** | (Frison, 1927) | Van Dyke Bumble Bee | LC |
| ***variabilis*** | Cresson, 1872 | Variable Cuckoo Bumble Bee | CR |
| ***vosnesenskii*** | Radoszkowski, 1862 | Vosnesensky Bumble Bee | LC |
| ***vosnesenskii or caliginosus*** | n/a | Yellow-faced Bumble Bee Group | n.s. but species are LC or VU |
| *Authority, name, and global status from NatureServe [13]  ^+^ Although ranked as DD by the IUCN, *B. bohemicus* has been assessed as Endangered in Canada by COSEWIC [14] and in Ontario by COSSARO [15] | | | |

Note: Names and authorities are from Williams et al. [16] and IUCN North American conservation statuses from IUCN [17] unless otherwise indicated. Abbreviations: DD, Data Deficient; LC, Least Concern; VU, Vulnerable; EN, Endangered; CR, Critically Endangered; [status] global species conservation status from NatureServe [13]; n.s: no status assigned.

**S1 File Table 3. Total number of bumble bee (*Bombus* spp.) records per species from BBNA all years, BBNA 2010-2020, and BBW datasets, and from the BBW + BBNA all years and BBW + 2010-2020 combined data sets.**

| ***Bombus* species** | **BBNA all years** | **BBNA 2010-2020** | **BBW** | **BBNA all years + BBW** | **BBNA 2010-2020 + BBW** |
| --- | --- | --- | --- | --- | --- |
| ***affinis*^1^** | 8,712 | 188 | 725 | 9,437 | 913 |
| ***appositus*** | 4,377 | 404 | 408 | 4,785 | 812 |
| ***auricomus*** | 3,200 | 128 | 269 | 3,469 | 397 |
| ***bifarius*** | 33,727 | 5,736 | 3,460 | 37,187 | 9,196 |
| ***bimaculatus*** | 19,255 | 8,129 | 2,326 | 21,581 | 10,455 |
| ***bohemicus*^2,3^** | 2,797 | 127 | 7 | 2,804 | 134 |
| ***borealis*** | 4,966 | 2,641 | 623 | 5,589 | 3,264 |
| ***caliginosus*^1^** | 2,552 | 89 | 101 | 2,653 | 190 |
| ***centralis*** | 7,685 | 1,202 | 1,009 | 8,694 | 2,211 |
| ***citrinus*** | 3,995 | 575 | 160 | 4,155 | 735 |
| ***cockerelli*^1^** | 120 | 6 | 0 | 120 | 6 |
| ***crotchii*^1^** | 1,837 | 14 | 17 | 1,854 | 31 |
| ***cryptarum*^2^** | 2,462 | 1,087 | 152 | 2,614 | 1,239 |
| ***distinguendus*^2^** | 6 | 0 | 0 | 6 | 0 |
| ***fervidus*^1^** | 20,033 | 1,900 | 1,158 | 21,191 | 3,058 |
| ***flavidus*^2^** | 2,759 | 678 | 244 | 3,003 | 922 |
| ***flavifrons*** | 17,836 | 4,069 | 1,719 | 19,555 | 5,788 |
| ***franklini*^1^** | 324 | 0 | 0 | 324 | 0 |
| ***fraternus*^1^** | 1,846 | 69 | 98 | 1,944 | 167 |
| ***frigidus*** | 8,182 | 2,689 | 30 | 8,212 | 2,719 |
| ***griseocollis*** | 21,604 | 7,039 | 3,474 | 25,078 | 10,513 |
| ***huntii*** | 9,483 | 1,972 | 1,248 | 10,731 | 3,220 |
| ***impatiens*** | 59,991 | 31,694 | 7,755 | 67,746 | 39,449 |
| ***insularis*** | 6,050 | 759 | 505 | 6,555 | 1,264 |
| ***jonellus*^2^** | 5,101 | 2,466 | 1 | 5,102 | 2,467 |
| ***kirbiellus*^2^** | 2,424 | 616 | 12 | 2,436 | 628 |
| ***kluanensis*^1^** | 20 | 14 | 0 | 20 | 14 |
| ***melanopygus*** | 14,725 | 1,665 | 1,326 | 16,051 | 2,991 |
| ***mixtus*** | 18,307 | 8,306 | 1,460 | 19,767 | 9,766 |
| ***morrisoni*^1^** | 4,626 | 73 | 88 | 4,714 | 161 |
| ***natvigi*^1,2^** | 312 | 44 | 0 | 312 | 44 |
| ***neoboreus*^2^** | 521 | 63 | 0 | 521 | 63 |
| ***nevadensis*** | 3,004 | 696 | 805 | 3,809 | 1,501 |
| ***occidentalis*^1^** | 22,572 | 2,934 | 544 | 23,116 | 3,478 |
| ***pensylvanicus*^1^** | 24,509 | 1,460 | 615 | 25,124 | 2,075 |
| ***perplexus*** | 9,552 | 3,439 | 541 | 10,093 | 3,980 |
| ***polaris*^2^** | 1,204 | 242 | 1 | 1,205 | 243 |
| ***rufocinctus*** | 12,521 | 4,297 | 1,733 | 14,254 | 6,030 |
| ***sandersoni*** | 2,158 | 1,096 | 43 | 2,201 | 1,139 |
| ***sitkensis*** | 2,741 | 347 | 221 | 2,962 | 568 |
| ***sp.*** | 0 | 0 | 3,395 | 3,395 | 3,395 |
| ***suckleyi*^1^** | 1,952 | 26 | 2 | 1,954 | 28 |
| ***sylvicola*** | 10,372 | 4,950 | 79 | 10,451 | 5,029 |
| ***ternarius*** | 20,292 | 13,033 | 2,045 | 22,337 | 15,078 |
| ***terricola*^1^** | 19,606 | 1,721 | 625 | 20,231 | 2,346 |
| ***vagans*** | 24,090 | 11,251 | 974 | 25,064 | 12,225 |
| ***vagans, sandersoni* or *perplexus*** | 0 | 0 | 550 | 550 | 550 |
| ***vandykei*** | 963 | 96 | 139 | 1,102 | 235 |
| ***variabilis*^1^** | 956 | 0 | 0 | 956 | 0 |
| ***vosnesenskii*** | 28,832 | 1,971 | 2,608 | 31,440 | 4,579 |
| ***vosnesenskii or caliginosus*^1^** | 0 | 0 | 839 | 839 | 839 |
|  |  |  |  |  |  |
| total # records | 475,159 | 132,001 | 44,134 | 519,293 | 176,135 |
| ^1^ species considered to be at-risk of extinction, with an IUCN or NatureServe status of VU, EN, CR  ^2^ species that are considered to be data deficient (DD) and thus no determination of their at-risk status can be made (i.e. may be LC, VU, EN, or CR)  ^3^ species considered to be at-risk of extinction by a federal assessment organization despite overall IUCN rank of DD | | | | | |

**S1 File Table 4. The number of total and unique bumble bee species (including three species groups: "sp", "*vagans, sandersoni* or *perplexus*", and "*vosnesenskii* or *caliginosus*") per province, state, and territory, by dataset.**

| **Province, State, Territory** | **# species in BBNA all years** | **# species in BBNA 2010-2020** | **# species in BBW** | **# species in BBW and not in BBNA all years** | **# species in BBW and not in BBNA 2010-2020?** | **# species found in this region** |
| --- | --- | --- | --- | --- | --- | --- |
| **Alabama** | 9 | 0 | 6 | 1 | 6 | 10 |
| **Alaska** | 27 | 24 | 9 | 1 | 2 | 28 |
| **Alberta** | 29 | 26 | 27 | 3 | 15 | 32 |
| **Arizona** | 17 | 14 | 6 | 1 | 5 | 18 |
| **Arkansas** | 8 | 4 | 6 | 1 | 5 | 9 |
| **British Columbia** | 34 | 26 | 26 | 2 | 12 | 36 |
| **California** | 25 | 21 | 20 | 2 | 8 | 27 |
| **Colorado** | 26 | 21 | 20 | 2 | 17 | 28 |
| **Connecticut** | 16 | 10 | 4 | 1 | 2 | 17 |
| **Delaware** | 10 | 6 | 4 | 1 | 1 | 11 |
| **District of Columbia** | 10 | 7 | 3 | 0 | 1 | 10 |
| **Florida** | 6 | 4 | 6 | 1 | 2 | 7 |
| **Georgia** | 15 | 6 | 6 | 1 | 5 | 16 |
| **Idaho** | 22 | 16 | 20 | 1 | 16 | 23 |
| **Illinois** | 17 | 10 | 11 | 1 | 3 | 18 |
| **Indiana** | 14 | 9 | 10 | 2 | 8 | 16 |
| **Iowa** | 14 | 3 | 12 | 2 | 9 | 16 |
| **Kansas** | 12 | 0 | 8 | 1 | 4 | 13 |
| **Kentucky** | 10 | 3 | 6 | 1 | 6 | 11 |
| **Louisiana** | 6 | 0 | 4 | 1 | 1 | 7 |
| **Maine** | 18 | 12 | 15 | 3 | 4 | 21 |
| **Manitoba** | 27 | 8 | 15 | 3 | 6 | 30 |
| **Maryland** | 14 | 11 | 10 | 2 | 5 | 16 |
| **Massachusetts** | 18 | 13 | 11 | 2 | 9 | 20 |
| **Michigan** | 21 | 15 | 14 | 2 | 4 | 23 |
| **Minnesota** | 20 | 11 | 20 | 3 | 13 | 23 |
| **Mississippi** | 8 | 0 | 5 | 1 | 5 | 9 |
| **Missouri** | 10 | 4 | 9 | 2 | 5 | 12 |
| **Montana** | 28 | 27 | 16 | 1 | 12 | 29 |
| **Nebraska** | 17 | 8 | 11 | 1 | 4 | 18 |
| **Nevada** | 18 | 8 | 5 | 0 | 1 | 18 |
| **New Brunswick** | 18 | 15 | 14 | 2 | 2 | 20 |
| **New Hampshire** | 18 | 13 | 7 | 2 | 5 | 20 |
| **New Jersey** | 17 | 9 | 6 | 2 | 2 | 19 |
| **New Mexico** | 18 | 6 | 5 | 2 | 5 | 20 |
| **New York** | 19 | 15 | 13 | 2 | 11 | 21 |
| **Newfoundland and Labrador** | 19 | 12 | 14 | 2 | 5 | 21 |
| **North Carolina** | 15 | 11 | 10 | 2 | 10 | 17 |
| **North Dakota** | 24 | 1 | 11 | 1 | 7 | 25 |
| **Northwest Territories** | 21 | 16 | 6 | 1 | 6 | 22 |
| **Nova Scotia** | 16 | 13 | 12 | 2 | 2 | 18 |
| **Nunavut** | 10 | 5 | 2 | 1 | 2 | 11 |
| **Ohio** | 20 | 8 | 12 | 2 | 3 | 22 |
| **Oklahoma** | 9 | 4 | 5 | 1 | 4 | 10 |
| **Ontario** | 27 | 16 | 23 | 2 | 6 | 29 |
| **Oregon** | 25 | 16 | 24 | 2 | 21 | 27 |
| **Pennsylvania** | 16 | 10 | 10 | 2 | 4 | 18 |
| **Prince Edward Island** | 14 | 10 | 14 | 2 | 12 | 16 |
| **Quebec** | 25 | 17 | 20 | 2 | 14 | 27 |
| **Rhode Island** | 10 | 7 | 7 | 3 | 3 | 13 |
| **Saskatchewan** | 24 | 18 | 20 | 4 | 18 | 28 |
| **South Carolina** | 12 | 3 | 6 | 1 | 6 | 13 |
| **South Dakota** | 28 | 13 | 15 | 2 | 8 | 30 |
| **Tennessee** | 16 | 8 | 7 | 1 | 1 | 17 |
| **Texas** | 12 | 4 | 7 | 1 | 5 | 13 |
| **Utah** | 23 | 13 | 13 | 1 | 10 | 24 |
| **Vermont** | 17 | 13 | 12 | 2 | 7 | 19 |
| **Virginia** | 17 | 14 | 13 | 2 | 12 | 19 |
| **Washington** | 26 | 21 | 25 | 3 | 13 | 29 |
| **West Virginia** | 14 | 12 | 8 | 2 | 8 | 16 |
| **Wisconsin** | 20 | 7 | 18 | 3 | 2 | 23 |
| **Wyoming** | 23 | 18 | 15 | 1 | 15 | 24 |
| **Yukon** | 26 | 20 | 3 | 0 | 3 | 26 |
| **Total # species** | **48** | **45** | **44** | **3** | **3** | **51** |

**S1 File Table 5. The total number of bumble bee records (all species combined) per province, state, and territory by dataset.**

| **Province, State, Territory** | **# BBNA records all years** | **# BBNA records 2010-2020** | **#BBW records** | **total # records (BBNA all years & BBW)** |
| --- | --- | --- | --- | --- |
| **Alabama** | 188 | 0 | 60 | 248 |
| **Alaska** | 40,468 | 20,310 | 48 | 40,516 |
| **Alberta** | 16,251 | 10,727 | 1,821 | 18,072 |
| **Arizona** | 6,431 | 388 | 10 | 6,441 |
| **Arkansas** | 204 | 19 | 41 | 245 |
| **British Columbia** | 12,522 | 1,410 | 1,257 | 13,779 |
| **California** | 61,101 | 2,200 | 481 | 61,582 |
| **Colorado** | 26,259 | 779 | 217 | 26,476 |
| **Connecticut** | 6,639 | 1,281 | 26 | 6,665 |
| **Delaware** | 382 | 48 | 21 | 403 |
| **District of Columbia** | 640 | 369 | 9 | 649 |
| **Florida** | 2,239 | 15 | 48 | 2,287 |
| **Georgia** | 1,339 | 68 | 47 | 1,386 |
| **Idaho** | 4,601 | 316 | 4,644 | 9,245 |
| **Illinois** | 10,176 | 392 | 212 | 10,388 |
| **Indiana** | 4,028 | 1,035 | 112 | 4,140 |
| **Iowa** | 1,367 | 5 | 198 | 1,565 |
| **Kansas** | 1,731 | 0 | 47 | 1,778 |
| **Kentucky** | 724 | 24 | 33 | 757 |
| **Louisiana** | 123 | 0 | 15 | 138 |
| **Maine** | 2,490 | 1,258 | 324 | 2,814 |
| **Manitoba** | 2,475 | 46 | 352 | 2,827 |
| **Maryland** | 4,738 | 3,066 | 85 | 4,823 |
| **Massachusetts** | 14,758 | 8,001 | 156 | 14,914 |
| **Michigan** | 9,512 | 669 | 400 | 9,912 |
| **Minnesota** | 12,899 | 584 | 2,662 | 15,561 |
| **Mississippi** | 1,871 | 0 | 11 | 1,882 |
| **Missouri** | 3,037 | 8 | 128 | 3,165 |
| **Montana** | 14,554 | 5,567 | 191 | 14,745 |
| **Nebraska** | 3,166 | 25 | 1,555 | 4,721 |
| **Nevada** | 2,926 | 43 | 15 | 2,941 |
| **New Brunswick** | 1,424 | 923 | 575 | 1,999 |
| **New Hampshire** | 6,842 | 1,400 | 54 | 6,896 |
| **New Jersey** | 10,464 | 5,057 | 46 | 10,510 |
| **New Mexico** | 3,484 | 25 | 18 | 3,502 |
| **New York** | 11,142 | 854 | 245 | 11,387 |
| **Newfoundland and Labrador** | 2,857 | 2,205 | 240 | 3,097 |
| **North Carolina** | 4,855 | 1,578 | 80 | 4,935 |
| **North Dakota** | 3,415 | 1 | 49 | 3,464 |
| **Northwest Territories** | 993 | 110 | 12 | 1,005 |
| **Nova Scotia** | 3,115 | 634 | 581 | 3,696 |
| **Nunavut** | 1,089 | 66 | 7 | 1,096 |
| **Ohio** | 4,699 | 95 | 274 | 4,973 |
| **Oklahoma** | 1,030 | 224 | 60 | 1,090 |
| **Ontario** | 36,121 | 19,322 | 9,581 | 45,702 |
| **Oregon** | 19,637 | 6,773 | 6,292 | 25,929 |
| **Pennsylvania** | 6,149 | 2,910 | 159 | 6,308 |
| **Prince Edward Island** | 1,519 | 149 | 187 | 1,706 |
| **Quebec** | 5,043 | 2,899 | 1,168 | 6,211 |
| **Rhode Island** | 377 | 161 | 45 | 422 |
| **Saskatchewan** | 1,598 | 557 | 314 | 1,912 |
| **South Carolina** | 532 | 4 | 27 | 559 |
| **South Dakota** | 3,475 | 675 | 138 | 3,613 |
| **Tennessee** | 800 | 293 | 42 | 842 |
| **Texas** | 4,060 | 827 | 223 | 4,283 |
| **Utah** | 10,110 | 374 | 52 | 10,162 |
| **Vermont** | 15,503 | 12,123 | 135 | 15,638 |
| **Virginia** | 4,997 | 3,675 | 151 | 5,148 |
| **Washington** | 13,473 | 1,588 | 7,143 | 20,616 |
| **West Virginia** | 5,375 | 3,824 | 33 | 5,408 |
| **Wisconsin** | 4,560 | 82 | 895 | 5,455 |
| **Wyoming** | 8,214 | 1,419 | 79 | 8,293 |
| **Yukon** | 8,368 | 2,521 | 3 | 8,371 |
| **Total # records** | **475,159** | **132,001** | **44,134** | **519,293** |

**S1 File Table 6. A comparison of the mean numbers of bee records, species richness, Menhinick’s Index, and Shannon-Weiner species diversity index values in BBW as compared to BBNA all years and BBNA 2010-2010 at three scales: province (including states and territories)(n=63), 100km x 100km grids (n=1485), and 10km x 10km grids (n=19314).**

| **Dataset comparison** | **Variable** | **Scale** | **Dataset** | **Mean** | **SE** | **Statistical test results** |
| --- | --- | --- | --- | --- | --- | --- |
| BBW vs BBNA all years | # bee records | Province | BBNA_allyears  BBW | 86.241  127.732 | 26.605  21.474 | U = 1,047.000, p-value ≤ 0.001* |
| BBW vs BBNA 2010-2020 | # bee records | Province | BBNA 2010-2020  BBW | 158.32  127.732 | 32.637  21.474 | U = 1,576.500, p-value = 0.047* |
| BBW vs BBNA all years | species richness | Province | BBNA_allyears  BBW | 17.857  11.460 | 0.814  0.795 | U = 3,031.500, p-value ≤ 0.001* |
| BBW vs BBNA 2010-2020 | species richness | Province | BBNA 2010-2020  BBW | 11.032  11.460 | 0.85  0.795 | U = 1,946.000, p-value = 0.853 |
| BBW vs BBNA all years | Menhinick's Index | Province | BBNA_allyears  BBW | 0.306  0.95 | 0.018  0.048 | U = 199.000, p-value ≤ 0.001* |
| BBW vs BBNA 2010-2020 | Menhinick's Index | Province | BBNA 2010-2020  BBW | 0.299  0.95 | 0.028  0.048 | U = 242.500, p-value ≤ 0.001* |
| BBW vs BBNA all years | species diversity | Province | BBNA_allyears  BBW | 1.959  1.789 | 0.062  0.061 | U = 2,401.000, p-value = 0.177 |
| BBW vs BBNA 2010-2020 | species diversity | Province | BBNA 2010-2020  BBW | 1.434  1.789 | 0.089  0.061 | T = -3.296, p-value = 0.001* |
| BBW vs BBNA all years | # bee records | 100km x 100km | BBNA_allyears  BBW | 319.972  29.72 | 22.51  3.338 | U = 1,835,000.000, p-value ≤ 0.001* |
| BBW vs BBNA 2010-2020 | # bee records | 100km x 100km | BBNA 2010-2020  BBW | 88.89  29.72 | 10.918  3.338 | U = 1,020,000.000, p-value ≤ 0.001* |
| BBW vs BBNA all years | species richness | 100km x 100km | BBNA_allyears  BBW | 7.253  2.923 | 0.139  0.109 | U = 1,710,000.000, p-value ≤ 0.001* |
| BBW vs BBNA 2010-2020 | species richness | 100km x 100km | BBNA_2010_2020  BBW | 2.255  2.923 | 0.098  0.109 | U = 951,213.000, p-value ≤ 0.001* |
| BBW vs BBNA all years | Menhinick's Index | 100km x 100km | BBNA_allyears  BBW | 0.835  0.04 | 0.011  0.005 | U = 2,114,000.000, p-value ≤ 0.001* |
| BBW vs BBNA 2010-2020 | Menhinick's Index | 100km x 100km | BBNA_2010_2020  BBW | 0.024  0.04 | 0.004  0.005 | U = 1,099,000.000, p-value = 0.625 |
| BBW vs BBNA all years | species diversity | 100km x 100km | BBNA_allyears  BBW | 1.209  0.601 | 0.019  0.02 | U = 1,582,000.000, p-value ≤ 0.001 |
| BBW vs BBNA 2010-2020 | species diversity | 100km x 100km | BBNA_2010_2020  BBW | 0.061  0.601 | 0.008  0.02 | U = 678,257.500, p-value ≤ 0.001* |
| BBW vs BBNA all years | # bee records | 10km x 10km | BBNA_allyears  BBW | 2.36  0.219 | 0.094  0.011 | U = 21,260,000,000.000, p-value ≤ 0.001* |
| BBW vs BBNA 2010-2020 | # bee records | 10km x 10km | BBNA_2010-2020  BBW | 0.655  0.219 | 0.05  0.011 | U = 20,010,000,000.000, p-value ≤ 0.001* |
| BBW vs BBNA all years | species richness | 10km x 10km | BBNA_allyears  BBW | 2.444  0.766 | 0.019  0.012 | U = 285,700,000.000, p-value ≤ 0.001* |
| BBW vs BBNA 2010-2020 | species richness | 10km x 10km | BBNA_2010-2020  BBW | 0.56  0.766 | 0.011  0.012 | U = 162,900,000.000, p-value ≤ 0.001* |
| BBW vs BBNA all years | Menhinick's Index | 10km x 10km | BBNA_allyears  BBW | 1.302  0.376 | 0.006  0.004 | U = 296,500,000.000, p-value ≤ 0.001* |
| BBW vs BBNA 2010-2020 | Menhinick's Index | 10km x 10km | BBNA_2010-2020  BBW | 0.295  0.376 | 0.005  0.004 | U = 165,000,000.000, p-value ≤ 0.001* |
| BBW vs BBNA all years | species diversity | 10km x 10km | BBNA_allyears  BBW | 0.517  0.171 | 0.004  0.003 | U = 247,300,000.000, p-value ≤ 0.001* |
| BBW vs BBNA 2010-2020 | species diversity | 10km x 10km | BBNA_2010-2020  BBW | 0.119  0.171 | 0.003  0.003 | U = 178,300,000.000, p-value ≤ 0.001* |

The means were based on the individual calculation for all species within each scale variable (e.g. for each province or state) and dataset using only provinces or grids where there was at least one record amongst all databases. Values presented here are mean ± standard error (SE); see **S1 File Tables 6-7, S2 File Table 1** for the individual values for each scale variable. Average Species Richness is calculated as the total number of species, Average Menhinick’s Index is the total number species divided by the square-root of the total number of individuals, and the Average Shannon-Weiner Index (species diversity) is calculated as -1*sum(pi*lnpi), where pi is the proportion of each species. Statistical tests conducted were either Independent samples T-tests (T) or Mann-Whitney U-tests (U). * indicates significance at p=0.05.

**S1 File Table7. The total Menhinick’s and Shannon-Weiner diversity indices (all species combined) per province, state, and territory, by dataset**.

|  | **Menhinick's index** | | | **Shannon-Weiner diversity index** | | |
| --- | --- | --- | --- | --- | --- | --- |
| **Province, State, Territory** | **BBNA all years** | **BBNA 2010-2020** | **BBW** | **BBNA all years** | **BBNA 2010-2020** | **BBW** |
| **Alabama** | 0.66 | n/a | 0.77 | 1.33 | n/a | 1.23 |
| **Alaska** | 0.13 | 0.17 | 1.30 | 2.47 | 2.36 | 1.86 |
| **Alberta** | 0.23 | 0.25 | 0.63 | 2.63 | 2.13 | 2.71 |
| **Arizona** | 0.21 | 0.71 | 1.90 | 1.43 | 1.96 | 1.61 |
| **Arkansas** | 0.56 | 0.92 | 0.94 | 1.40 | 1.01 | 1.50 |
| **British Columbia** | 0.30 | 0.69 | 0.73 | 2.45 | 2.43 | 2.61 |
| **California** | 0.10 | 0.45 | 0.91 | 2.21 | 1.89 | 2.07 |
| **Colorado** | 0.16 | 0.75 | 1.36 | 2.77 | 2.56 | 2.26 |
| **Connecticut** | 0.20 | 0.28 | 0.78 | 1.95 | 1.05 | 0.94 |
| **Delaware** | 0.51 | 0.87 | 0.87 | 1.55 | 1.12 | 0.96 |
| **District of Columbia** | 0.40 | 0.36 | 1.00 | 1.47 | 0.79 | 1.06 |
| **Florida** | 0.13 | 1.03 | 0.87 | 1.27 | 1.06 | 1.36 |
| **Georgia** | 0.41 | 0.73 | 0.88 | 1.61 | 1.08 | 1.41 |
| **Idaho** | 0.32 | 0.90 | 0.29 | 2.46 | 1.58 | 2.58 |
| **Illinois** | 0.17 | 0.51 | 0.76 | 1.97 | 1.80 | 1.75 |
| **Indiana** | 0.22 | 0.28 | 0.94 | 1.92 | 1.60 | 1.80 |
| **Iowa** | 0.38 | 1.34 | 0.85 | 1.66 | 1.05 | 1.81 |
| **Kansas** | 0.29 | n/a | 1.17 | 1.68 | n/a | 1.84 |
| **Kentucky** | 0.37 | 0.61 | 1.04 | 0.95 | 0.46 | 1.57 |
| **Louisiana** | 0.54 | n/a | 1.03 | 1.33 | n/a | 1.18 |
| **Maine** | 0.36 | 0.34 | 0.83 | 2.09 | 1.53 | 2.23 |
| **Manitoba** | 0.54 | 1.18 | 0.80 | 2.50 | 1.68 | 2.10 |
| **Maryland** | 0.20 | 0.20 | 1.08 | 1.64 | 1.34 | 1.78 |
| **Massachusetts** | 0.15 | 0.15 | 0.88 | 1.84 | 1.14 | 1.35 |
| **Michigan** | 0.22 | 0.58 | 0.70 | 2.38 | 2.10 | 1.74 |
| **Minnesota** | 0.18 | 0.46 | 0.39 | 2.48 | 1.48 | 2.27 |
| **Mississippi** | 0.18 | n/a | 1.51 | 1.39 | n/a | 1.52 |
| **Missouri** | 0.18 | 1.41 | 0.80 | 1.70 | 1.26 | 1.95 |
| **Montana** | 0.23 | 0.36 | 1.16 | 2.65 | 2.63 | 2.29 |
| **Nebraska** | 0.30 | 1.60 | 0.28 | 1.90 | 1.71 | 1.53 |
| **Nevada** | 0.33 | 1.22 | 1.29 | 2.32 | 1.70 | 1.43 |
| **New Brunswick** | 0.48 | 0.49 | 0.58 | 2.23 | 2.28 | 1.99 |
| **New Hampshire** | 0.22 | 0.35 | 0.95 | 2.09 | 1.53 | 1.38 |
| **New Jersey** | 0.17 | 0.13 | 0.88 | 1.48 | 1.12 | 1.43 |
| **New Mexico** | 0.30 | 1.20 | 1.18 | 2.31 | 1.46 | 1.35 |
| **New York** | 0.18 | 0.51 | 0.83 | 2.40 | 2.08 | 1.89 |
| **Newfoundland and Labrador** | 0.36 | 0.26 | 0.90 | 1.50 | 0.96 | 2.21 |
| **North Carolina** | 0.22 | 0.28 | 1.12 | 2.08 | 1.40 | 1.89 |
| **North Dakota** | 0.41 | 1.00 | 1.57 | 2.00 | 0.00 | 2.14 |
| **Northwest Territories** | 0.67 | 1.53 | 1.73 | 2.38 | 2.51 | 1.54 |
| **Nova Scotia** | 0.29 | 0.52 | 0.50 | 2.30 | 2.32 | 2.03 |
| **Nunavut** | 0.30 | 0.62 | 0.76 | 1.23 | 1.18 | 0.41 |
| **Ohio** | 0.29 | 0.82 | 0.72 | 2.23 | 0.56 | 1.60 |
| **Oklahoma** | 0.28 | 0.27 | 0.65 | 1.12 | 0.76 | 1.43 |
| **Ontario** | 0.14 | 0.12 | 0.23 | 2.37 | 2.04 | 1.98 |
| **Oregon** | 0.18 | 0.19 | 0.30 | 2.35 | 1.34 | 2.54 |
| **Pennsylvania** | 0.20 | 0.19 | 0.79 | 1.54 | 0.78 | 1.75 |
| **Prince Edward Island** | 0.36 | 0.82 | 1.02 | 2.03 | 1.65 | 1.95 |
| **Quebec** | 0.35 | 0.32 | 0.59 | 2.57 | 2.13 | 2.27 |
| **Rhode Island** | 0.52 | 0.55 | 1.04 | 1.98 | 1.48 | 1.51 |
| **Saskatchewan** | 0.60 | 0.76 | 1.13 | 2.66 | 2.35 | 2.40 |
| **South Carolina** | 0.52 | 1.50 | 1.15 | 1.47 | 1.04 | 1.61 |
| **South Dakota** | 0.47 | 0.50 | 1.28 | 2.47 | 1.58 | 2.23 |
| **Tennessee** | 0.57 | 0.47 | 1.08 | 2.07 | 1.08 | 1.57 |
| **Texas** | 0.19 | 0.14 | 0.47 | 0.57 | 0.45 | 1.05 |
| **Utah** | 0.23 | 0.67 | 1.80 | 2.34 | 1.76 | 2.32 |
| **Vermont** | 0.14 | 0.12 | 1.03 | 2.01 | 1.75 | 1.86 |
| **Virginia** | 0.24 | 0.23 | 1.06 | 1.74 | 1.47 | 1.93 |
| **Washington** | 0.22 | 0.53 | 0.30 | 2.66 | 2.46 | 2.76 |
| **West Virginia** | 0.19 | 0.19 | 1.39 | 1.32 | 1.05 | 1.62 |
| **Wisconsin** | 0.30 | 0.77 | 0.60 | 1.74 | 0.54 | 2.30 |
| **Wyoming** | 0.25 | 0.48 | 1.69 | 2.31 | 2.35 | 2.36 |
| **Yukon** | 0.28 | 0.40 | 1.73 | 2.44 | 2.32 | 1.10 |
| **All data combined** | **0.07** | **0.12** | **0.21** | **3.31** | **2.87** | **3.07** |

Menhinick's index is the number of species divided by the square-root of the total number of individuals. Shannon-Weiner diversity index is pi * lnpi where pi = relative individual species density.

**S1 File Table 8. A comparison of the number of BBW records that fall outside of the Extent of Occurrence (EOO) for each bumble bee (*Bombus*) species in the BBNA all years and BBNA 2010-2020 datasets.**

| **Species** | **BBW records outside BBNA EOO** | |
| --- | --- | --- |
|  | **BBW vs BBNA all years** | **BBW vs BBNA 2010-2020** |
| ***affinis*** | 1 | 126 |
| ***appositus*** | 0 | 27 |
| ***auricomus*** | 1 | 13 |
| ***bifarius*** | 0 | 3 |
| ***bimaculatus*** | 88 | 262 |
| ***bohemicus*** | 0 | 2 |
| ***borealis*** | 10 | 43 |
| ***caliginosus*** | 1 | 87 |
| ***centralis*** | 0 | 2 |
| ***citrinus*** | 4 | 4 |
| ***cockerelli*** | n/a | n/a |
| ***crotchii*** | 0 | 15 |
| ***cryptarum*** | 17 | 23 |
| ***distinguendus*** | n/a | n/a |
| ***fervidus*** | 0 | 2 |
| ***flavidus*** | 0 | 1 |
| ***flavifrons*** | 0 | 3 |
| ***franklini*** | n/a | n/a |
| ***fraternus*** | 0 | 16 |
| ***frigidus*** | 1 | 3 |
| ***griseocollis*** | 14 | 128 |
| ***huntii*** | 2 | 51 |
| ***impatiens*** | 121 | 219 |
| ***insularis*** | 0 | 0 |
| ***jonellus*** | 0 | 0 |
| ***kirbiellus*** | 0 | 4 |
| ***kluanensis*** | n/a | n/a |
| ***melanopygus*** | 2 | 30 |
| ***mixtus*** | 0 | 3 |
| ***morrisoni*** | 1 | 53 |
| ***natvigi*** | n/a | n/a |
| ***neoboreus*** | n/a | n/a |
| ***nevadensis*** | 8 | 46 |
| ***occidentalis*** | 1 | 7 |
| ***pensylvanicus*** | 0 | 13 |
| ***perplexus*** | 1 | 9 |
| ***polaris*** | 0 | 1 |
| ***rufocinctus*** | 27 | 54 |
| ***sandersoni*** | 3 | 21 |
| ***sitkensis*** | 0 | 5 |
| ***sp.*** | n/a | n/a |
| ***suckleyi*** | 0 | 1 |
| ***sylvicola*** | 4 | 4 |
| ***ternarius*** | 11 | 39 |
| ***terricola*** | 20 | 34 |
| ***vagans*** | 5 | 156 |
| ***vagans, sandersoni* or *perplexus*** | n/a | n/a |
| ***vandykei*** | 0 | 17 |
| ***variabilis*** | n/a | n/a |
| ***vosnesenskii*** | 44 | 456 |
| ***vosnesenskii* or *caliginosus*** | n/a | n/a |
| **all species combined** | 21 | 131 |

**S1 File Table 9. A comparison of the Extent of Occurrence (EOO) geographic range area and difference in EOO areas per species between BBW dataset and the BBNA all years or BBNA 2010-2020 datasets.**

|  | **EOO area (km^2^)** | | | **Difference in EOO area (km^2^) (BBW less BBNA)** | |
| --- | --- | --- | --- | --- | --- |
| **Species** | **BBNA all years** | **BBNA 2010-2020** | **BBW** | **BBW vs BBNA all years** | **BBW vs BBNA 2010-2020** |
| ***affinis*** | 2,695,720 | 447,577 | 1,284,220 | -1,411,500 | 836,643 |
| ***appositus*** | 3,099,020 | 1,824,600 | 1,172,080 | -1,926,940 | -652,520 |
| ***auricomus*** | 4,240,090 | 2,591,170 | 2,433,400 | -1,806,690 | -157,770 |
| ***bifarius*** | 9,424,620 | 6,278,810 | 2,834,600 | -6,590,020 | -3,444,210 |
| ***bimaculatus*** | 5,787,300 | 3,834,660 | 5,336,340 | -450,960 | 1,501,680 |
| ***bohemicus*** | 13,678,200 | 5,195,400 | 2,792,900 | -10,885,300 | -2,402,500 |
| ***borealis*** | 12,107,600 | 8,991,360 | 5,169,280 | -6,938,320 | -3,822,080 |
| ***caliginosus*** | 551,953 | 96,711 | 322,515 | -229,438 | 225,804 |
| ***centralis*** | 8,611,140 | 4,958,690 | 2,518,260 | -6,092,880 | -2,440,430 |
| ***citrinus*** | 5,510,460 | 5,074,500 | 3,127,740 | -2,382,720 | -1,946,760 |
| ***cockerelli*** | 1,297 | 0 | no records | n/a | n/a |
| ***crotchii*** | 357,698 | 2,433 | 84,010 | -273,688 | 81,577 |
| ***cryptarum*** | 6,548,630 | 2,256,180 | 4,962,840 | -1,585,790 | 2,706,660 |
| ***distinguendus*** | 409,904 | no records | no records | n/a | n/a |
| ***fervidus*** | 17,952,500 | 13,776,700 | 8,705,000 | -9,247,500 | -5,071,700 |
| ***flavidus*** | 18,436,000 | 15,396,100 | 7,187,510 | -11,248,490 | -8,208,590 |
| ***flavifrons*** | 15,877,600 | 6,706,140 | 3,734,950 | -12,142,650 | -2,971,190 |
| ***franklini*** | 19,589 | no records | no records | n/a | n/a |
| ***fraternus*** | 4,545,830 | 1,937,690 | 2,141,730 | -2,404,100 | 204,040 |
| ***frigidus*** | 15,756,600 | 12,405,800 | 5,614,210 | -10,142,390 | -6,791,590 |
| ***griseocollis*** | 9,462,960 | 6,635,110 | 7,403,930 | -2,059,030 | 768,820 |
| ***huntii*** | 5,446,530 | 2,807,540 | 3,412,950 | -2,033,580 | 605,410 |
| ***impatiens*** | 9,944,970 | 5,983,250 | 9,304,660 | -640,310 | 3,321,410 |
| ***insularis*** | 15,523,800 | 13,186,400 | 6,698,170 | -8,825,630 | -6,488,230 |
| ***jonellus*** | 4,145,690 | 2,043,470 | 0 | -4,145,690 | -2,043,470 |
| ***kirbiellus*** | 16,069,500 | 6,066,480 | 1,884,860 | -14,184,640 | -4,181,620 |
| ***kluanensis*** | 11,114 | 8,415 | no records | n/a | n/a |
| ***melanopygus*** | 18,102,600 | 11,398,200 | 11,077,400 | -7,025,200 | -320,800 |
| ***mixtus*** | 16,760,700 | 5,564,230 | 2,863,910 | -13,896,790 | -2,700,320 |
| ***morrisoni*** | 2,999,980 | 1,008,850 | 1,304,430 | -1,695,550 | 295,580 |
| ***natvigi*** | 7,790,570 | 4,132,230 | no records | n/a | n/a |
| ***neoboreus*** | 4,062,620 | 905,351 | no records | n/a | n/a |
| ***nevadensis*** | 6,337,480 | 4,118,470 | 4,662,030 | -1,675,450 | 543,560 |
| ***occidentalis*** | 10,346,600 | 5,282,160 | 5,895,740 | -4,450,860 | 613,580 |
| ***pensylvanicus*** | 9,180,680 | 6,813,980 | 5,762,510 | -3,418,170 | -1,051,470 |
| ***perplexus*** | 10,668,500 | 8,795,900 | 3,994,910 | -6,673,590 | -4,800,990 |
| ***polaris*** | 13,735,400 | 8,260,170 | 0 | -13,735,400 | -8,260,170 |
| ***rufocinctus*** | 14,327,000 | 11,602,000 | 7,611,200 | -6,715,800 | -3,990,800 |
| ***sandersoni*** | 8,832,360 | 7,416,070 | 3,581,700 | -5,250,660 | -3,834,370 |
| ***sitkensis*** | 5,680,490 | 2,236,540 | 1,182,750 | -4,497,740 | -1,053,790 |
| ***sp.*** | no records | no records | 22,812,300 | n/a | n/a |
| ***suckleyi*** | 13,958,500 | 8,292,670 | 3 | -13,958,497 | -8,292,667 |
| ***sylvicola*** | 23,973,300 | 23,973,300 | 12,311,800 | -11,661,500 | -11,661,500 |
| ***ternarius*** | 10,445,900 | 6,800,730 | 7,123,760 | -3,322,140 | 323,030 |
| ***terricola*** | 13,094,000 | 7,910,660 | 6,933,690 | -6,160,310 | -976,970 |
| ***vagans*** | 14,299,700 | 7,175,190 | 7,991,690 | -6,308,010 | 816,500 |
| ***vagans, sandersoni* or *perplexus*** | no records | no records | 6,055,720 | n/a | n/a |
| ***vandykei*** | 748,521 | 505,566 | 240,012 | -508,509 | -265,554 |
| ***variabilis*** | 5,944,340 | no records | no records | n/a | n/a |
| ***vosnesenskii*** | 1,428,890 | 557,806 | 1,014,040 | -414,850 | 456,234 |
| ***vosnesenskii* or *caliginosus*** | no records | no records | 831,586 | n/a | n/a |
| **all species combined** | 31,862,000 | 26,323,600 | 25,840,000 | -6,022,000 | -483,600 |
| **Mean ± standard error (se)** | 9,773,049 ± 912,234.6 | 6,249,006 ± 763,651.8 | 4,187,261 ± 497,999.5 | -5,585,787 ± 691,597.5 | -2,061,744 ± 518,783.7 |
| **Mann-Whitney U-test (U)**  **on EOOs** | BBNA all years vs BBW  U=1322, p<0.001* | BBNA 2010-2020 vs BBW  U=1048, p=0.055 |  | (BBW-BBNA all years) vs (BBW-BBNA 2010-2020)  U=426.5, p<0.001* | |

See the text for discussion about constraints with interpreting these values as areas of usable range (e.g. the inclusion of areas in the EOO that are not suitable habitat).

**S1 File Table 10. A comparison of the number of 100km x 100km grids: with records per species per dataset; where BBW confirms the presence of a species in BBNA all years (i.e. in both BBNA and BBW); where BBW shows novel recent persistence (i.e. present historically in BBNA all years but only found in the last decade through BBW); with unique grids over the combined all years and 2010-2020 datasets due to the BBW dataset; and the total number of girds in the combined BBNA all years + BBW and BBNA 2010-2020 + BBW datasets.** This latter measure can also be used to calculate the total range size by multiplying the number of grids per combined dataset by 100km x 100km.

| **Comparison of 100km x 100km grids being tested per *Bombus* species** | **total # 100km x 100km grids per dataset** | | | **# grids where BBW confirms presence*** | **# grids where BBW shows novel recent persistence*** | **# unique grids due to BBW over all years** | **# unique grids due to BBW over 2010-2020** | **# grids with any records in combined all years data** | **# grids with any records in combined 2010-2020 data** |
| --- | --- | --- | --- | --- | --- | --- | --- | --- | --- |
| **Datasets being compared** | BBNA all years | BBNA 2010-2020 | BBW | BBW in same grid as BBNA all years | BBW in same grid as BBNA all years, when no BBNA 2010-2020 present | BBW vs BBNA all years | BBW vs BBNA 2010-2020 | BBW and BBNA all years | BBW and BBNA 2010-2020 |
| ***affinis*** | 178 | 23 | 30 | 23 | 7 | 7 | 14 | 185 | 37 |
| ***appositus*** | 173 | 46 | 67 | 59 | 41 | 8 | 49 | 181 | 95 |
| ***auricomus*** | 221 | 31 | 63 | 52 | 43 | 11 | 54 | 232 | 85 |
| ***bifarius*** | 292 | 99 | 111 | 99 | 50 | 12 | 62 | 304 | 161 |
| ***bimaculatus*** | 325 | 130 | 217 | 163 | 78 | 54 | 132 | 379 | 262 |
| ***bohemicus*** | 263 | 22 | 5 | 2 | 1 | 3 | 4 | 266 | 26 |
| ***borealis*** | 277 | 104 | 151 | 126 | 72 | 25 | 97 | 302 | 201 |
| ***caliginosus*** | 44 | 7 | 20 | 18 | 15 | 2 | 17 | 46 | 24 |
| ***centralis*** | 243 | 78 | 90 | 71 | 47 | 19 | 66 | 262 | 144 |
| ***citrinus*** | 189 | 64 | 46 | 29 | 15 | 17 | 32 | 206 | 96 |
| ***cockerelli*** | 2 | 1 | 0 | 0 | 0 | 0 | 0 | 2 | 1 |
| ***crotchii*** | 35 | 3 | 9 | 9 | 6 | 0 | 6 | 35 | 9 |
| ***cryptarum*** | 104 | 57 | 28 | 16 | 4 | 12 | 16 | 116 | 73 |
| ***distinguendus*** | 3 | 0 | 0 | 0 | 0 | 0 | 0 | 3 | 0 |
| ***fervidus*** | 587 | 169 | 187 | 167 | 106 | 20 | 126 | 607 | 295 |
| ***flavidus*** | 311 | 97 | 57 | 35 | 24 | 22 | 46 | 333 | 143 |
| ***flavifrons*** | 350 | 123 | 97 | 79 | 49 | 18 | 67 | 368 | 190 |
| ***franklini*** | 7 | 0 | 0 | 0 | 0 | 0 | 0 | 7 | 0 |
| ***fraternus*** | 214 | 15 | 33 | 28 | 21 | 5 | 26 | 219 | 41 |
| ***frigidus*** | 253 | 83 | 16 | 8 | 1 | 8 | 9 | 261 | 92 |
| ***griseocollis*** | 501 | 170 | 299 | 256 | 161 | 43 | 204 | 544 | 374 |
| ***huntii*** | 269 | 81 | 126 | 101 | 69 | 25 | 94 | 294 | 175 |
| ***impatiens*** | 400 | 171 | 326 | 265 | 126 | 61 | 187 | 461 | 358 |
| ***insularis*** | 361 | 105 | 90 | 64 | 41 | 26 | 67 | 387 | 172 |
| ***jonellus*** | 95 | 38 | 1 | 1 | 1 | 0 | 1 | 95 | 39 |
| ***kirbiellus*** | 113 | 34 | 7 | 3 | 1 | 4 | 5 | 117 | 39 |
| ***kluanensis*** | 5 | 4 | 0 | 0 | 0 | 0 | 0 | 5 | 4 |
| ***melanopygus*** | 283 | 102 | 104 | 71 | 42 | 33 | 75 | 316 | 177 |
| ***mixtus*** | 308 | 117 | 89 | 75 | 37 | 14 | 51 | 322 | 168 |
| ***morrisoni*** | 183 | 10 | 37 | 29 | 25 | 8 | 33 | 191 | 43 |
| ***natvigi*** | 50 | 10 | 0 | 0 | 0 | 0 | 0 | 50 | 9 |
| ***neoboreus*** | 41 | 12 | 0 | 0 | 0 | 0 | 0 | 41 | 12 |
| ***nevadensis*** | 250 | 64 | 120 | 86 | 64 | 34 | 98 | 284 | 162 |
| ***occidentalis*** | 369 | 131 | 98 | 93 | 45 | 5 | 50 | 374 | 181 |
| ***pensylvanicus*** | 558 | 100 | 134 | 124 | 88 | 10 | 98 | 568 | 198 |
| ***perplexus*** | 252 | 138 | 127 | 84 | 30 | 43 | 73 | 295 | 211 |
| ***polaris*** | 113 | 30 | 1 | 0 | 0 | 1 | 1 | 114 | 30 |
| ***rufocinctus*** | 402 | 142 | 191 | 145 | 79 | 46 | 125 | 448 | 267 |
| ***sandersoni*** | 184 | 85 | 20 | 11 | 8 | 9 | 17 | 193 | 102 |
| ***sitkensis*** | 114 | 26 | 29 | 29 | 19 | 0 | 19 | 114 | 45 |
| ***sp.*** | 0 | 0 | 419 | 0 | 0 | 419 | 419 | 419 | 419 |
| ***suckleyi*** | 202 | 11 | 2 | 2 | 2 | 0 | 2 | 202 | 13 |
| ***sylvicola*** | 309 | 84 | 36 | 19 | 12 | 17 | 29 | 326 | 112 |
| ***ternarius*** | 300 | 149 | 235 | 178 | 75 | 57 | 132 | 357 | 281 |
| ***terricola*** | 383 | 135 | 155 | 127 | 50 | 28 | 78 | 411 | 213 |
| ***vagans*** | 406 | 200 | 179 | 140 | 57 | 39 | 96 | 445 | 296 |
| ***vagans, sandersoni or perplexus*** | 0 | 0 | 149 | 0 | 0 | 149 | 149 | 149 | 149 |
| ***vandykei*** | 56 | 16 | 18 | 15 | 8 | 3 | 11 | 59 | 27 |
| ***variabilis*** | 94 | 0 | 0 | 0 | 0 | 0 | 0 | 94 | 0 |
| ***vosnesenskii*** | 98 | 31 | 73 | 60 | 34 | 13 | 47 | 111 | 78 |
| ***vosnesenskii or caliginosus*** | 0 | 0 | 49 | 0 | 0 | 49 | 49 | 49 | 49 |
| **all species combined** | 1434 | 619 | 842 | 792^+^  717¨ | 384^+^  543¨ | 50^+^  604¨ | 398^+^  764¨ | 1484 | 1017 |
| **Mean ± standard error (se) (excl combined species)** | 198 ± 21.3 | 62 ± 8.1 | 94 ± 13.1 | 51.9 ± 9.4 | 27 ± 5.1 | 42± 8.6 | 69 ± 10.2 | 240 ± 22.2 | 131 ± 15.3 |
| **Mann-Whitney U-test (U)** | Total # grids,  BBNA all years vs BBW  U=1947, p<0.001* | Total # grids,  BBNA 2010-2020 vs BBW  U=1232, p=0.649 |  |  |  | Unique grids from BBW, all years vs 2010-2020 combined datasets  U=821.5, p=0.001* | | Total number of grids, all years vs 2010-2020 combined datasets  U=1875.5, p<0.001* | |
| * Number of grids out of the 100km x 100km grids in BBNA all years (excluding grids unique to BBW)  ^+^ Number of grids overall (grids compared with all species combined)  ¨ Number of grids for at least one species (grids compared for each species separately) | | | | | | | | | |

**S1 File Table 11. A comparison of the number of 10km x 10km grids with records per species per dataset, and the number of unique grids over the combined all years and 2010-2020 datasets due to the BBW dataset.**

| **Comparison of 10km x 10km grids being tested per *Bombus* species** | **total # 10km x 10km grids per dataset** | | | **# unique grids due to BBW over all years** | **# unique grids due to BBW over 2010-2020** | **# grids with any records in combined all years data** | **# grids with any records in combined 2010-2020 data** |
| --- | --- | --- | --- | --- | --- | --- | --- |
| **Datasets being compared** | BBNA all years | BBNA 2010-2020 | BBW | BBW vs BBNA all years | BBW vs BBNA 2010-2020 | BBW and BBNA all years | BBW and BBNA 2010-2020 |
| ***affinis*** | 961 | 73 | 157 | 112 | 128 | 1073 | 201 |
| ***appositus*** | 665 | 76 | 199 | 167 | 192 | 832 | 268 |
| ***auricomus*** | 658 | 61 | 138 | 117 | 135 | 775 | 196 |
| ***bifarius*** | 1983 | 251 | 618 | 445 | 592 | 2428 | 843 |
| ***bimaculatus*** | 1976 | 833 | 720 | 539 | 631 | 2515 | 1464 |
| ***bohemicus*** | 645 | 36 | 6 | 5 | 5 | 650 | 41 |
| ***borealis*** | 910 | 360 | 390 | 316 | 356 | 1226 | 716 |
| ***caliginosus*** | 294 | 11 | 49 | 38 | 47 | 332 | 58 |
| ***centralis*** | 988 | 149 | 364 | 317 | 352 | 1305 | 501 |
| ***citrinus*** | 830 | 177 | 88 | 70 | 82 | 900 | 259 |
| ***cockerelli*** | 7 | 1 | 0 | 0 | 0 | 7 | 1 |
| ***crotchii*** | 295 | 4 | 13 | 10 | 13 | 305 | 17 |
| ***cryptarum*** | 218 | 125 | 69 | 56 | 59 | 274 | 184 |
| ***distinguendus*** | 4 | 0 | 0 | 0 | 0 | 4 | 0 |
| ***fervidus*** | 3146 | 418 | 530 | 410 | 508 | 3556 | 926 |
| ***flavidus*** | 650 | 177 | 129 | 116 | 124 | 766 | 301 |
| ***flavifrons*** | 1378 | 291 | 519 | 396 | 500 | 1774 | 791 |
| ***franklini*** | 39 | 0 | 0 | 0 | 0 | 39 | 0 |
| ***fraternus*** | 494 | 43 | 58 | 48 | 55 | 542 | 98 |
| ***frigidus*** | 554 | 191 | 21 | 19 | 21 | 573 | 212 |
| ***griseocollis*** | 2489 | 816 | 1010 | 813 | 939 | 3302 | 1755 |
| ***huntii*** | 1268 | 199 | 454 | 361 | 440 | 1629 | 639 |
| ***impatiens*** | 3284 | 1224 | 1721 | 1252 | 1524 | 4536 | 2748 |
| ***insularis*** | 1001 | 162 | 232 | 202 | 225 | 1203 | 387 |
| ***jonellus*** | 196 | 85 | 1 | 1 | 1 | 197 | 86 |
| ***kirbiellus*** | 221 | 61 | 7 | 6 | 7 | 227 | 68 |
| ***kluanensis*** | 8 | 6 | 0 | 0 | 0 | 8 | 6 |
| ***melanopygus*** | 1346 | 223 | 439 | 346 | 424 | 1692 | 647 |
| ***mixtus*** | 1182 | 263 | 473 | 365 | 446 | 1547 | 709 |
| ***morrisoni*** | 840 | 15 | 52 | 39 | 50 | 879 | 65 |
| ***natvigi*** | 62 | 11 | 0 | 0 | 0 | 62 | 11 |
| ***neoboreus*** | 57 | 20 | 0 | 0 | 0 | 57 | 20 |
| ***nevadensis*** | 716 | 130 | 387 | 326 | 373 | 1042 | 503 |
| ***occidentalis*** | 2228 | 326 | 289 | 184 | 258 | 2412 | 584 |
| ***pensylvanicus*** | 3025 | 312 | 284 | 203 | 268 | 3228 | 580 |
| ***perplexus*** | 1312 | 511 | 268 | 204 | 233 | 1516 | 744 |
| ***polaris*** | 160 | 46 | 1 | 1 | 1 | 161 | 47 |
| ***rufocinctus*** | 1451 | 385 | 611 | 512 | 562 | 1963 | 947 |
| ***sandersoni*** | 539 | 271 | 31 | 28 | 31 | 567 | 302 |
| ***sitkensis*** | 390 | 58 | 93 | 76 | 90 | 466 | 148 |
| ***sp.*** | 0 | 0 | 1447 | 1447 | 1447 | 1447 | 1447 |
| ***suckleyi*** | 467 | 11 | 2 | 2 | 2 | 469 | 13 |
| ***sylvicola*** | 713 | 162 | 53 | 48 | 52 | 761 | 214 |
| ***ternarius*** | 1557 | 689 | 1016 | 836 | 923 | 2393 | 1612 |
| ***terricola*** | 1894 | 443 | 374 | 294 | 337 | 2188 | 780 |
| ***vagans*** | 2382 | 984 | 451 | 368 | 406 | 2750 | 1390 |
| ***vagans, sandersoni or perplexus*** | 0 | 0 | 332 | 332 | 332 | 332 | 332 |
| ***vandykei*** | 245 | 23 | 57 | 46 | 54 | 291 | 77 |
| ***variabilis*** | 147 | 0 | 0 | 0 | 0 | 147 | 0 |
| ***vosnesenskii*** | 1338 | 99 | 526 | 370 | 507 | 1708 | 606 |
| ***vosnesenskii or caliginosus*** | 0 | 0 | 271 | 271 | 271 | 271 | 271 |
| **all species combined** | 15727 | 3464 | 6169 | 5513 | 5948 | 19314 | 8922 |
| **Mean ± standard error (se) (excl combined species)** | 925 ± 123.1 | 212 ± 38.4 | 293 ± 51.8 | 237.6 ± 42.9 | 274 ± 48.2 | 1163 ± 149.9 | 486 ± 78.9 |
| **Mann-Whitney U-test (U)** | Total # grids,  BBNA all years vs BBW  U=1937, p<0.001* | Total # grids,  BBNA 2010-2020 vs BBW  U=1173, p=0.395 | Unique grids from BBW, all years vs 2010-2020 combined datasets  U=1217, p=0.578 | | Total number of grids, all years vs 2010-2020 combined datasets  U=1847, p<0.001* | |  |

S1 File Fig 1

a) *Bombus affinis*

*
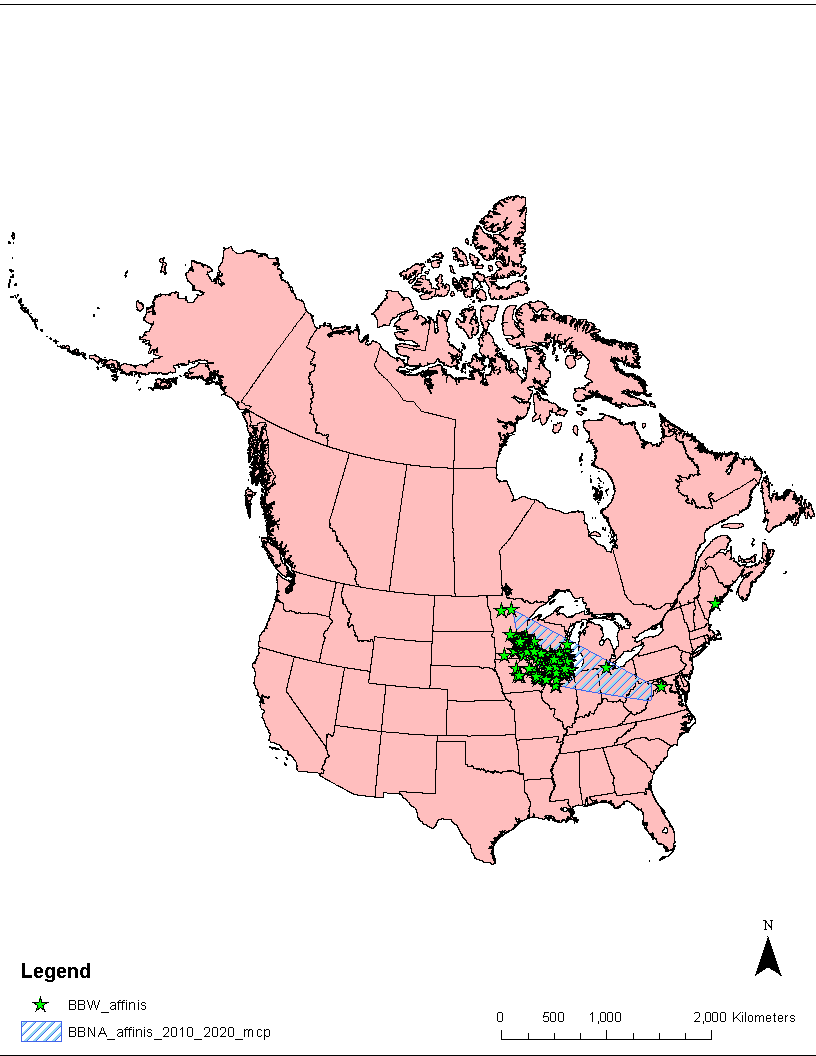

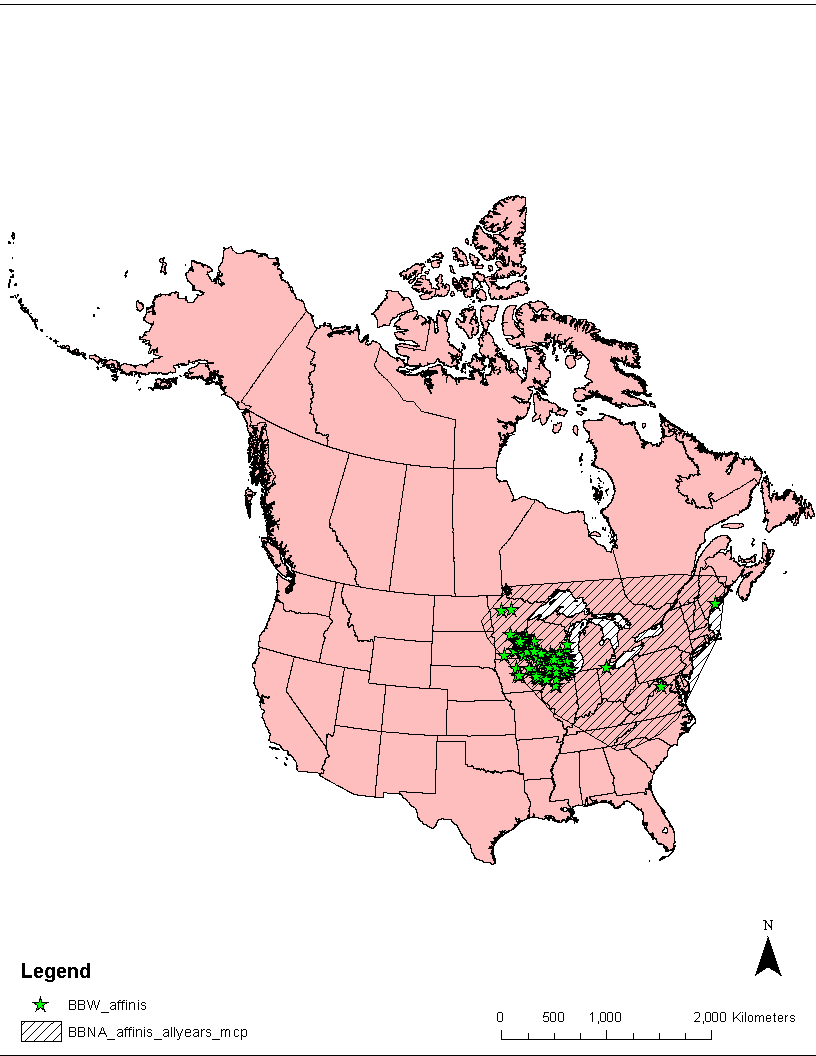
*

b) *Bombus appositus*

*
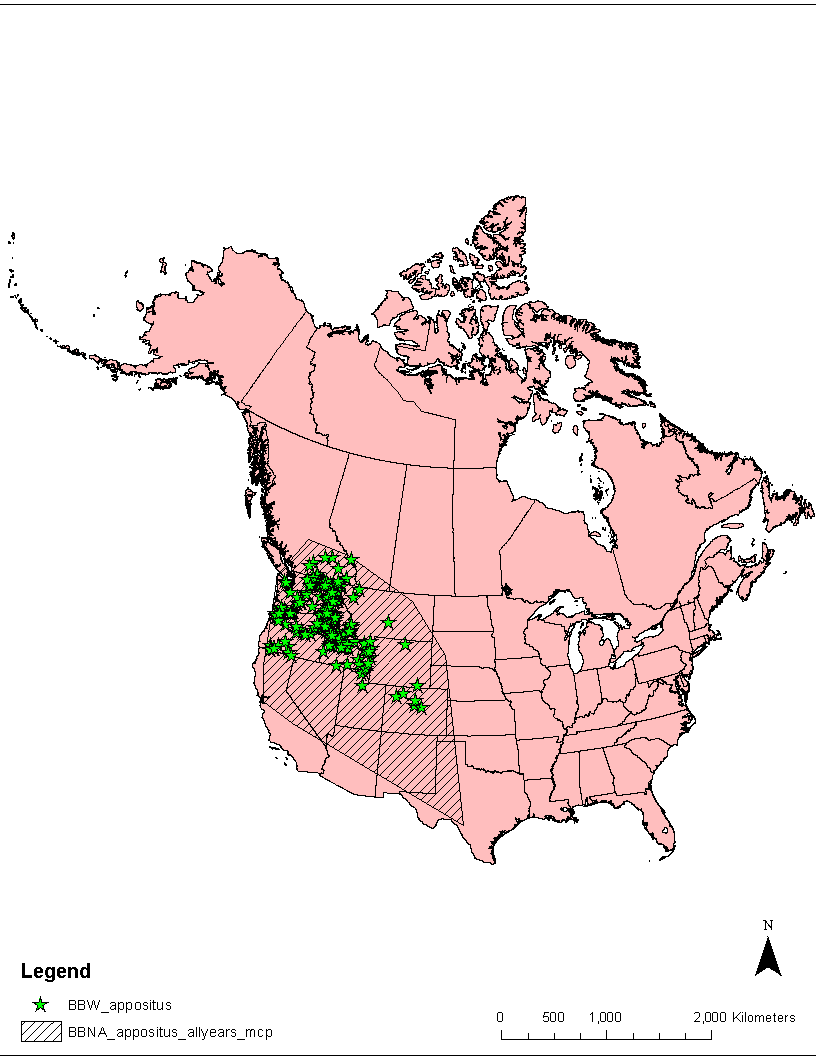

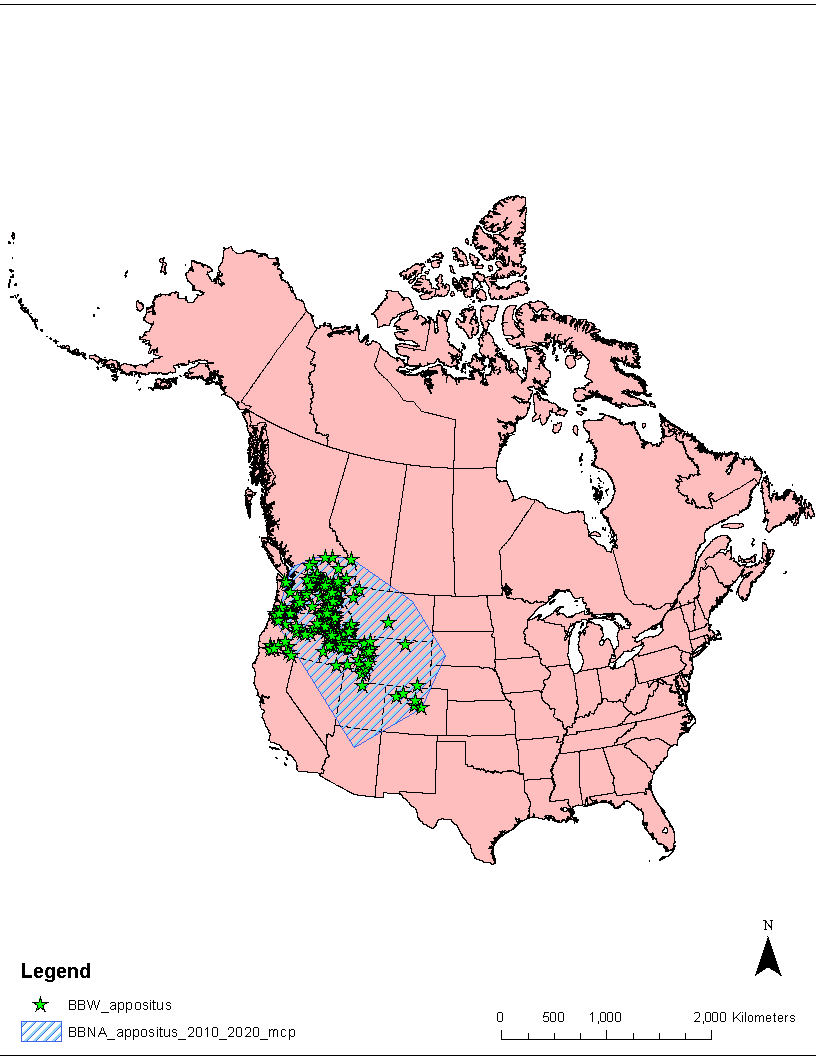
*

c) *Bombus auricomus*

*
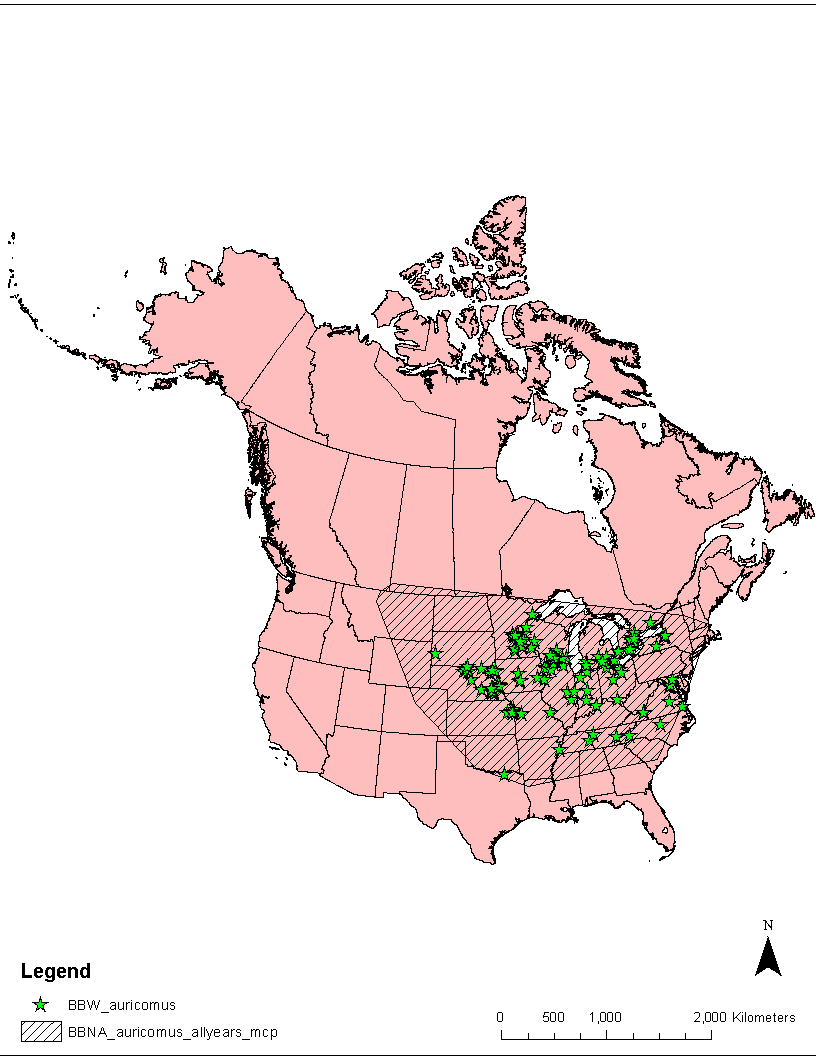

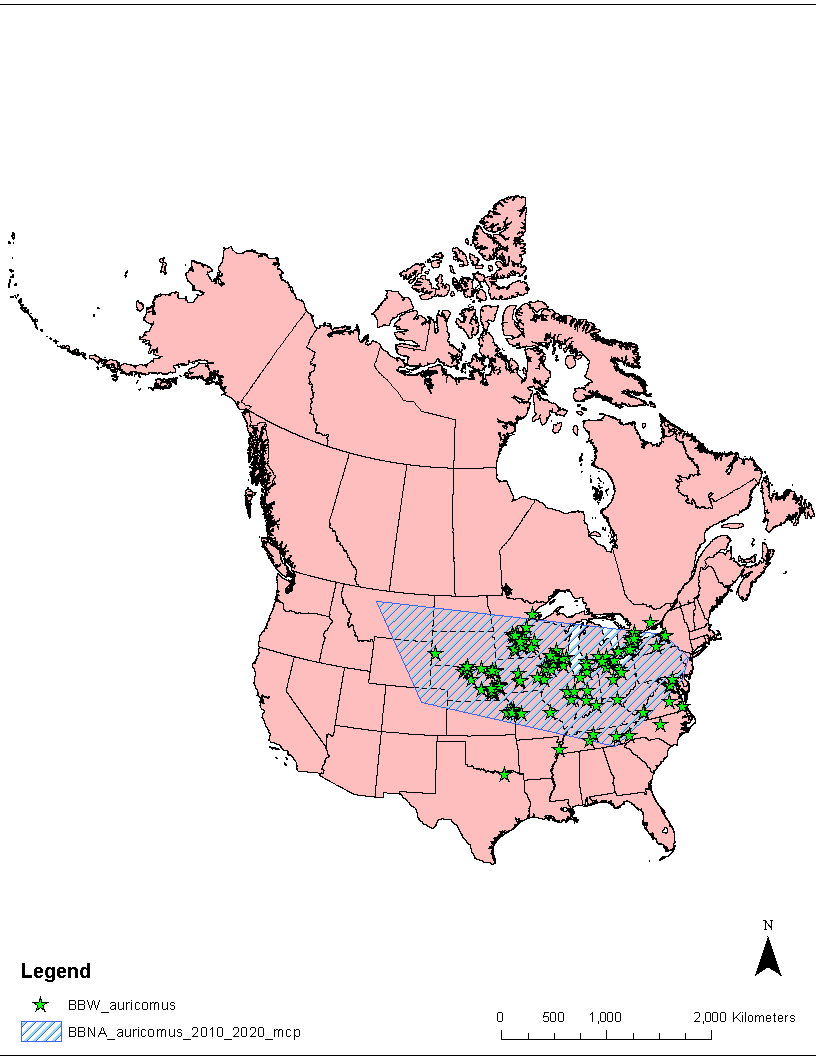
*

d) *Bombus bifarius*

*
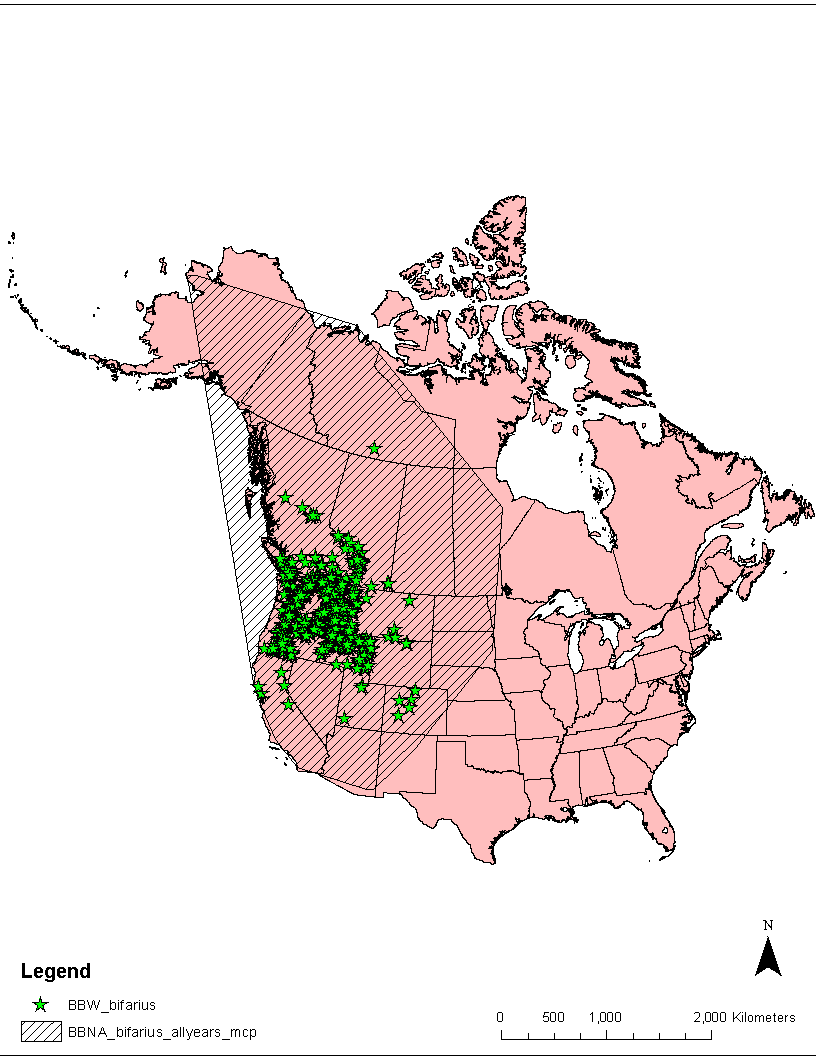

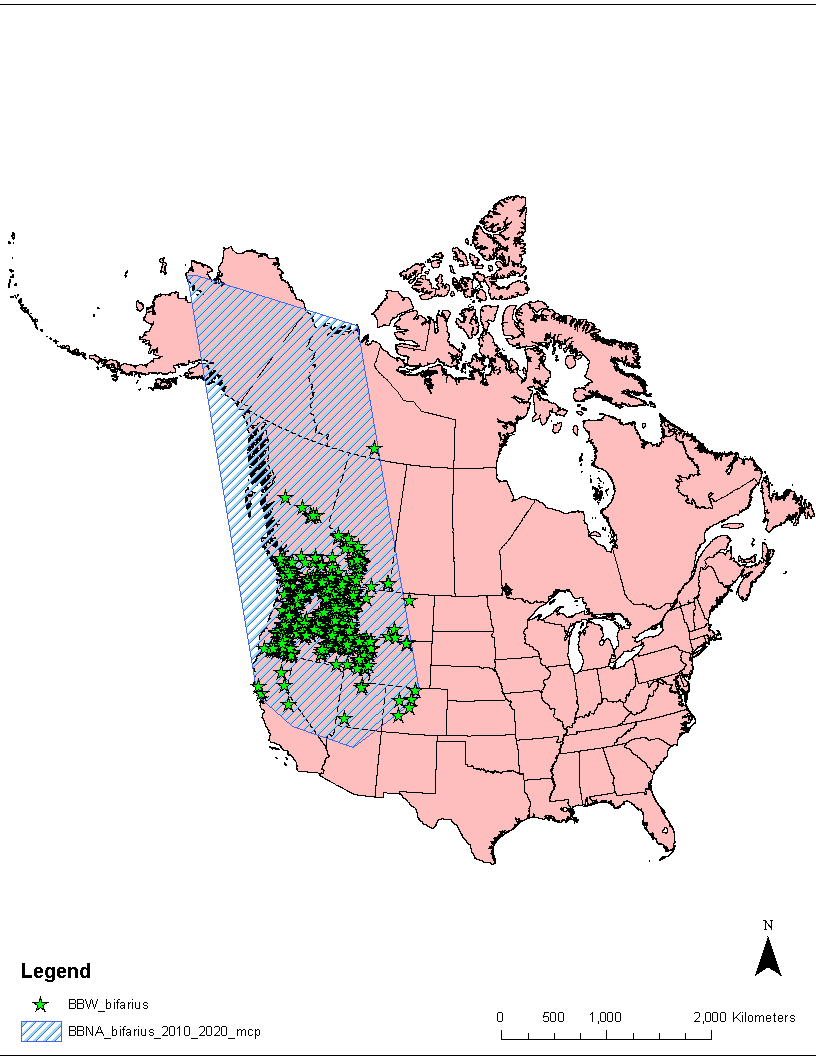
*

e) *Bombus bimaculatus*

*
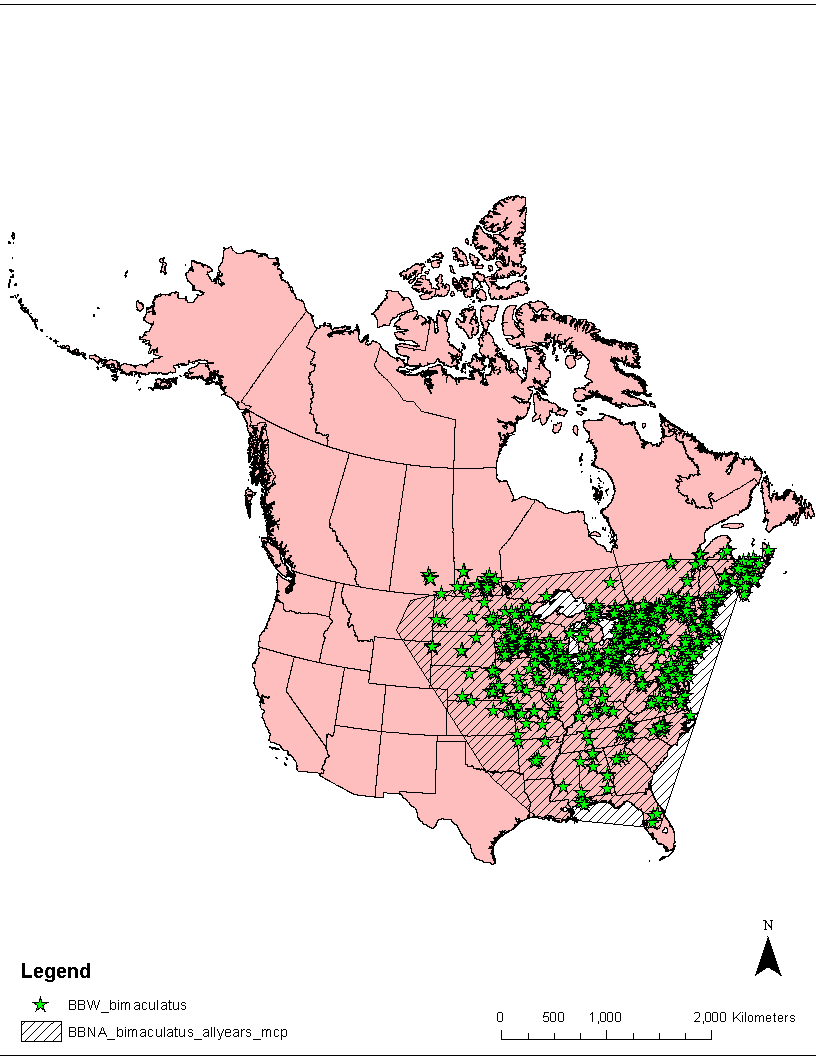

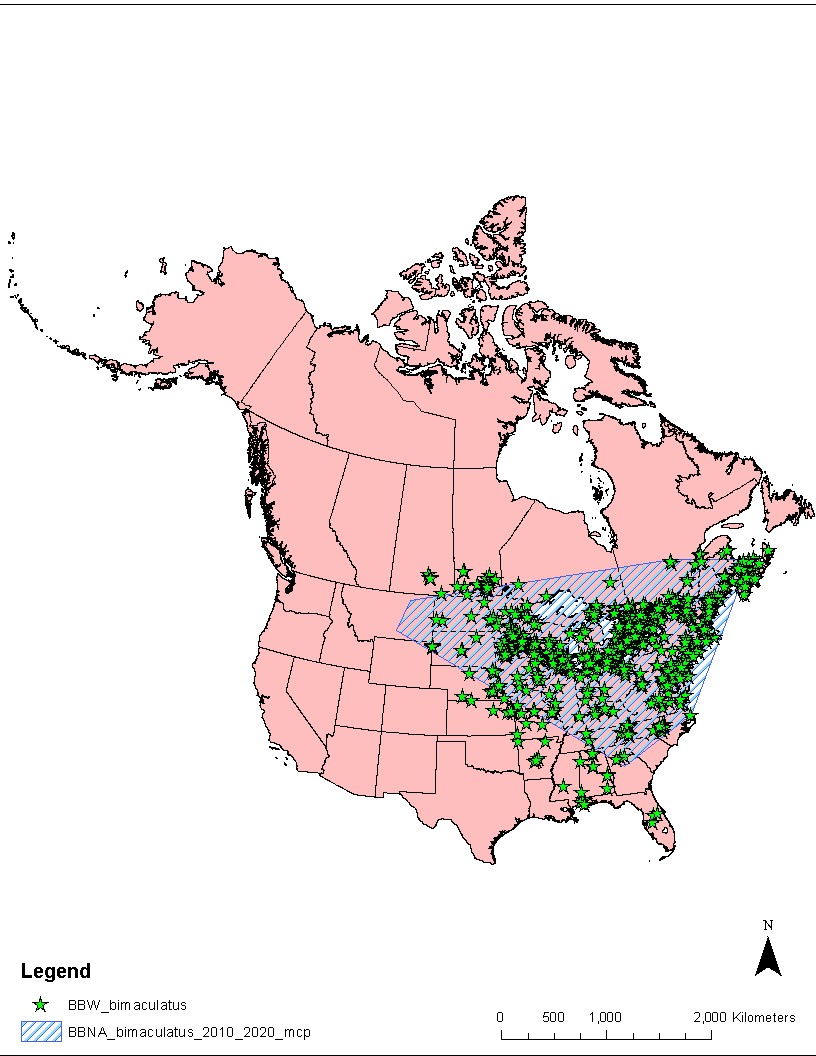
*

f) *Bombus bohemicus*

*
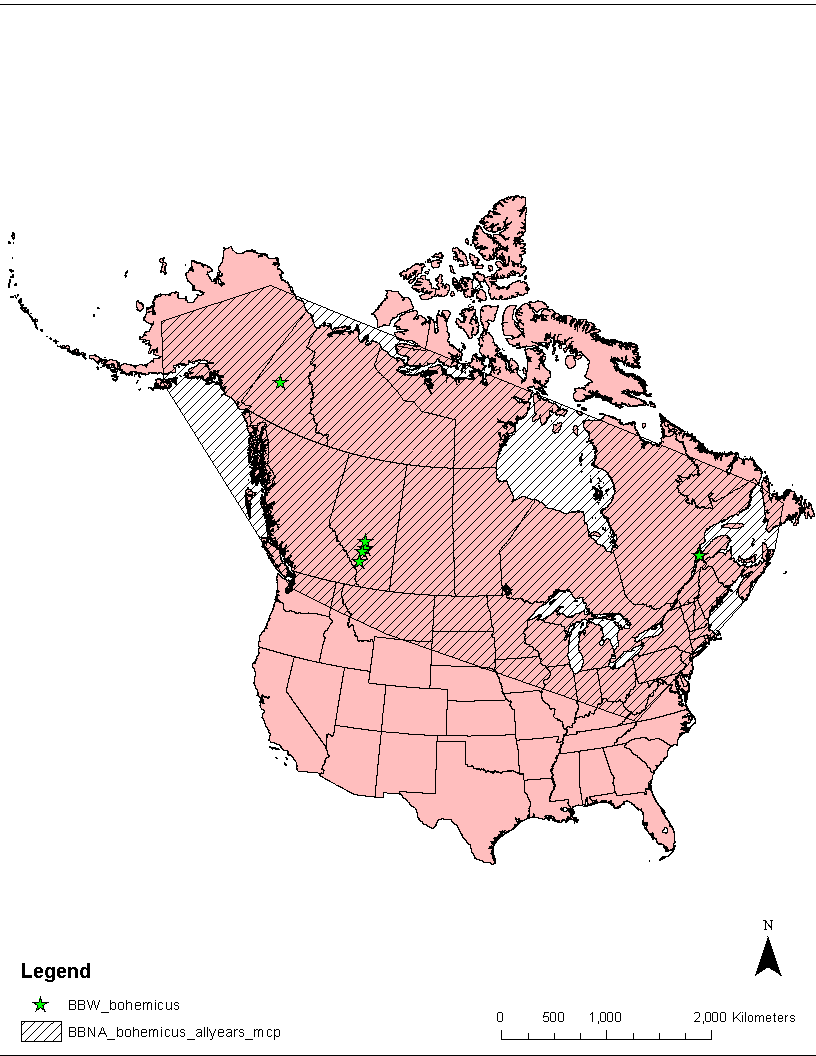

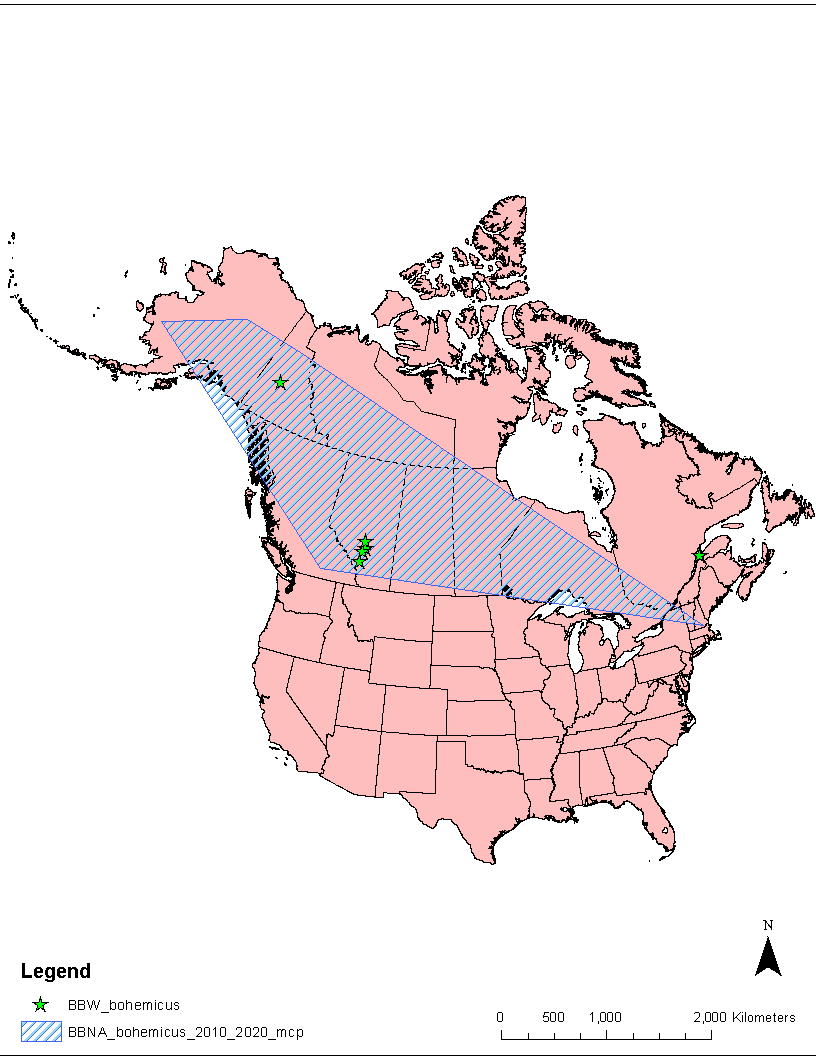
*

g) *Bombus borealis*

*
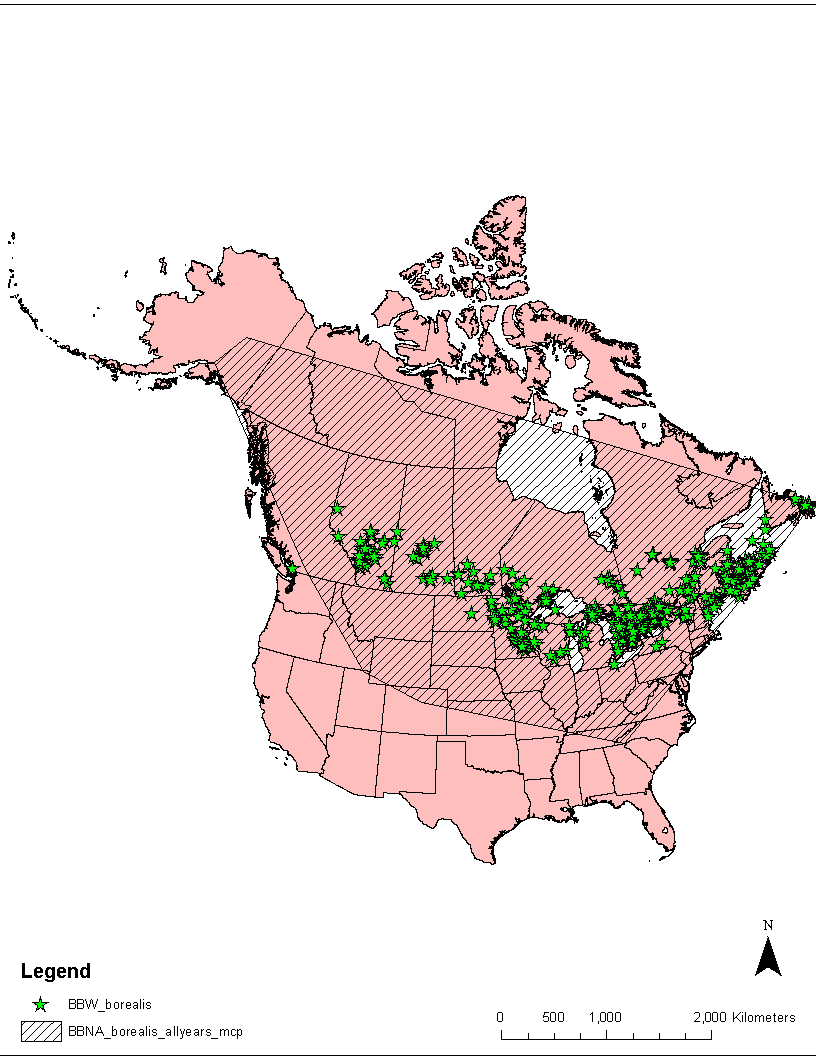

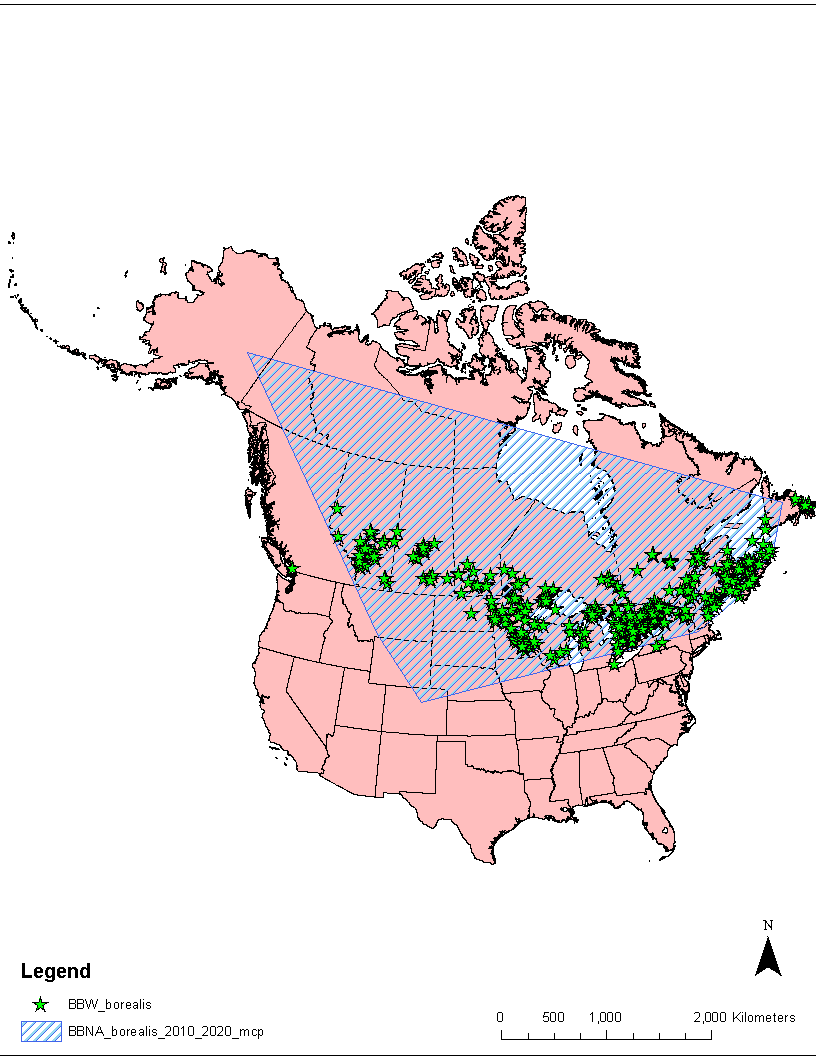
*

h) *Bombus caliginosus*

*
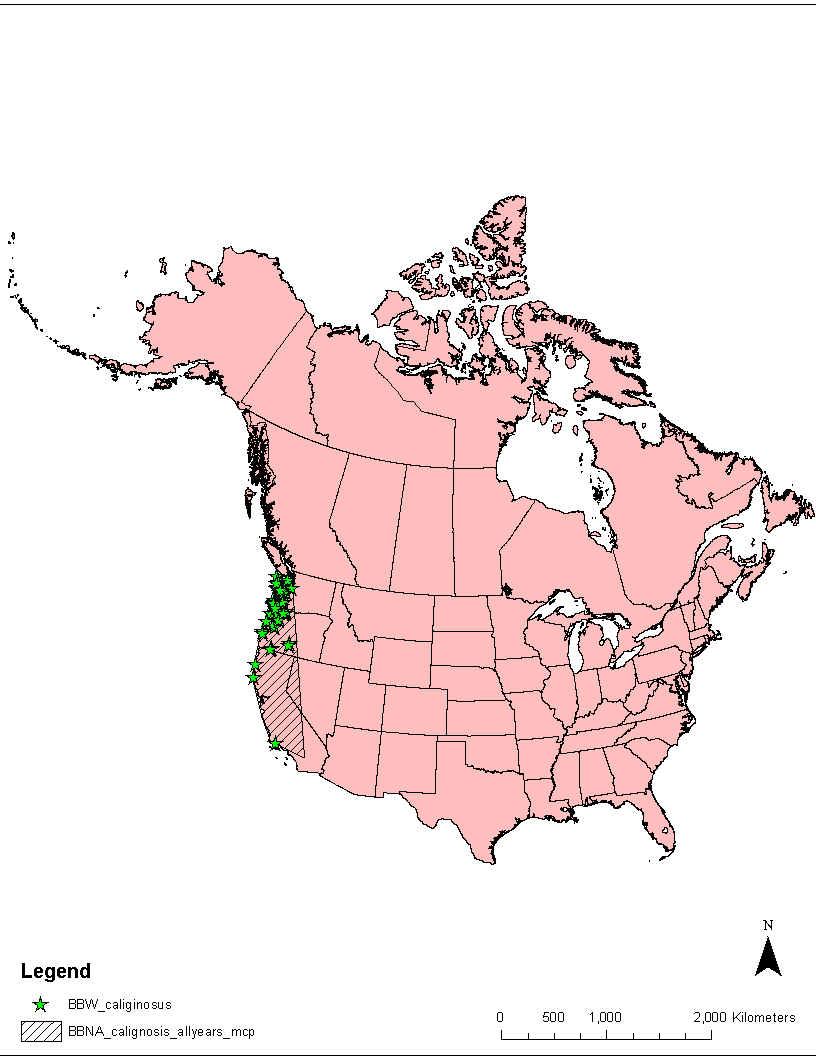

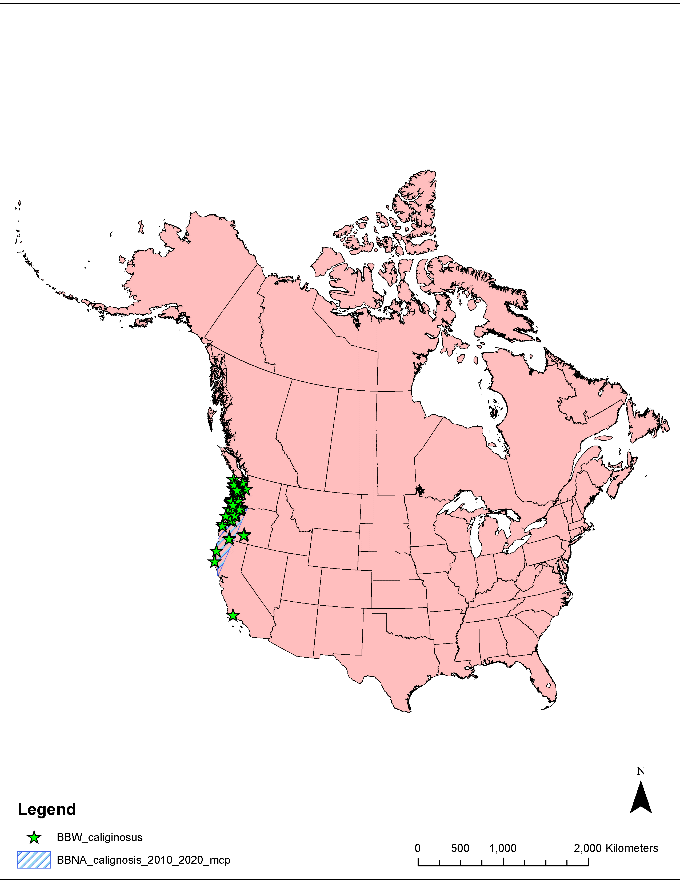
*

i) *Bombus centralis*

*
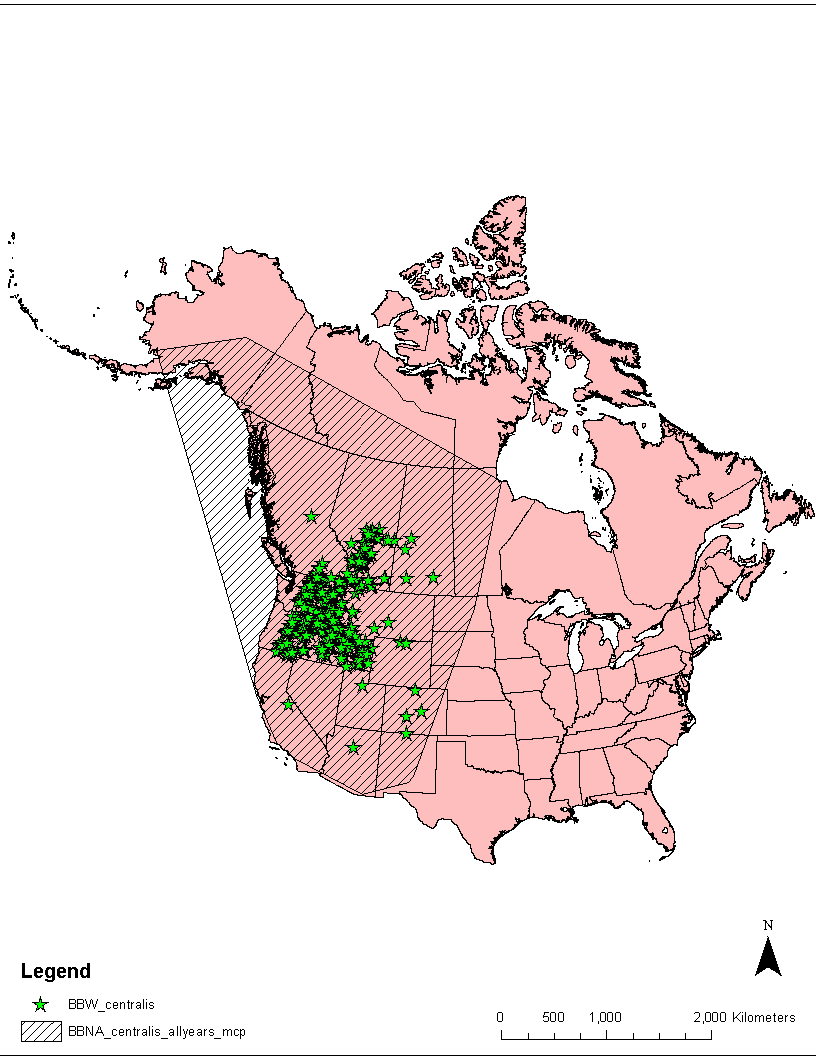

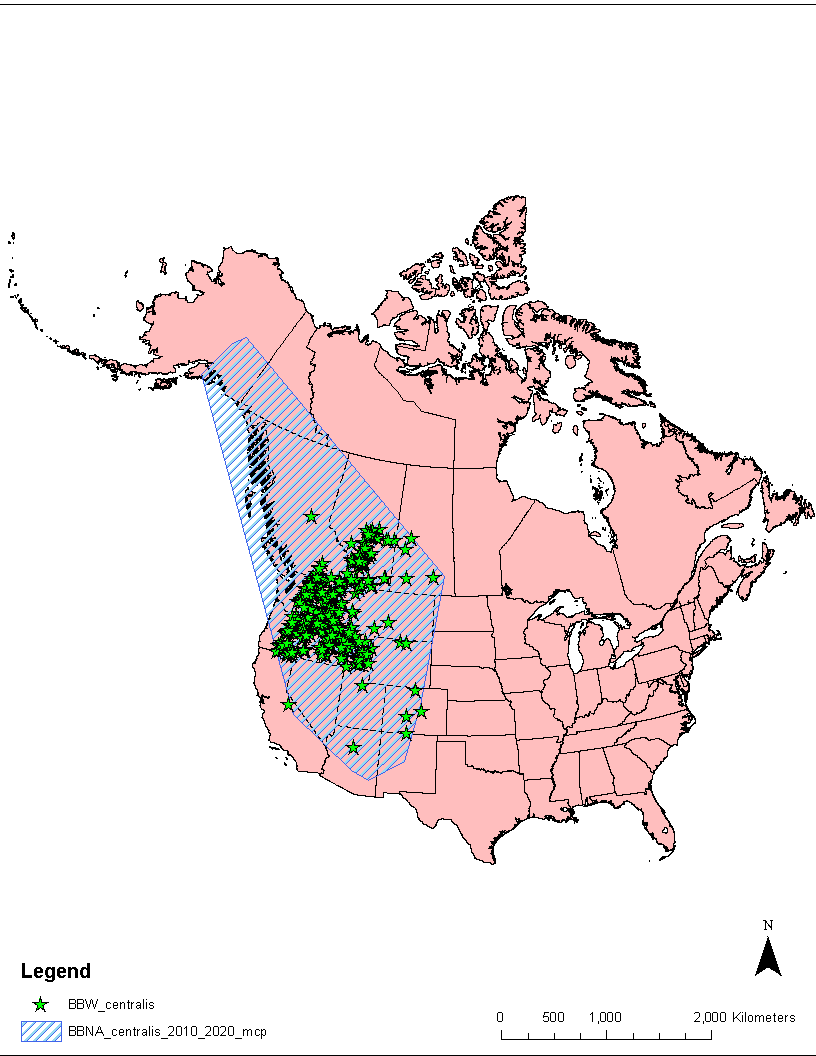
*

j) *Bombus citrinus*

*
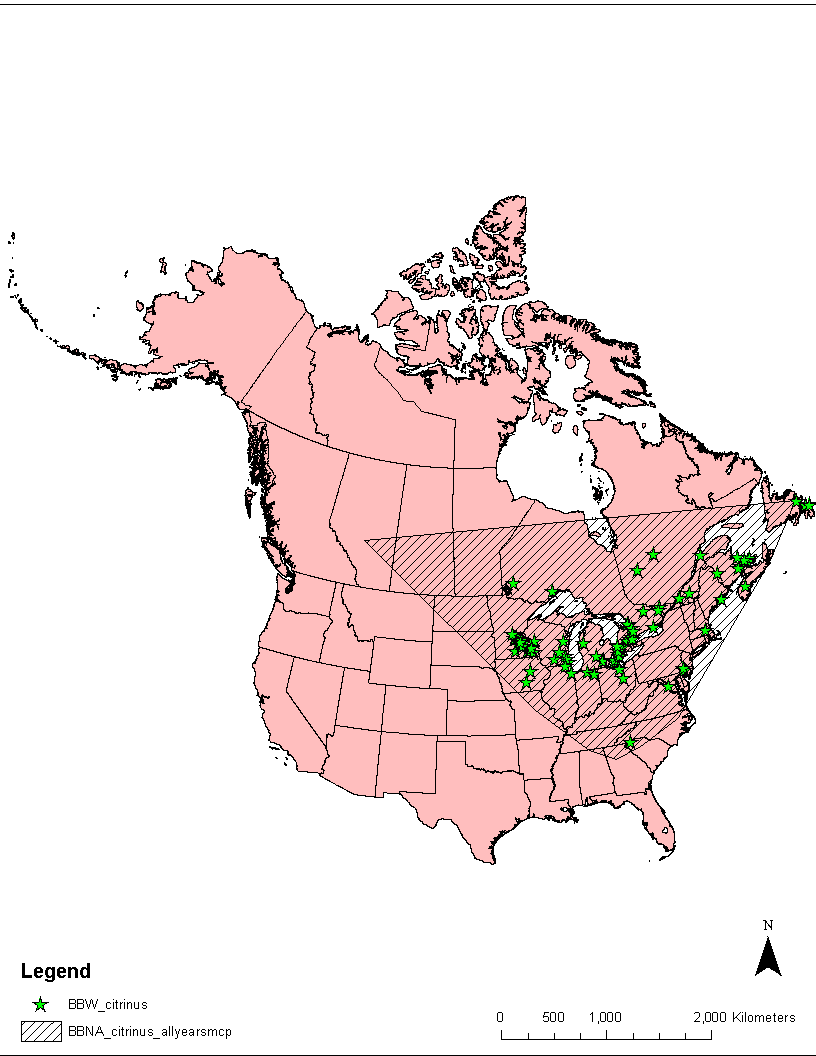

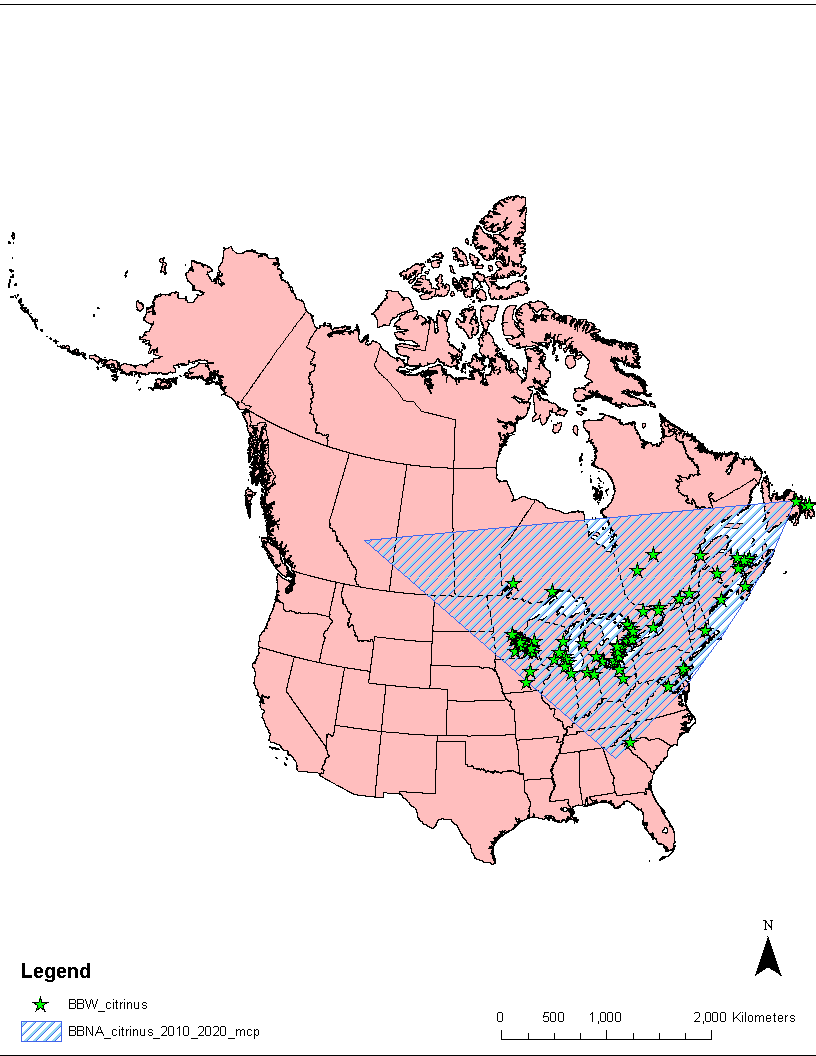
*

k) *Bombus crotchii*

*
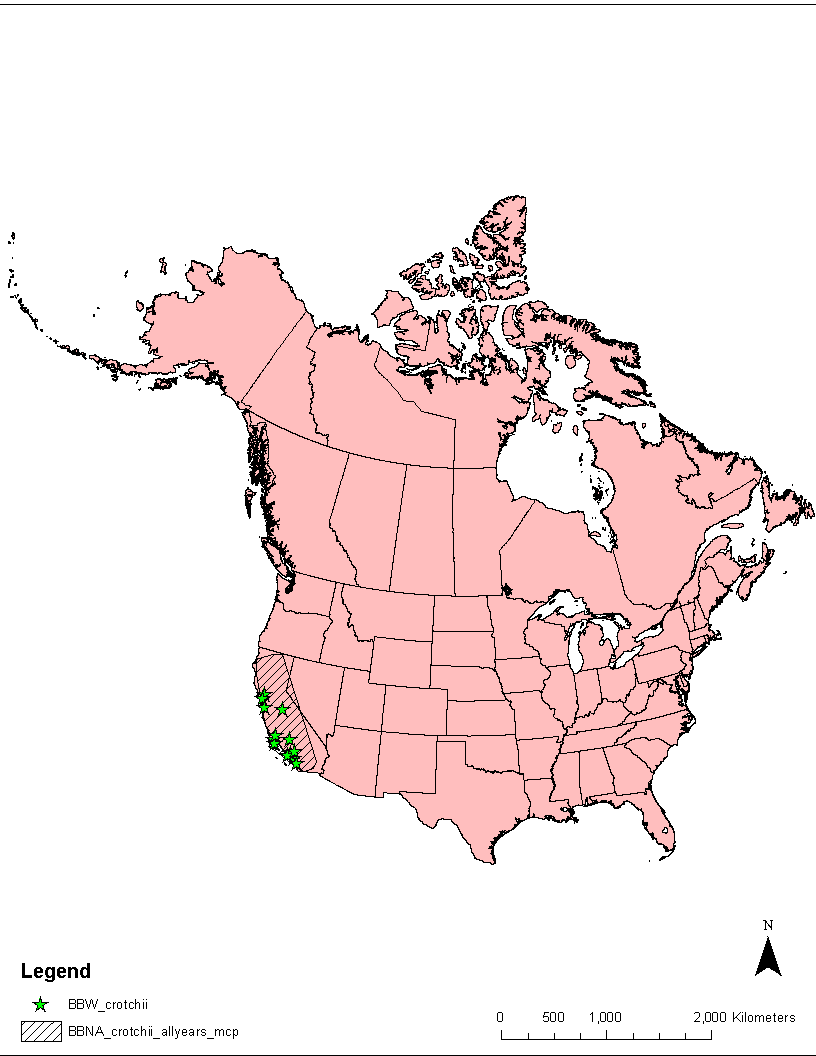

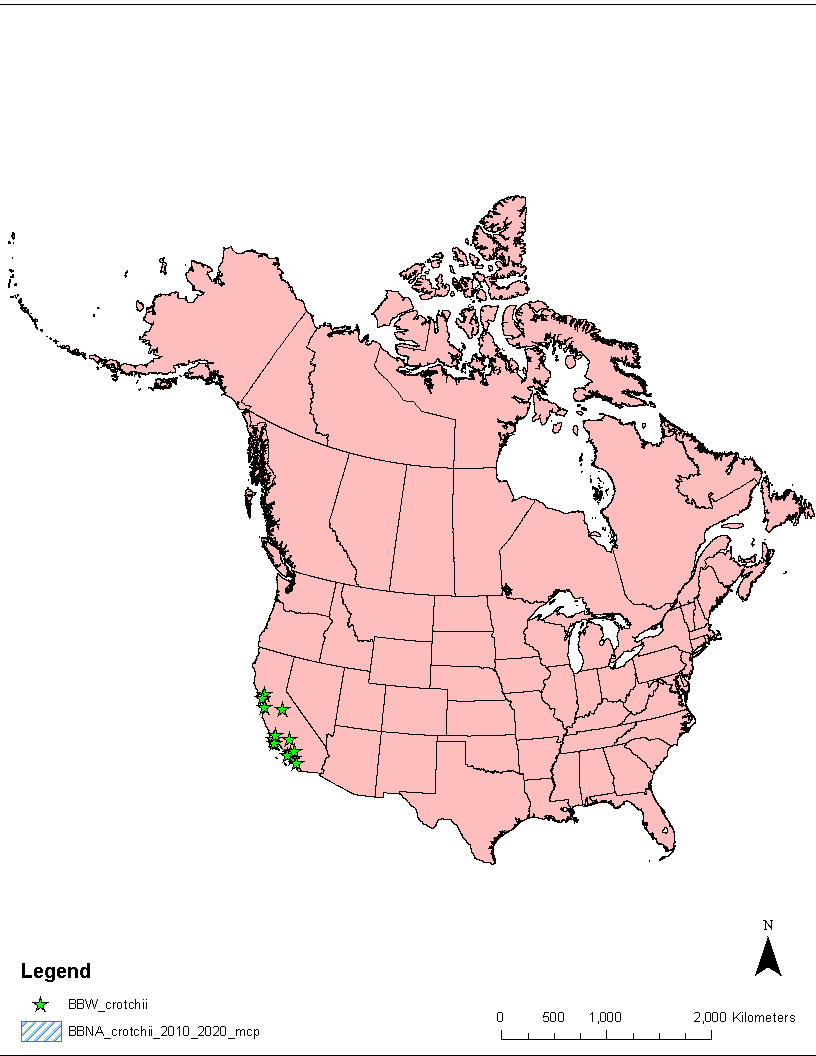
*

l) *Bombus cryptarum*

*
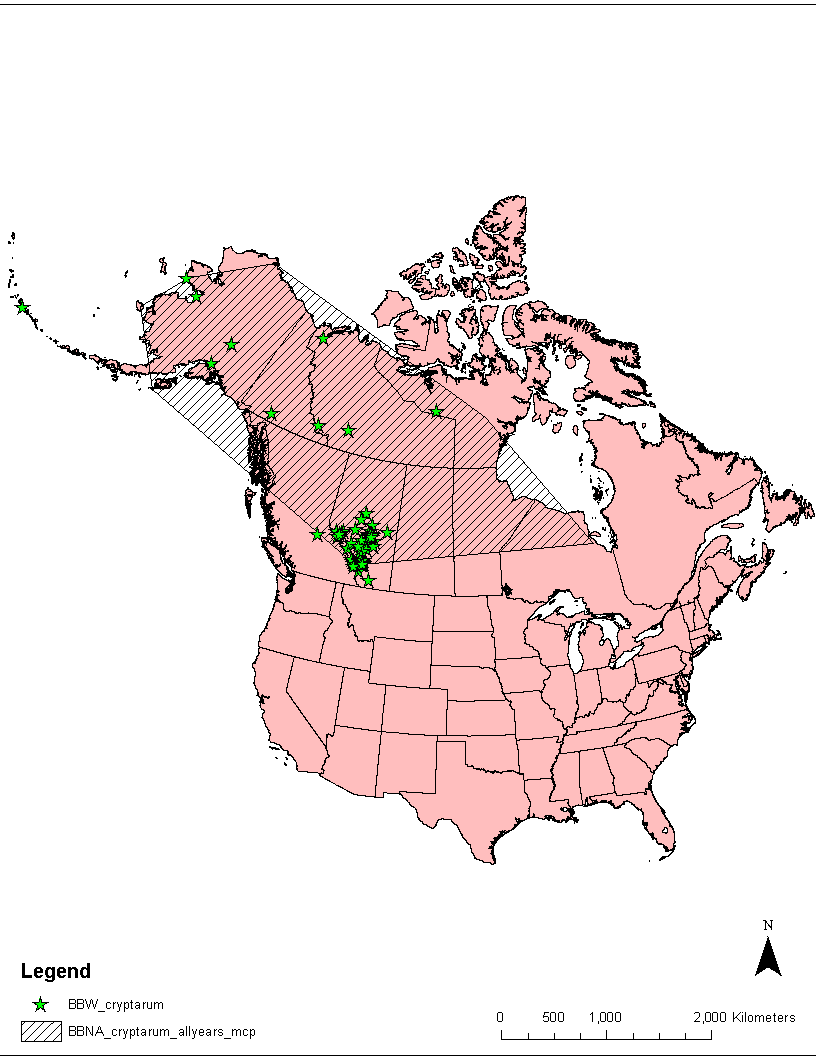

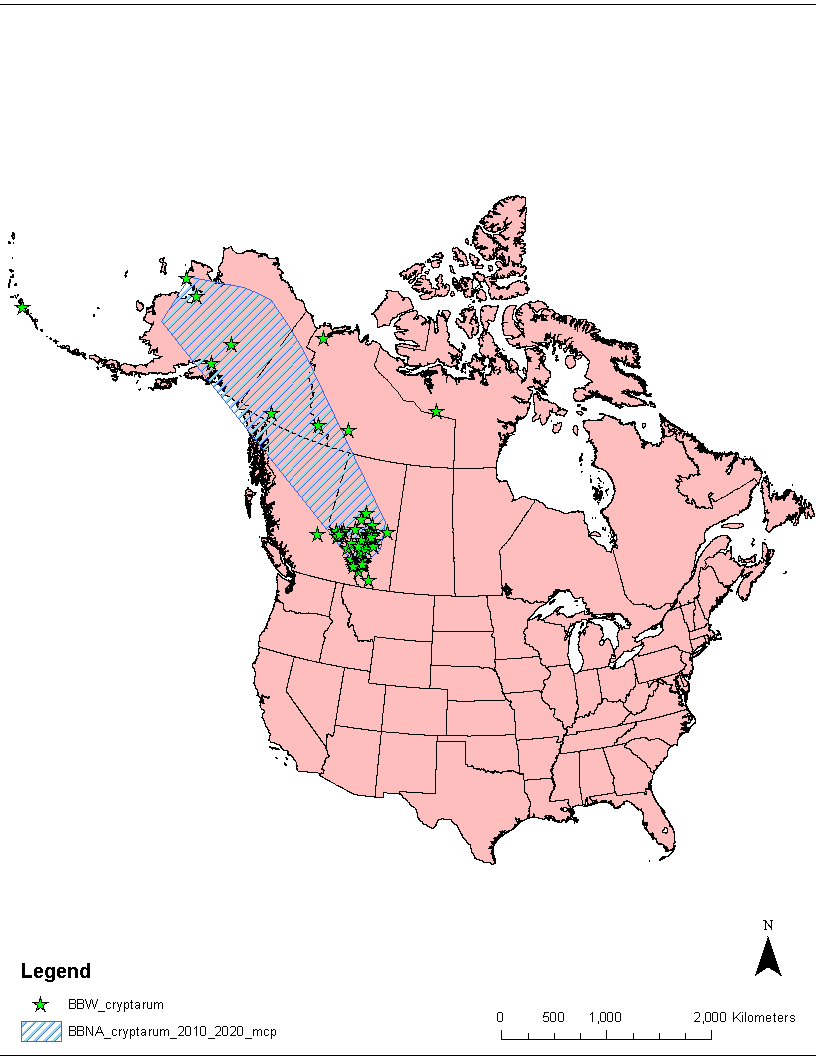
*

m) *Bombus fervidus*

*
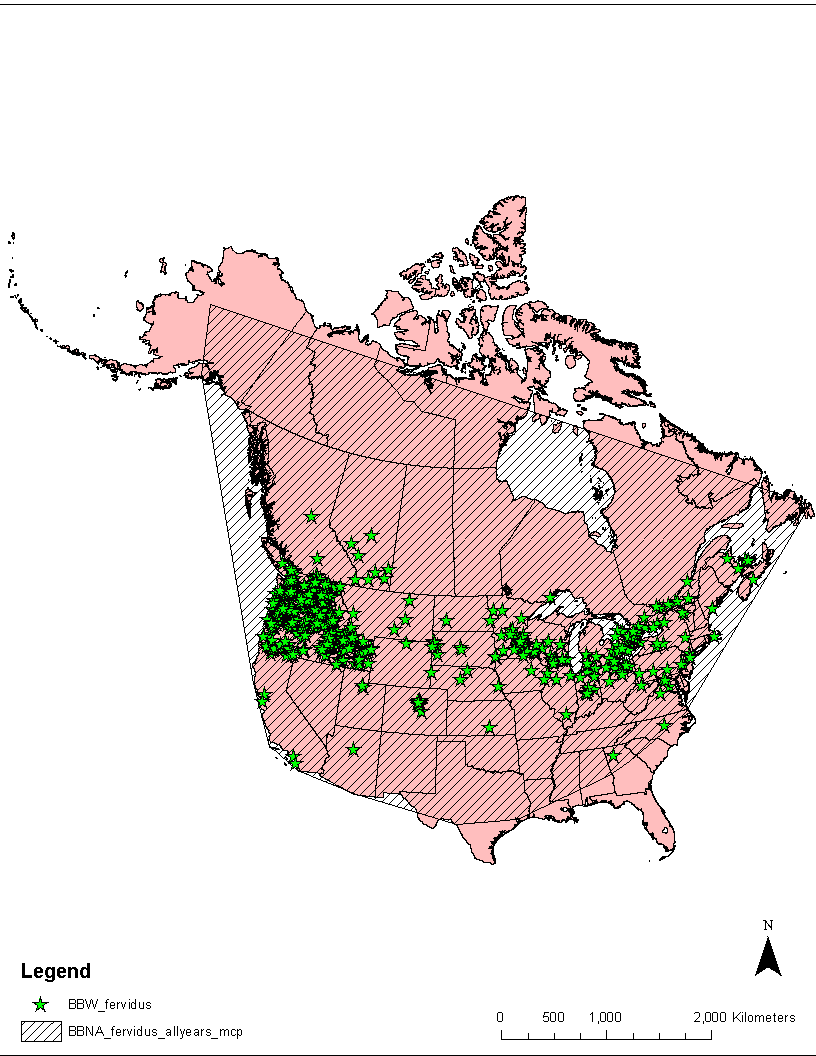

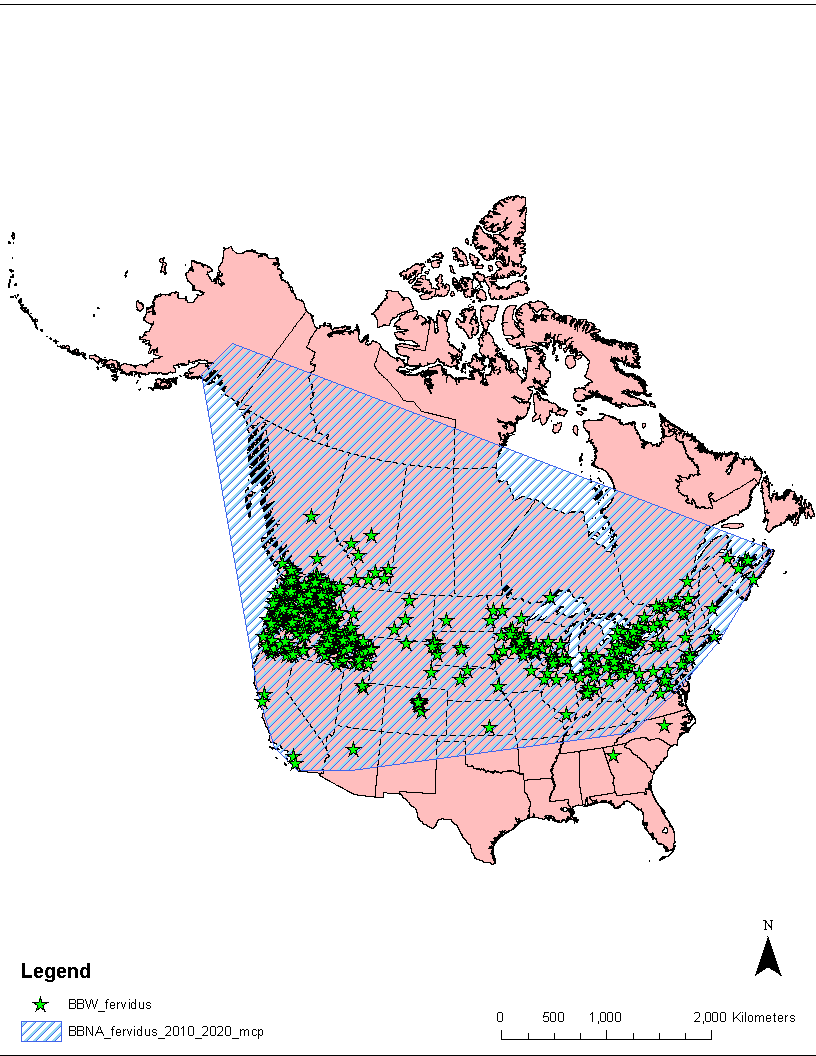
*

n) *Bombus flavidus*

*
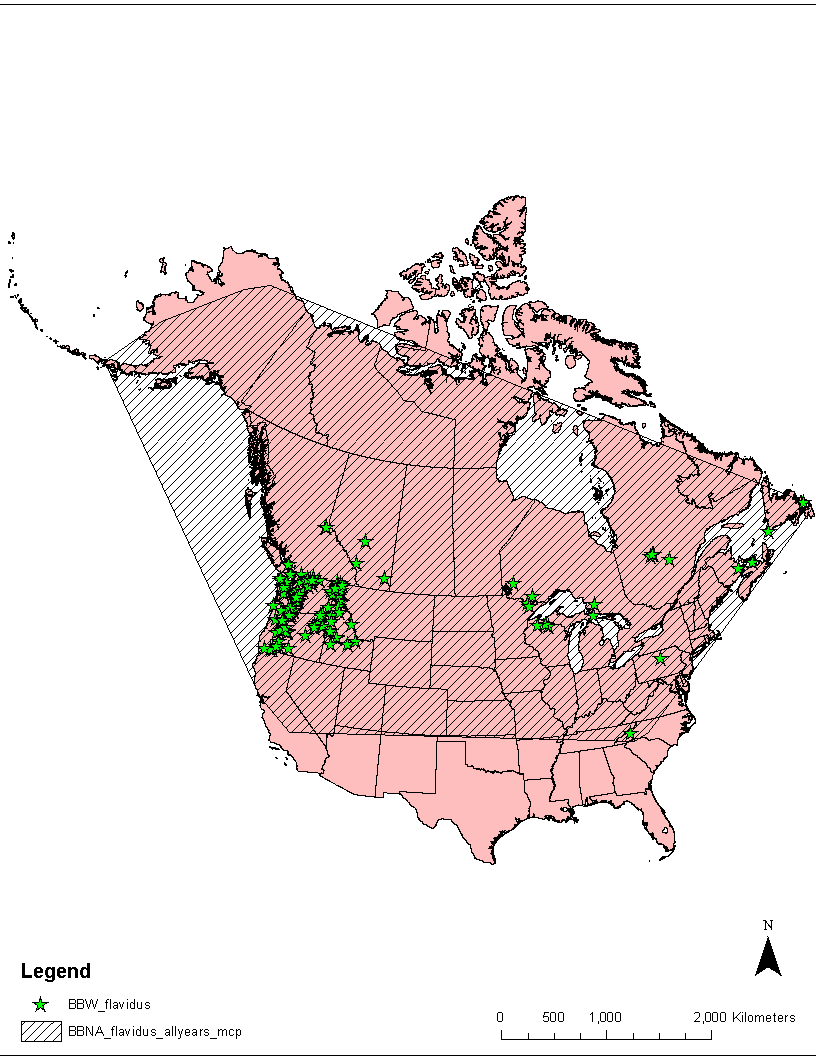

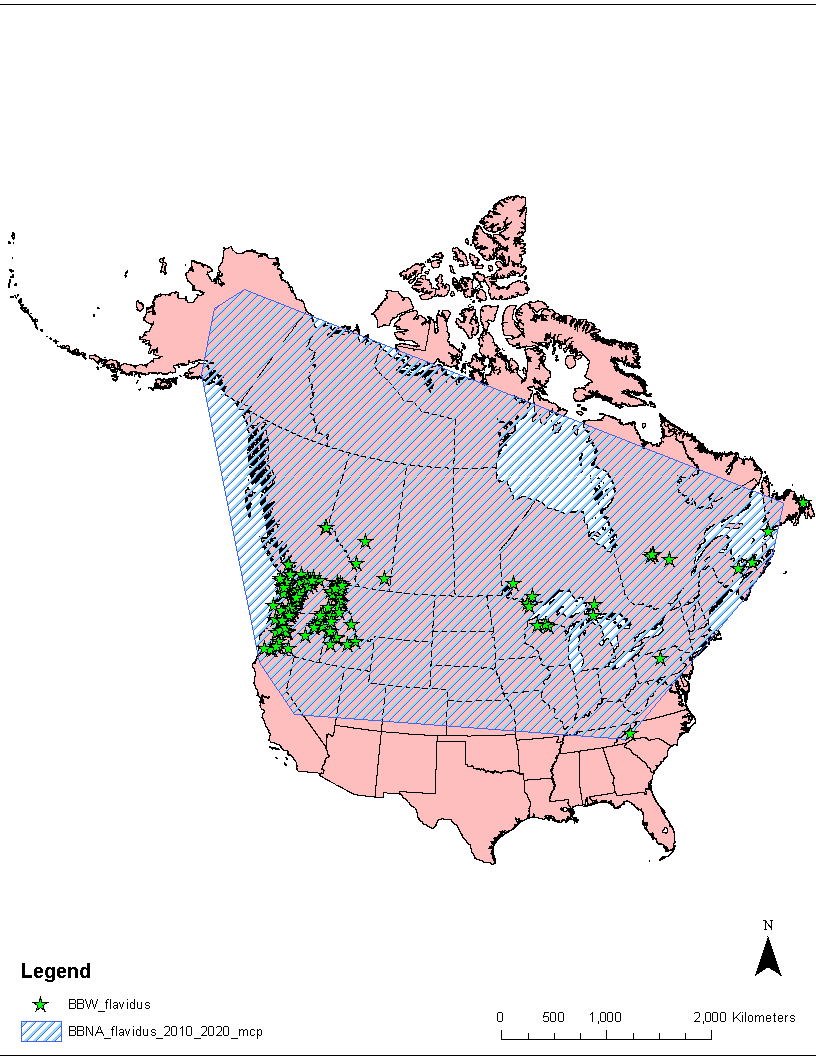
*

o) *Bombus flavifrons*

*
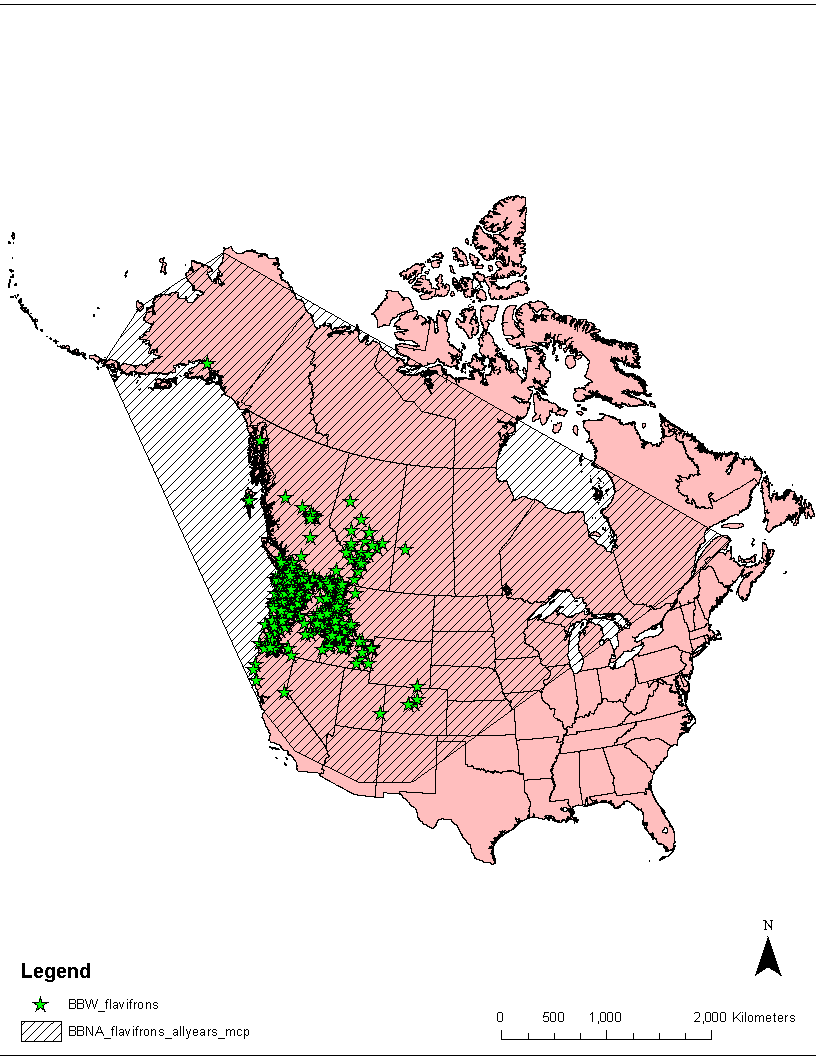

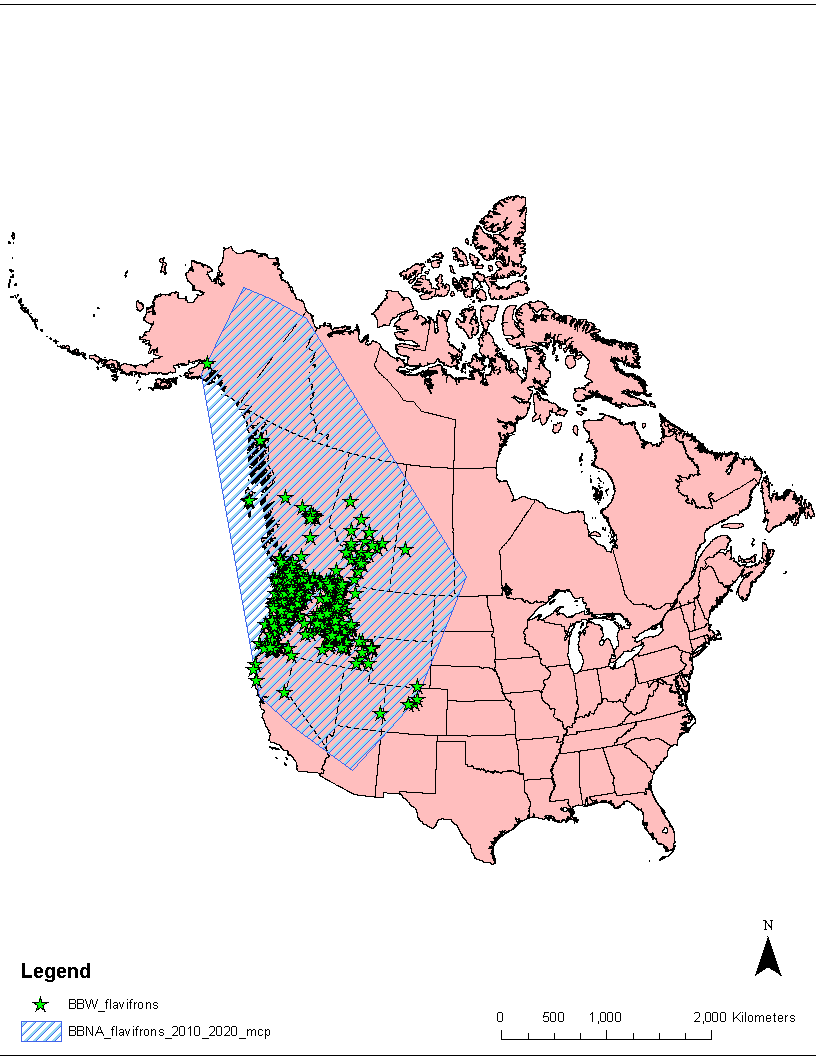
*

p) *Bombus fraternus*

*
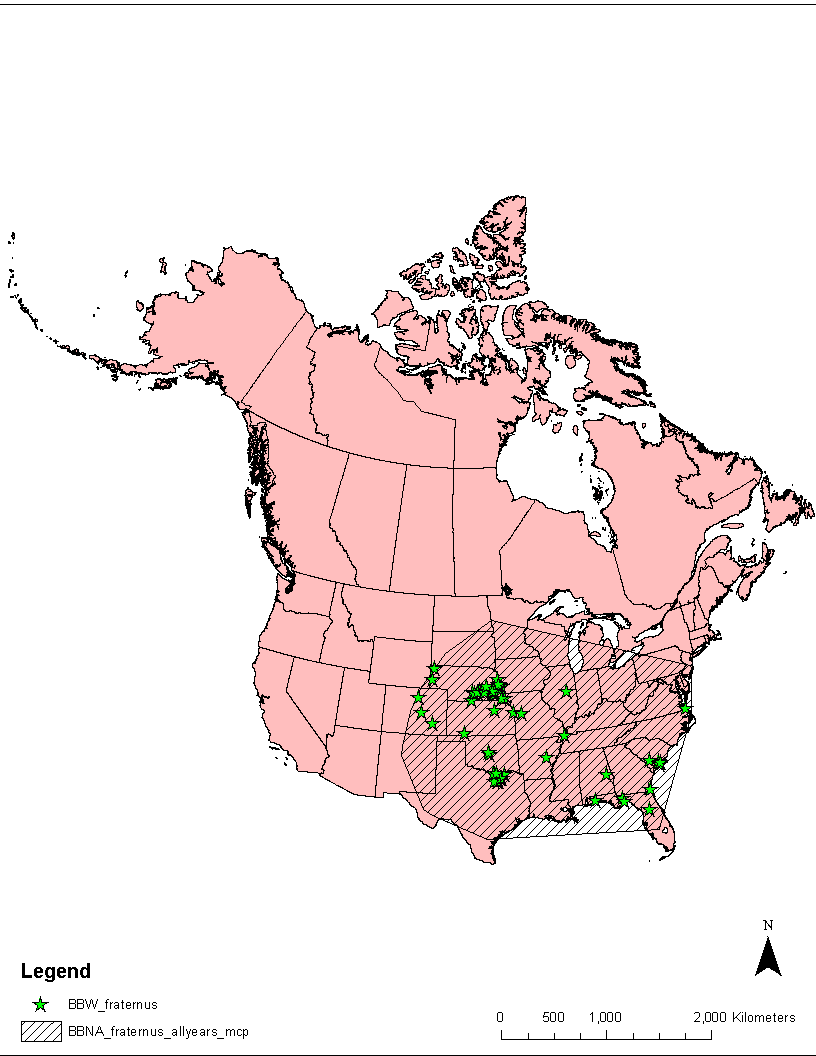

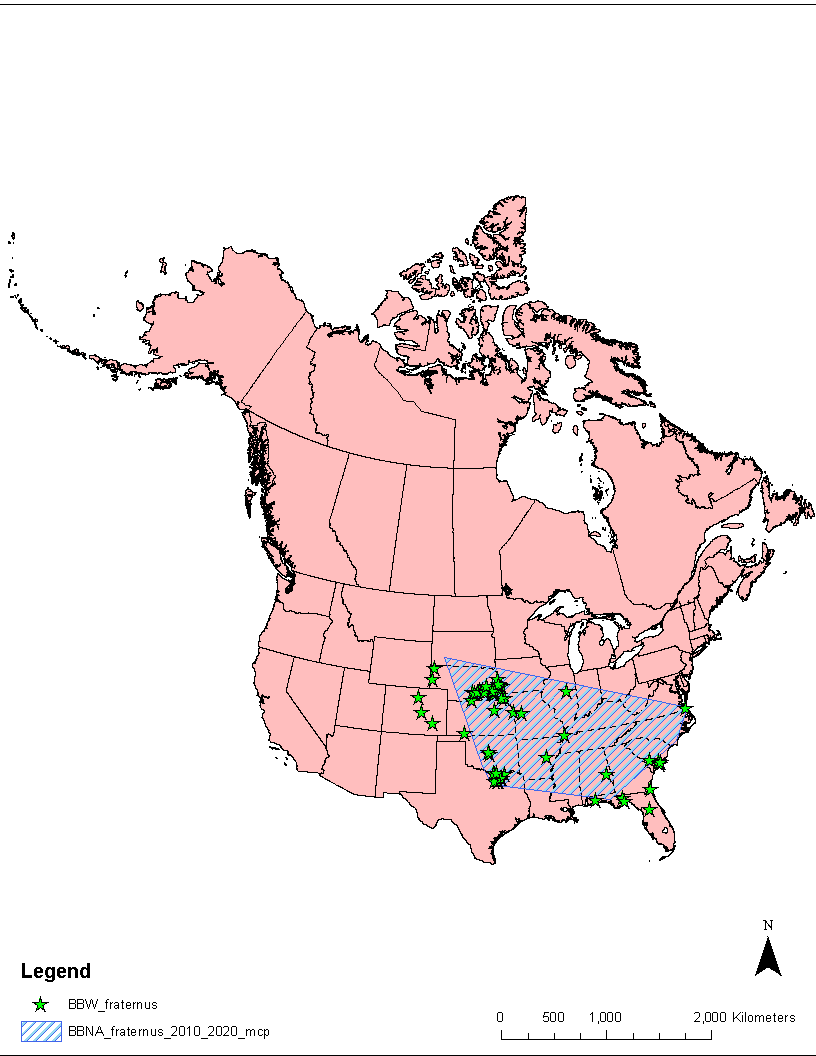
*

q) *Bombus frigidus*

*
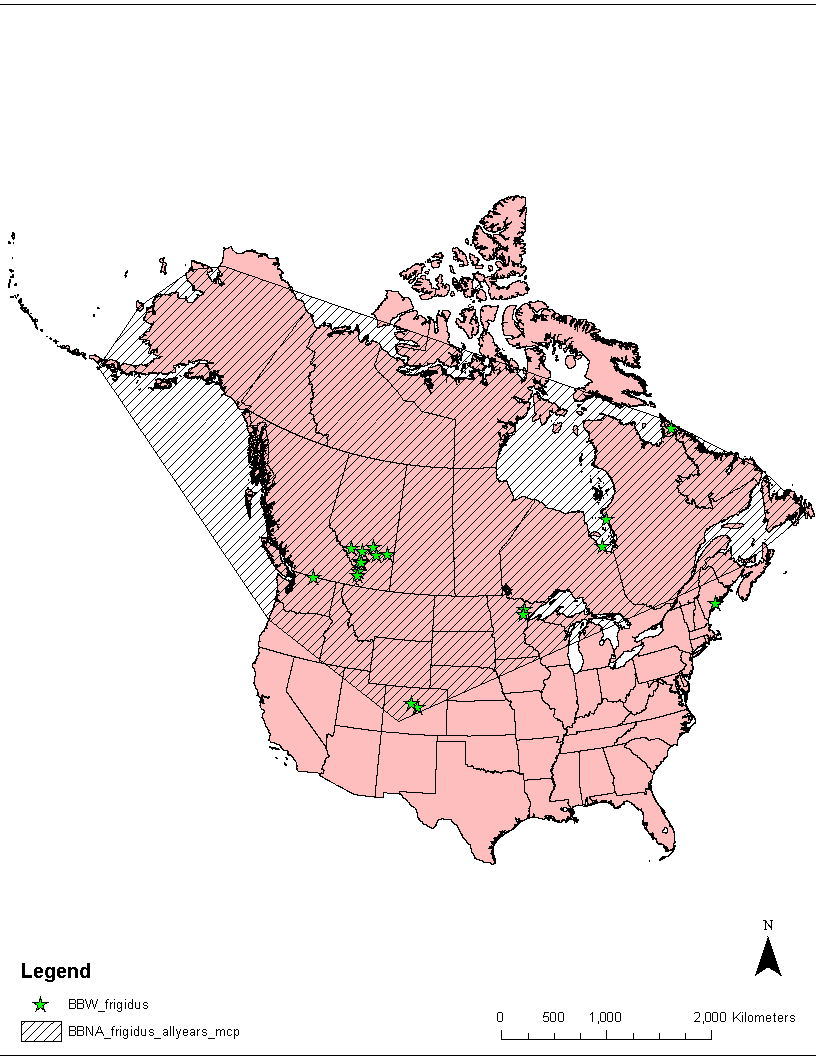

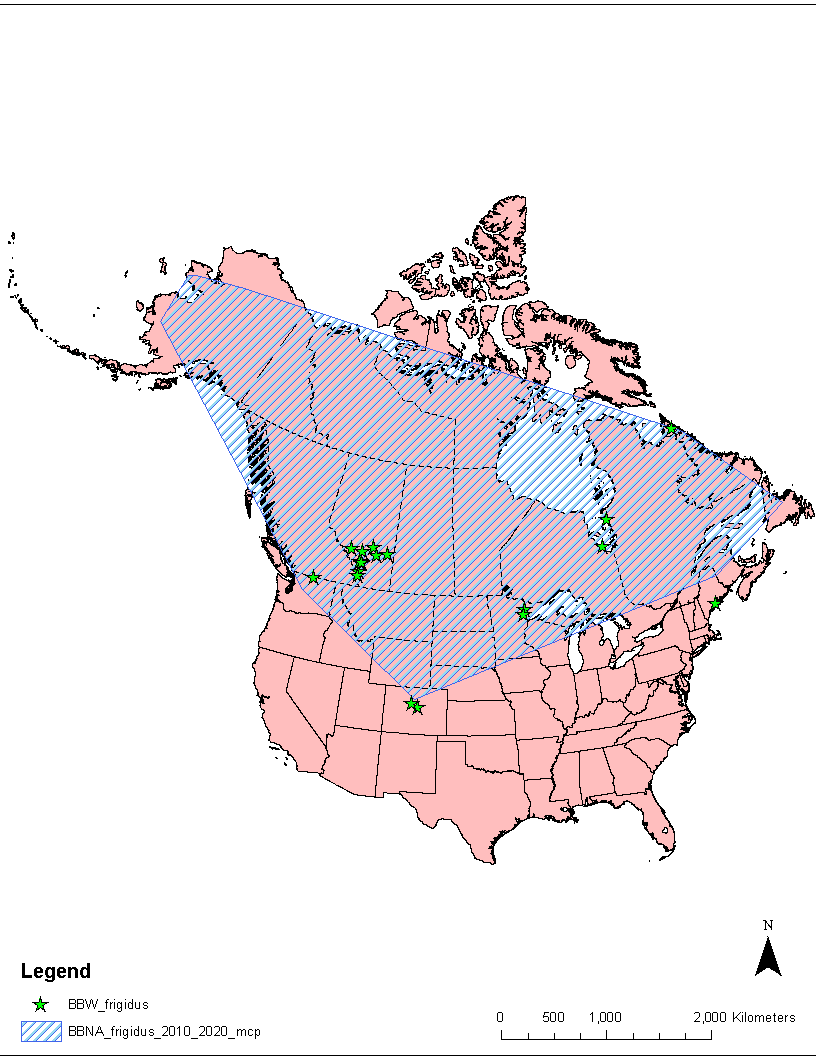
*

r) *Bombus griseocollis*

*
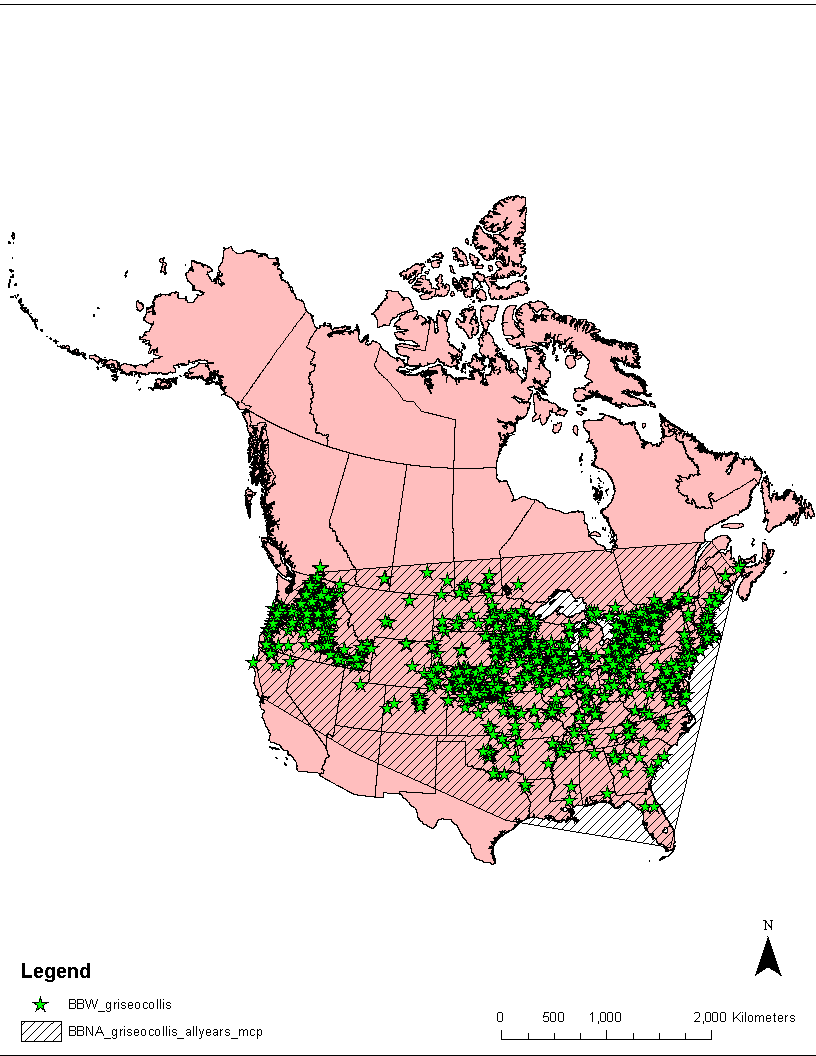

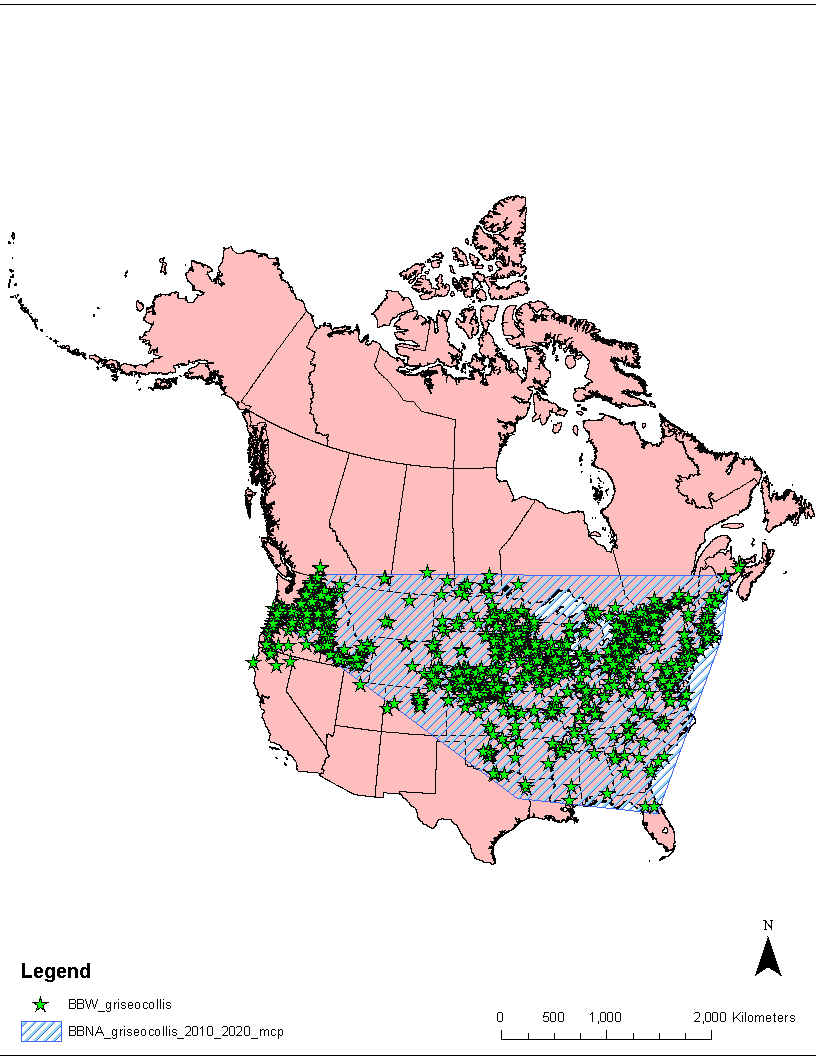
*

s) *Bombus huntii*

*
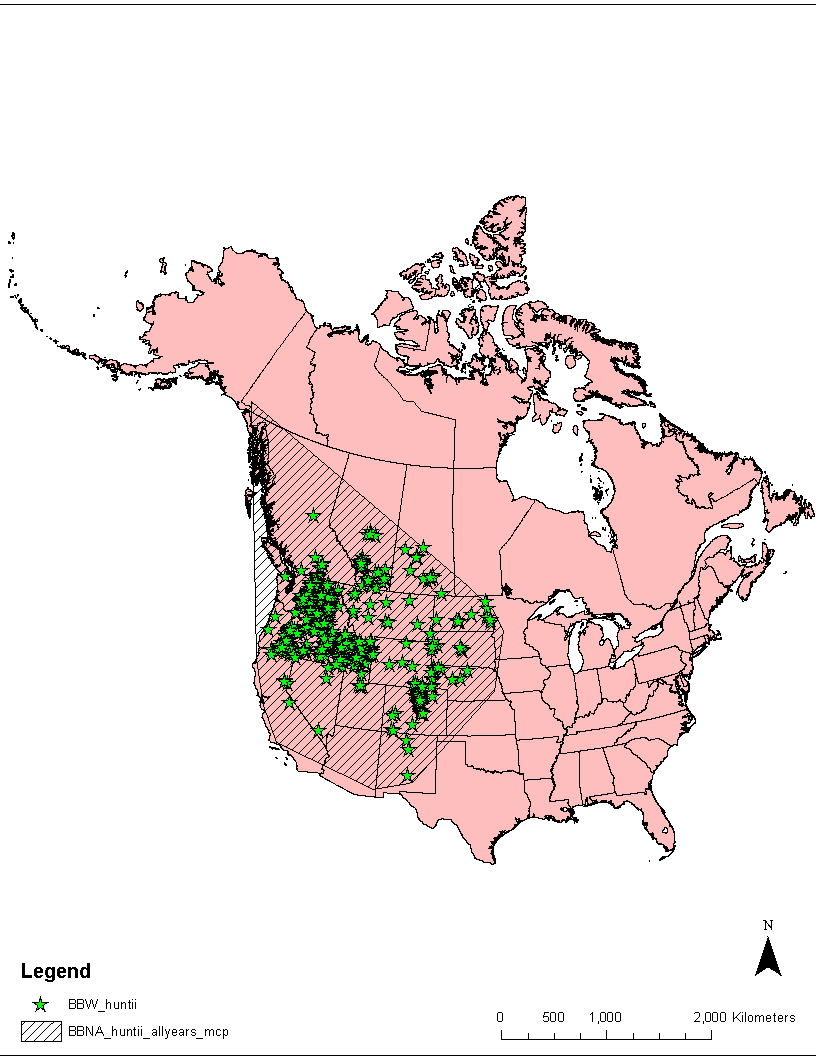

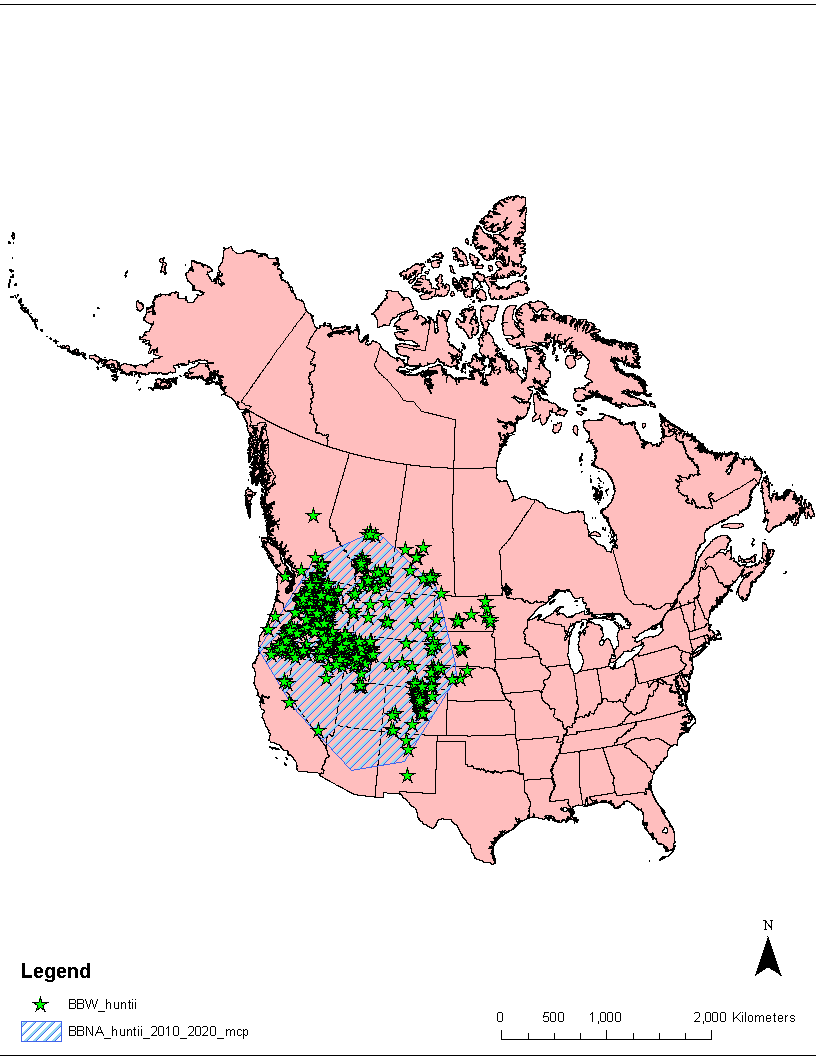
*

t) *Bombus impatiens*

*
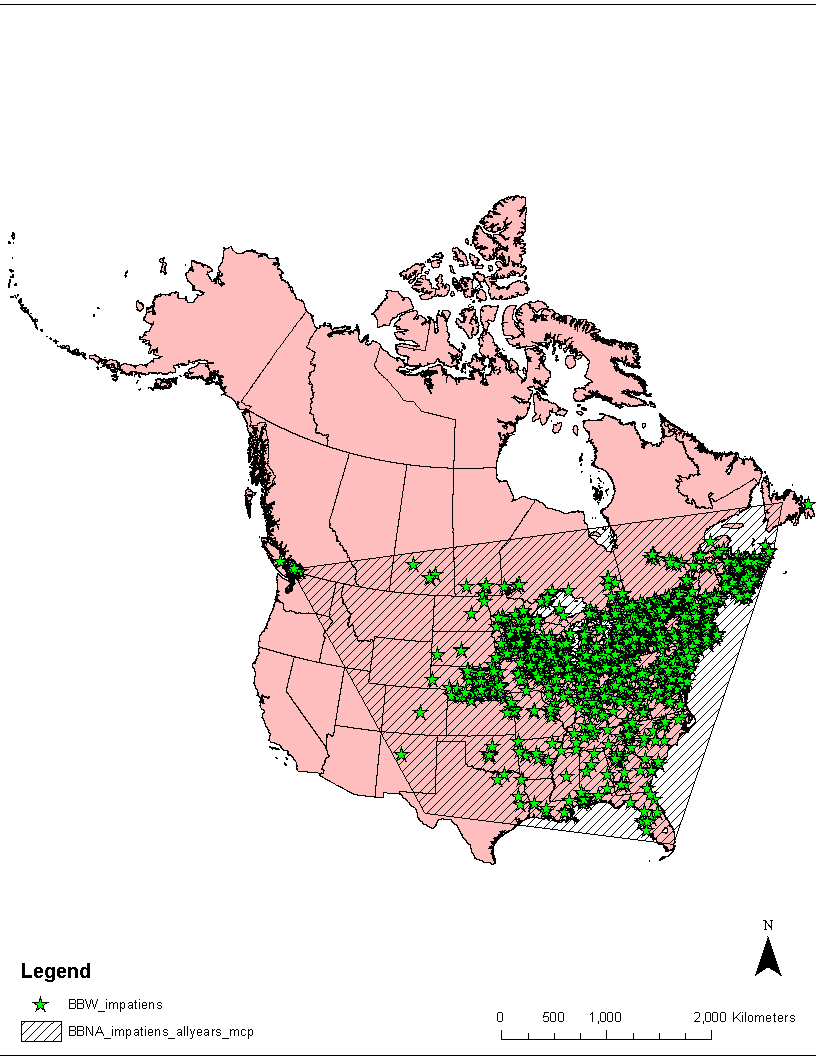

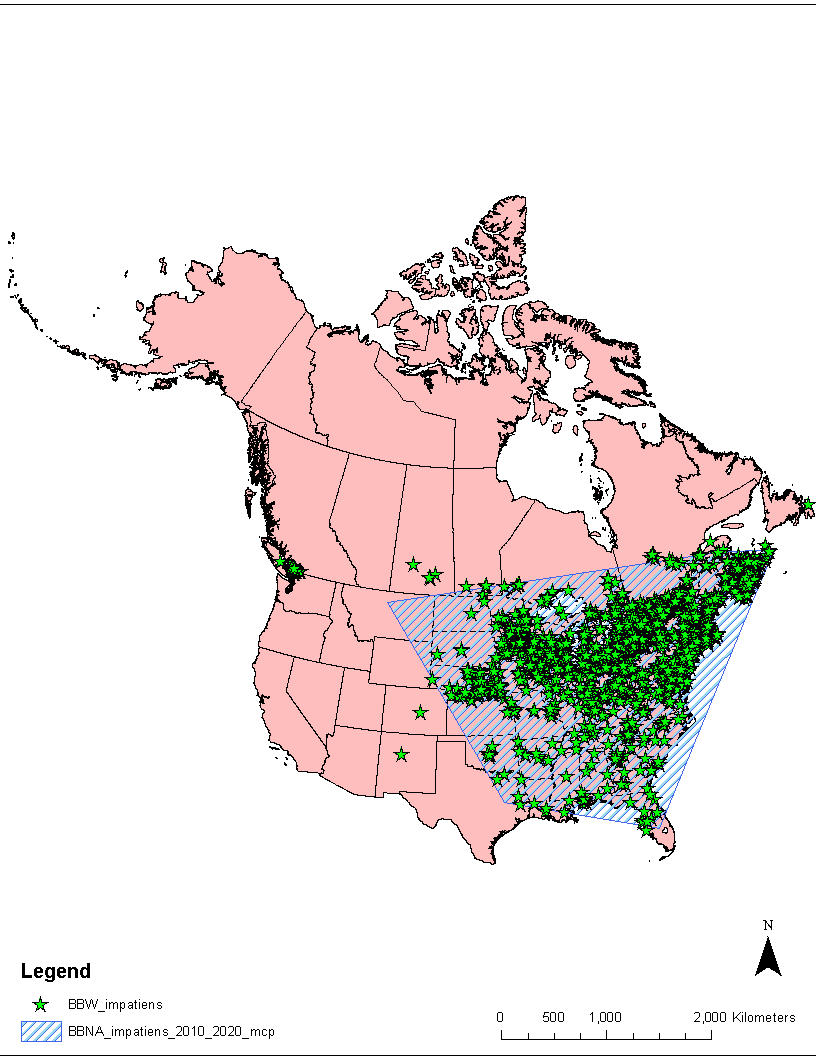
*

u) *Bombus insularis*

*
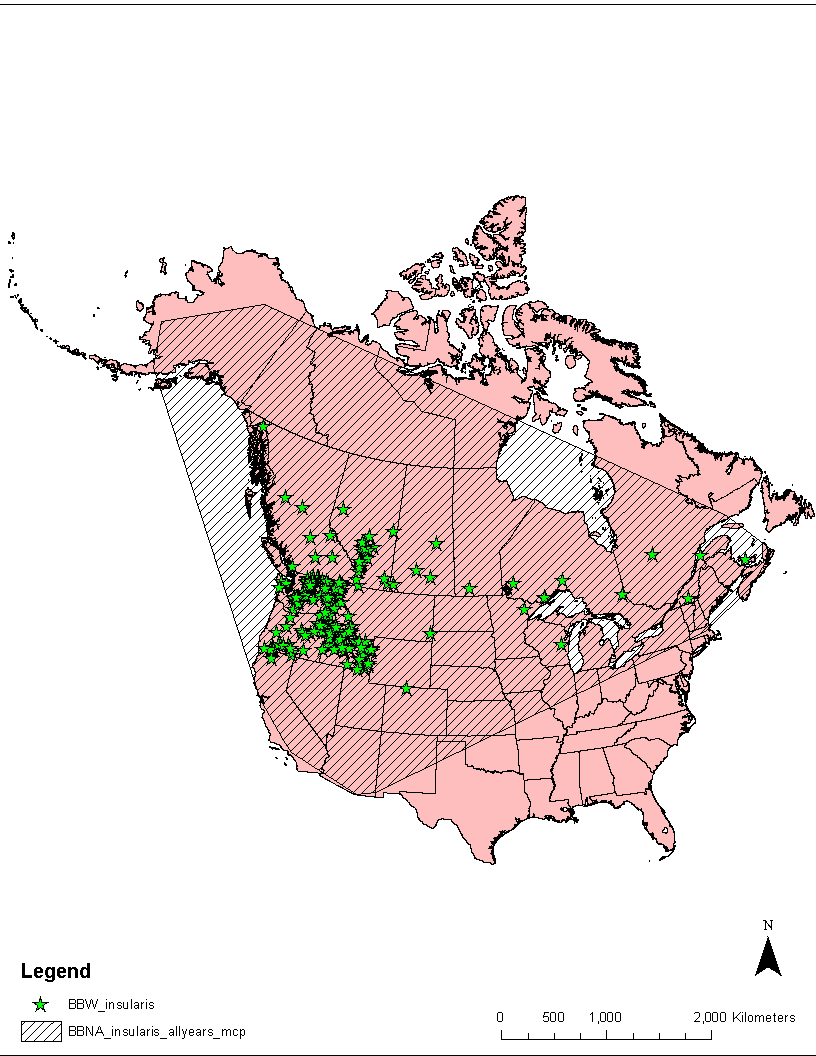

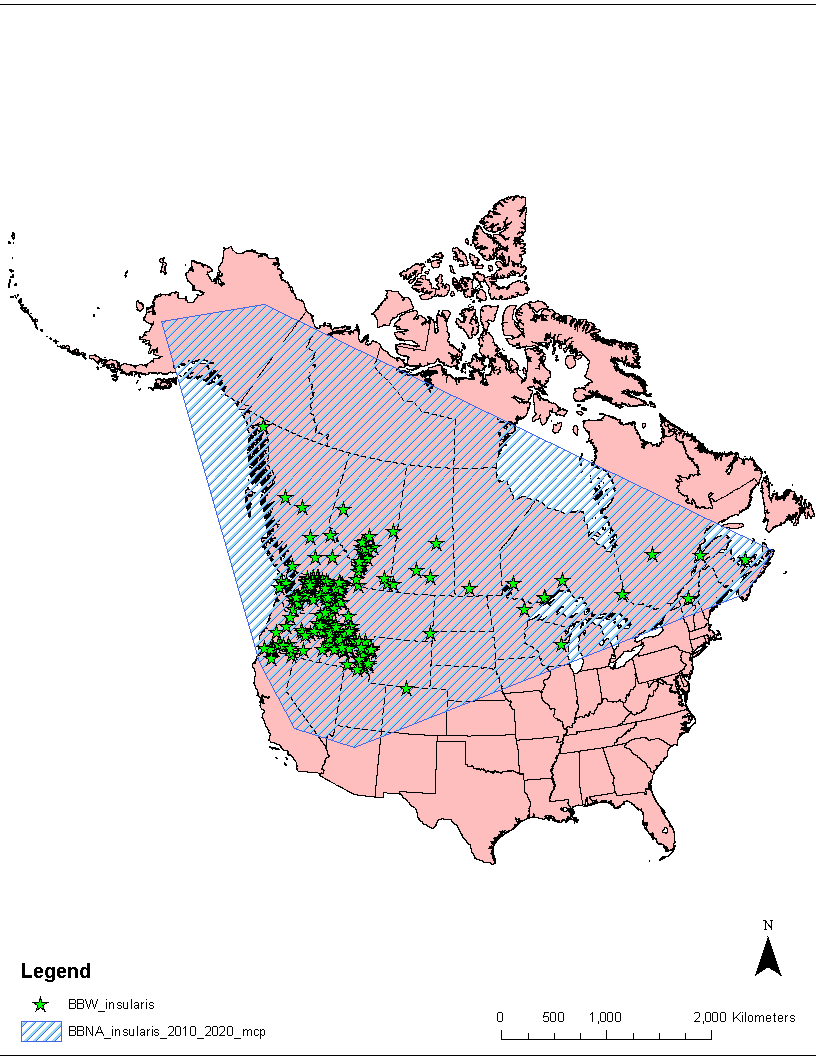
*

v) *Bombus jonellus*

*
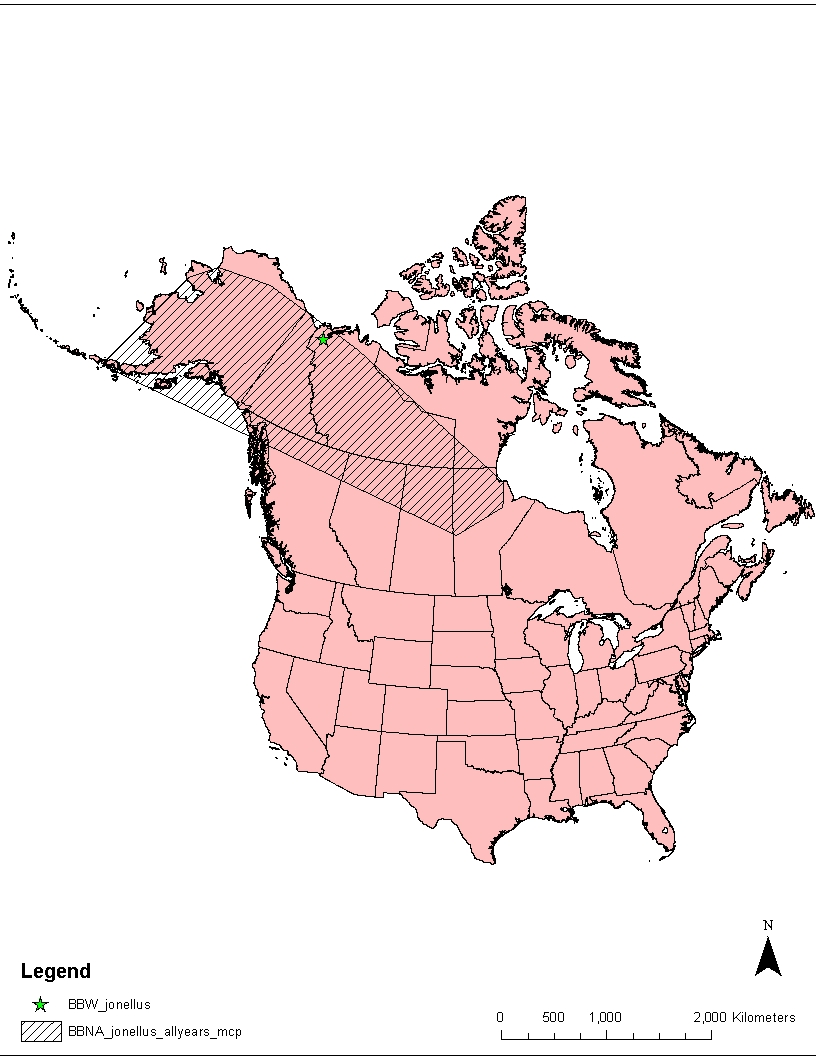

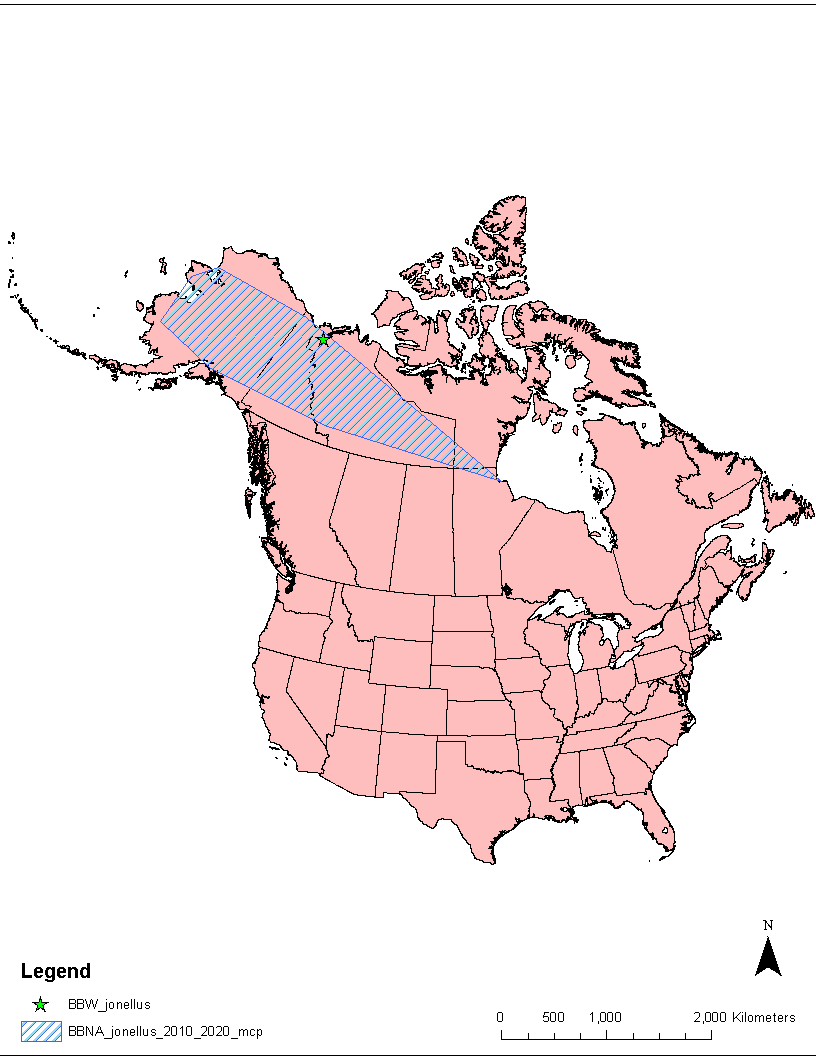
*

w) *Bombus kirbiellus*

*
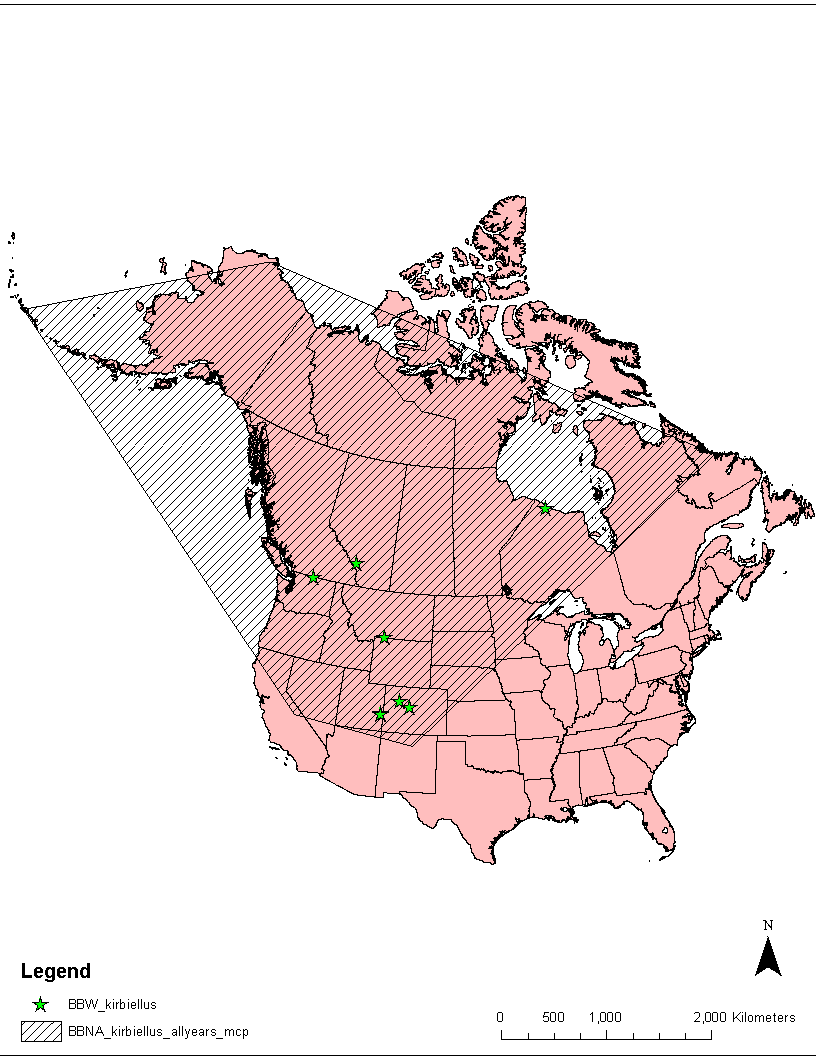

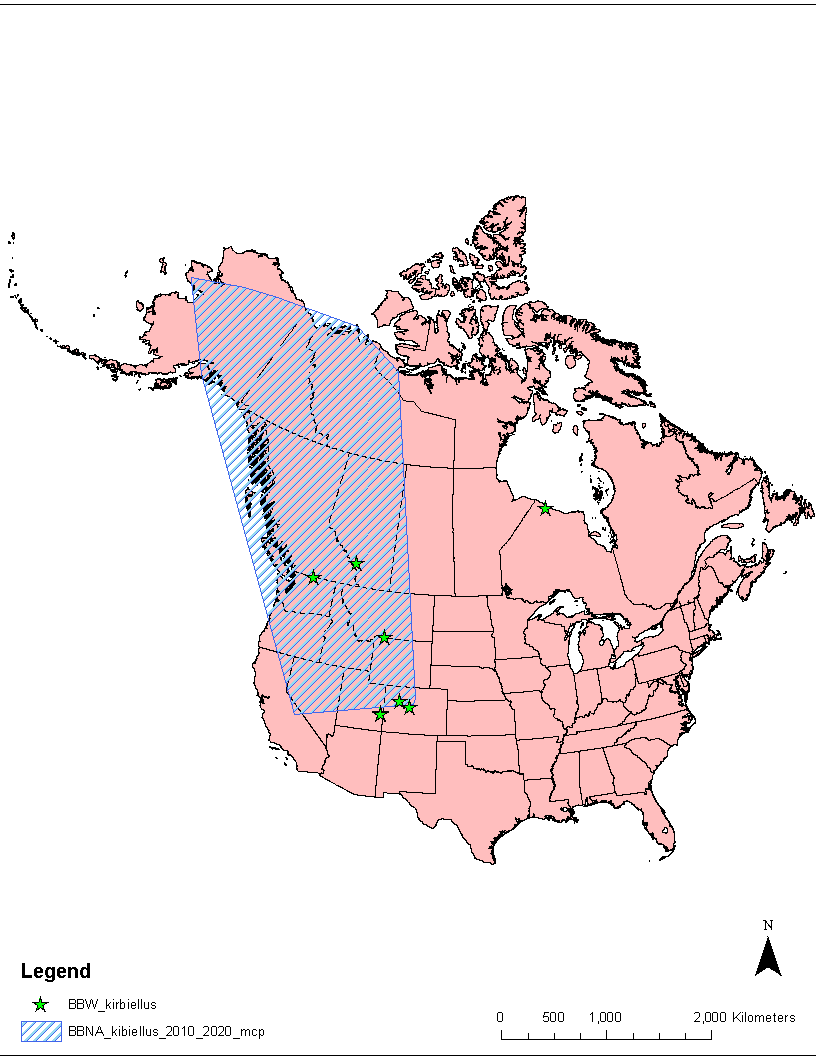
*

x) *Bombus melanopygus*

*
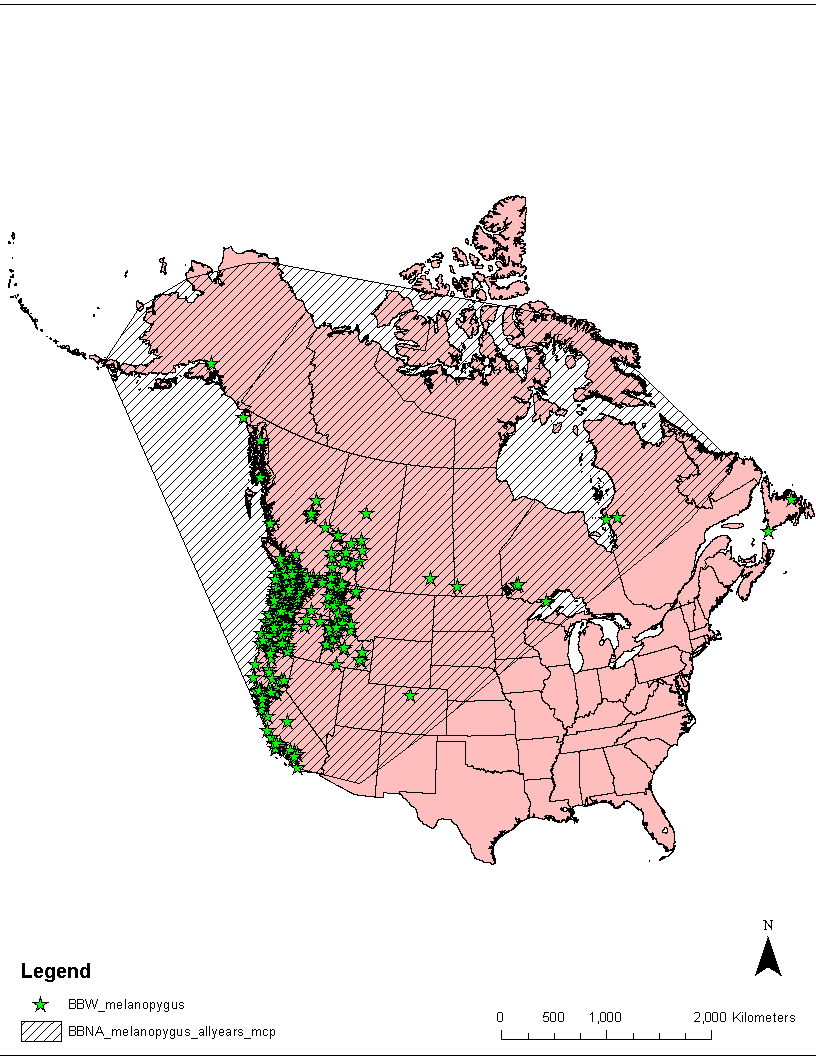

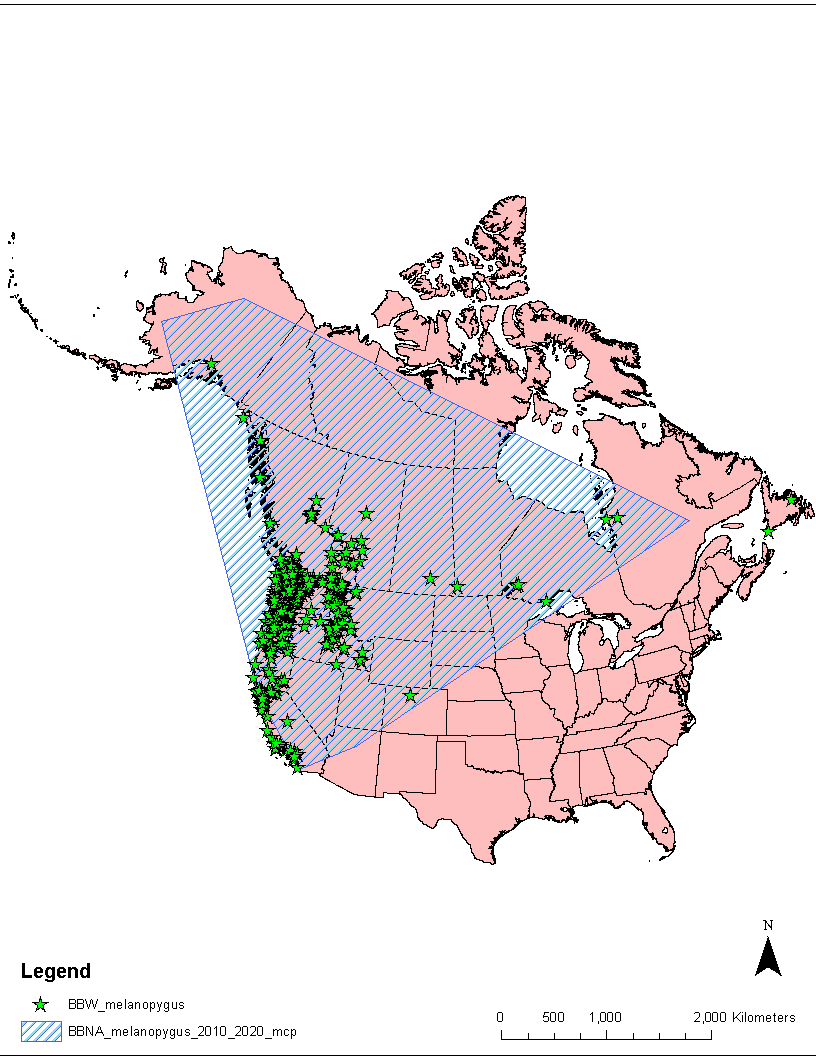
*

y) *Bombus mixtus*

*
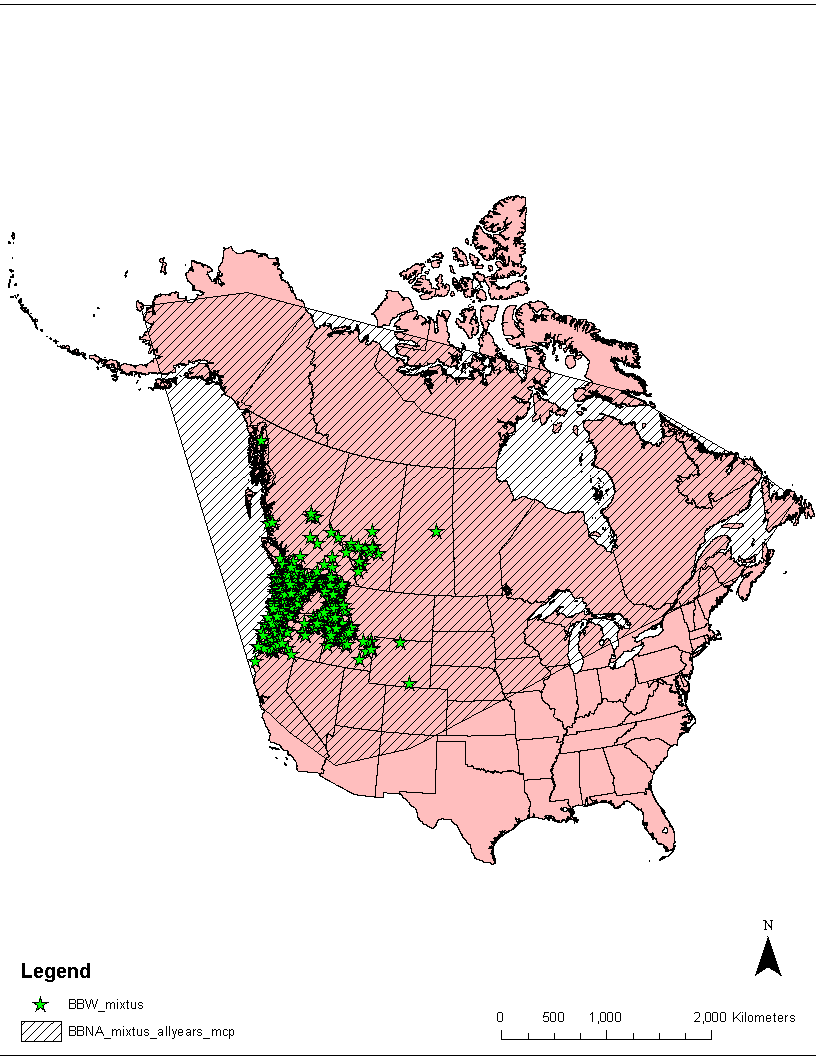

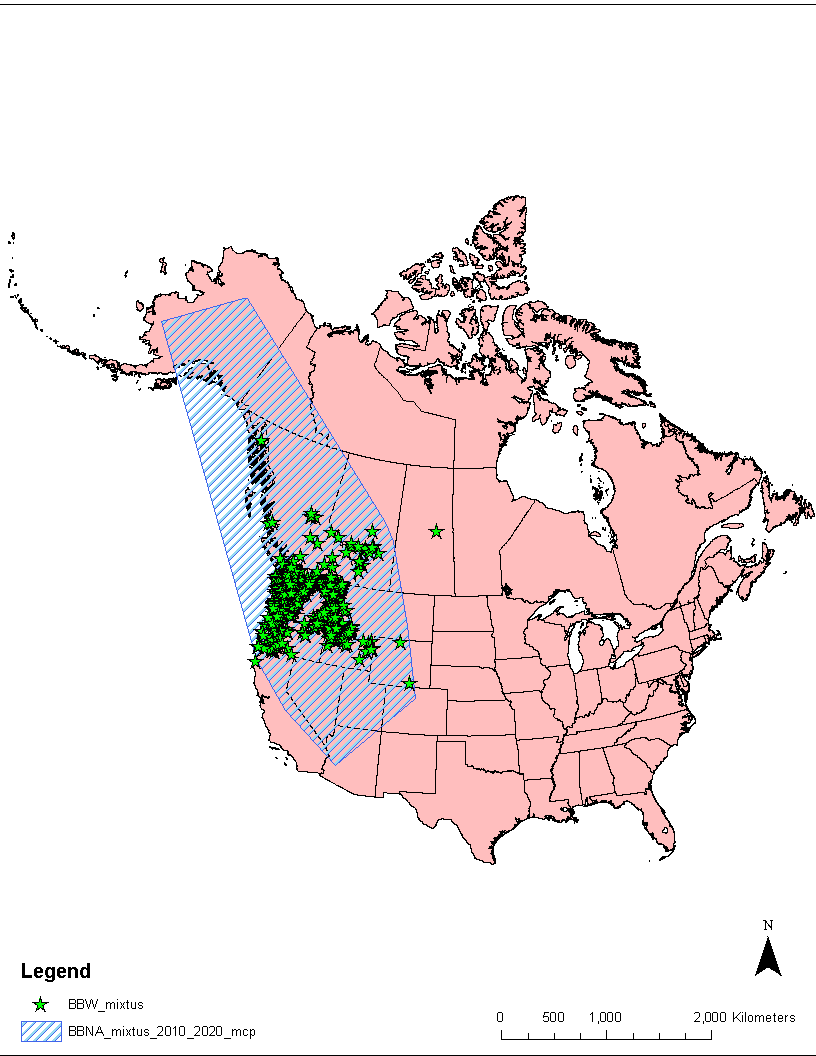
*

z) *Bombus morrisoni*

*
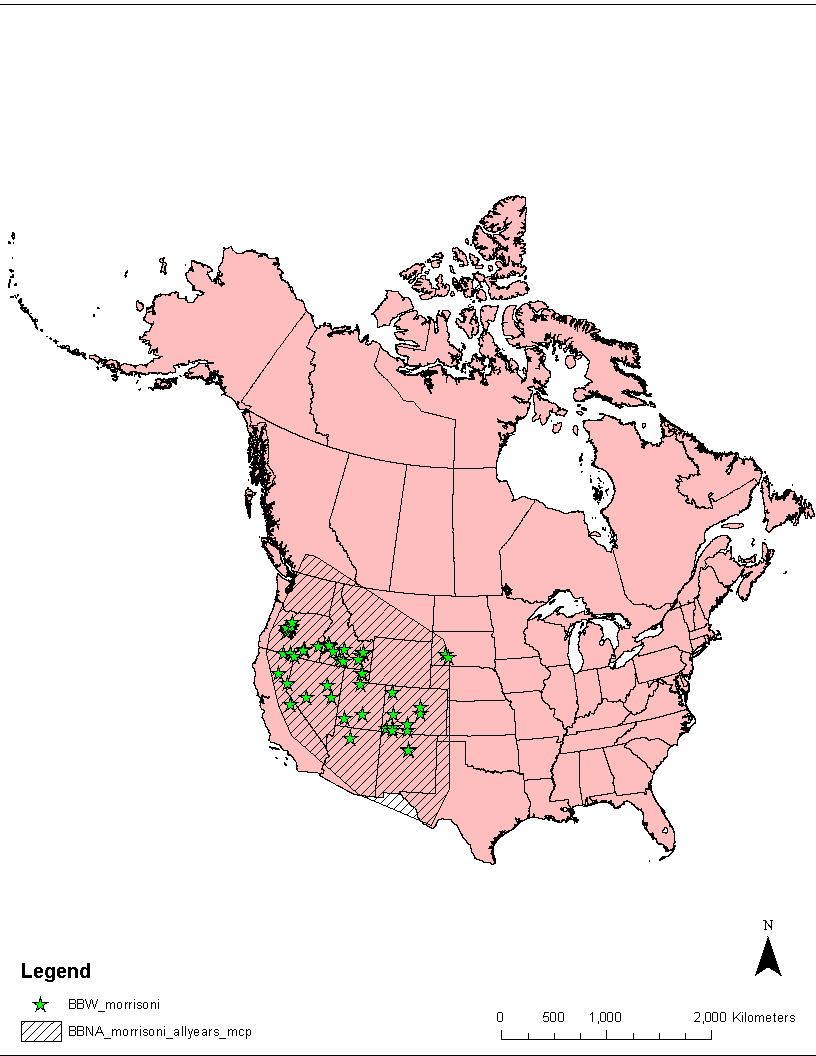

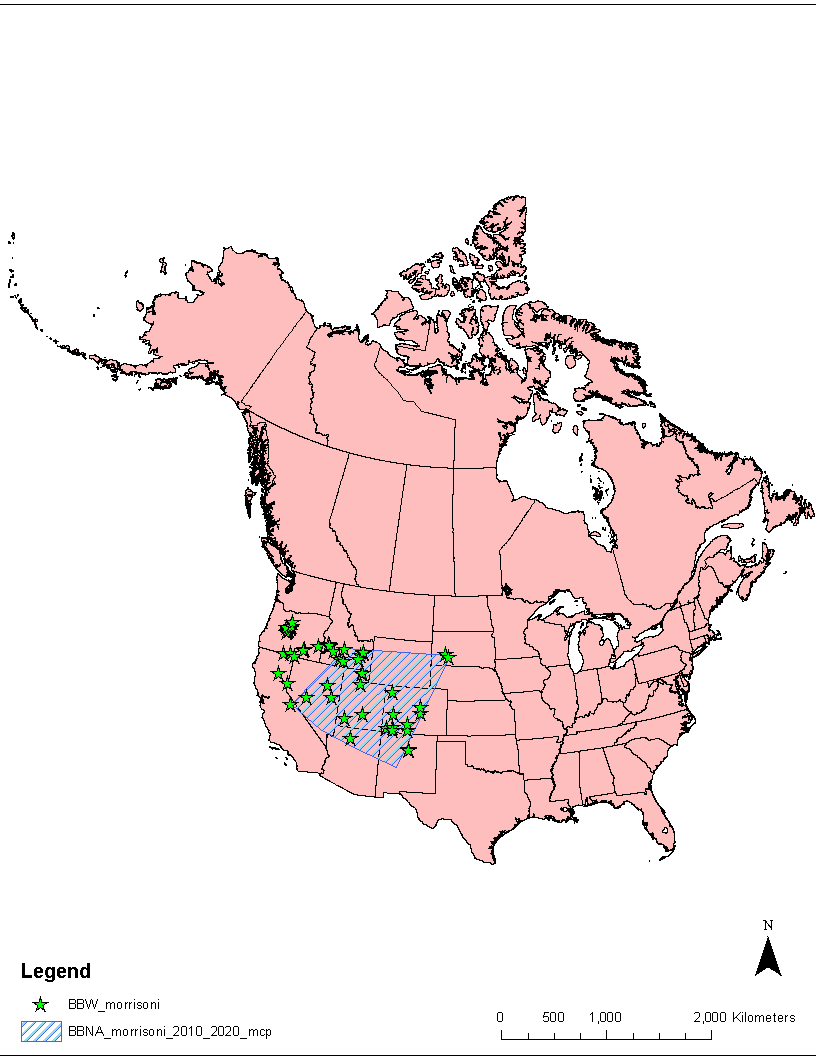
*

aa) *Bombus nevadensis*

*
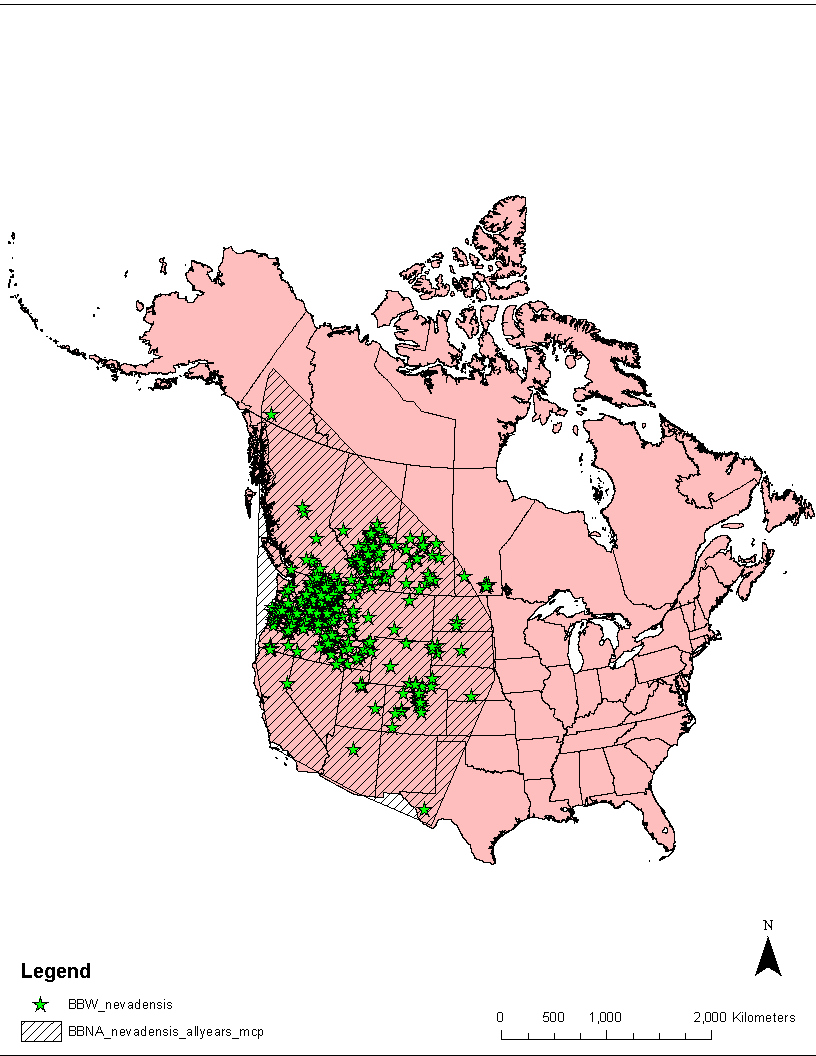

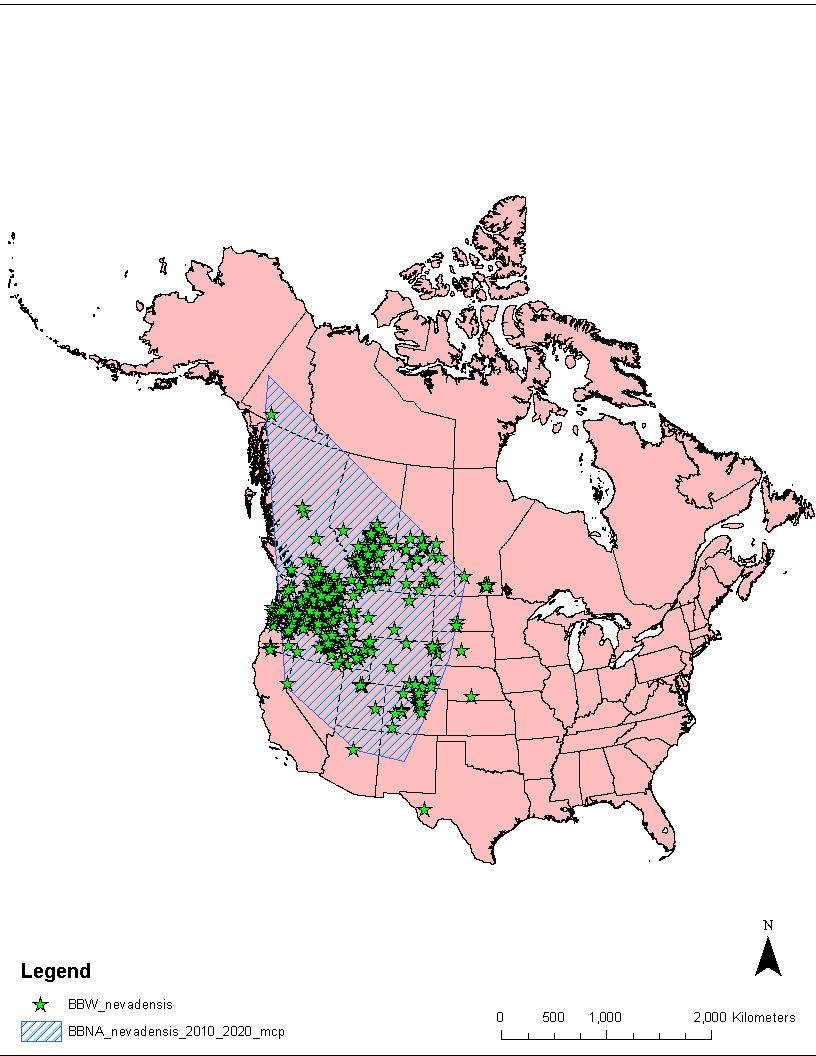
*

bb) *Bombus occidentalis*

*
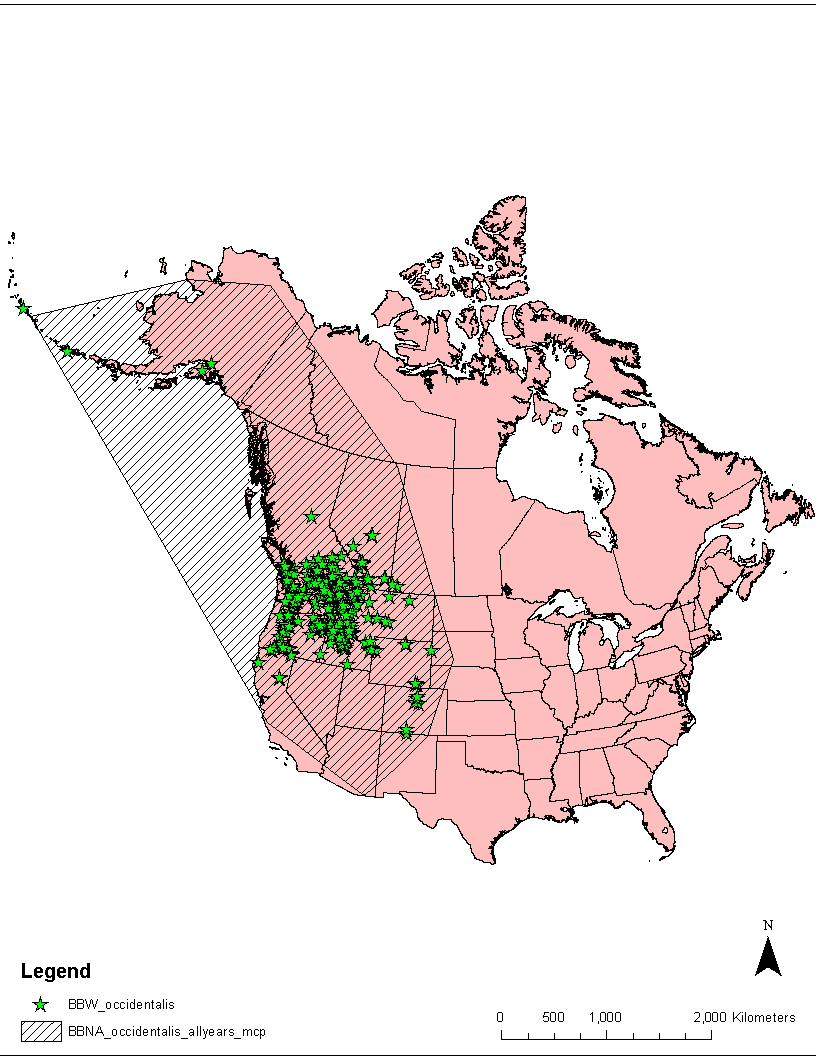

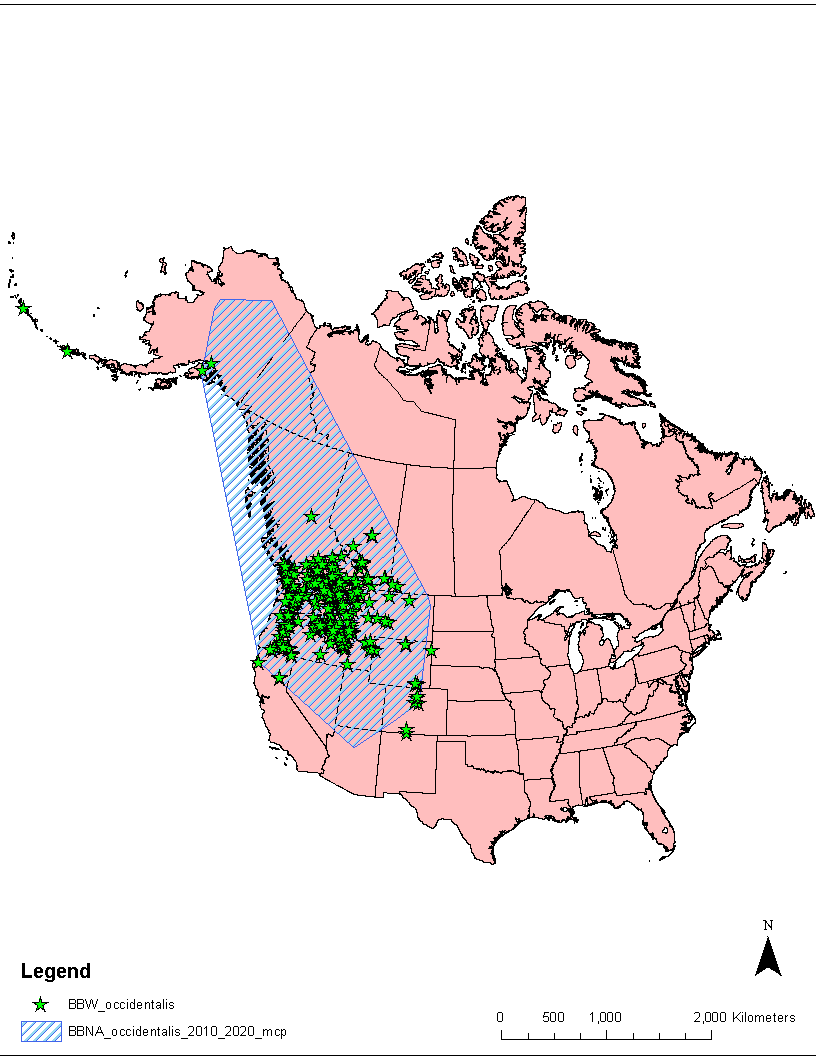
*

cc) *Bombus pensylvanicus*

*
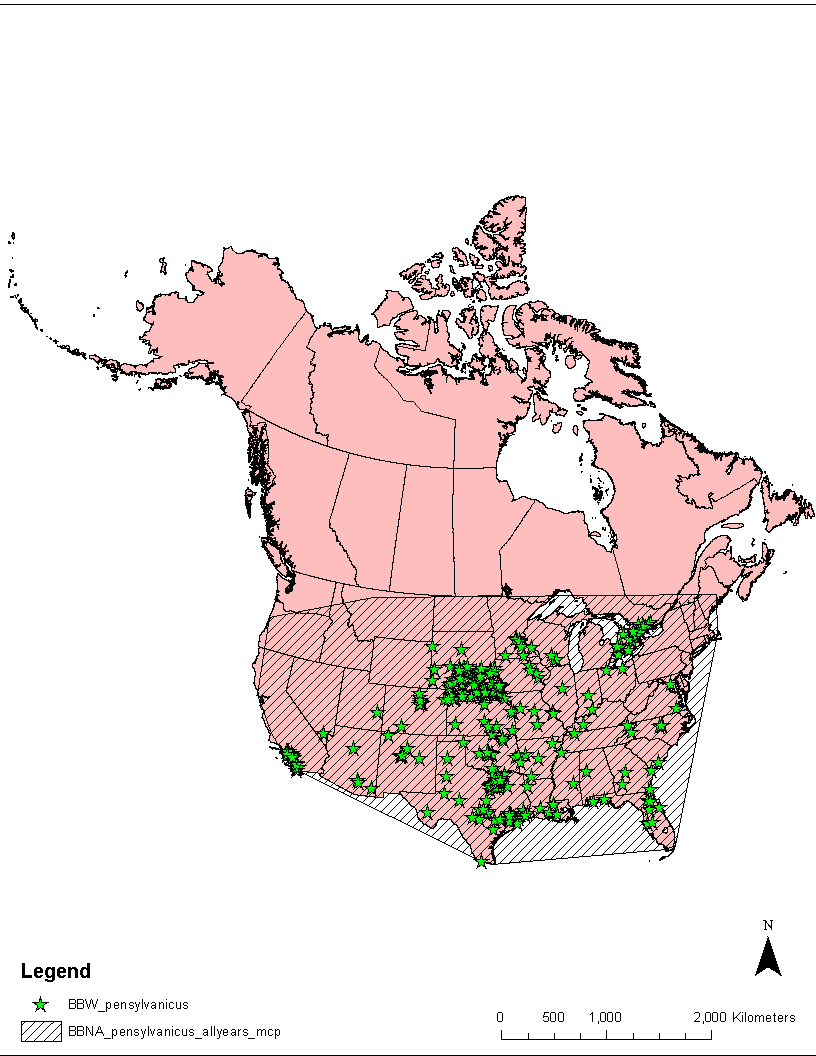

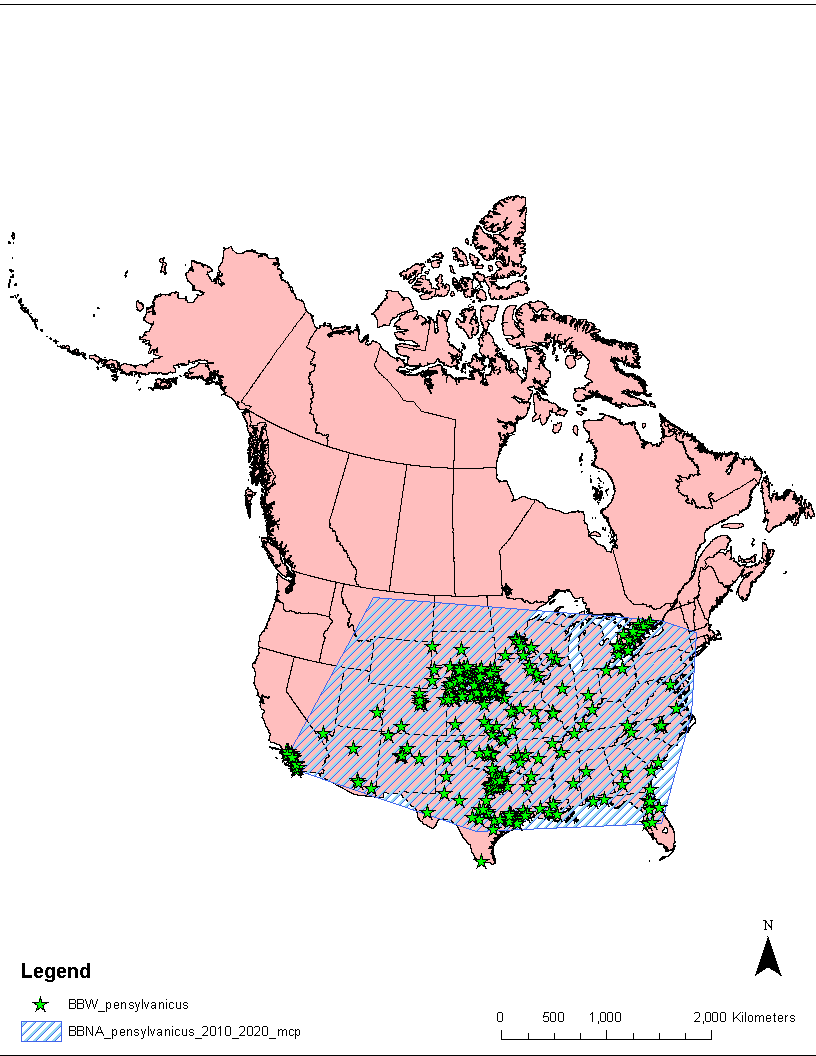
*

dd) *Bombus perplexus*

*
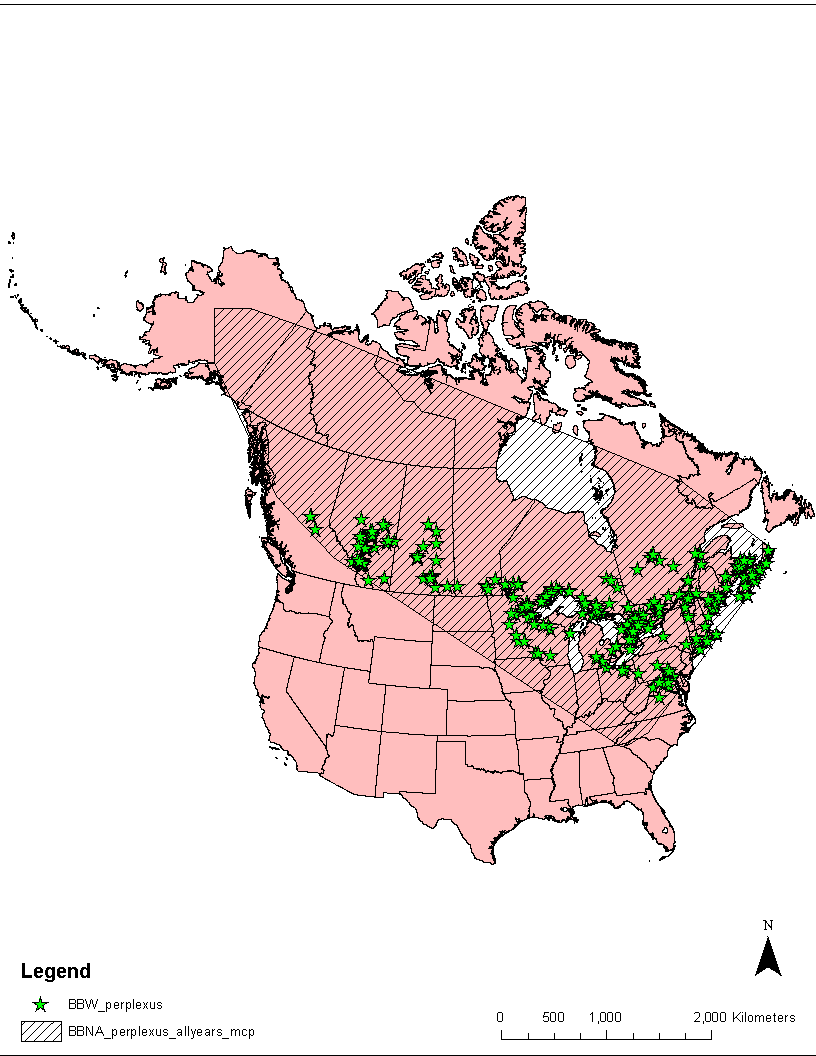

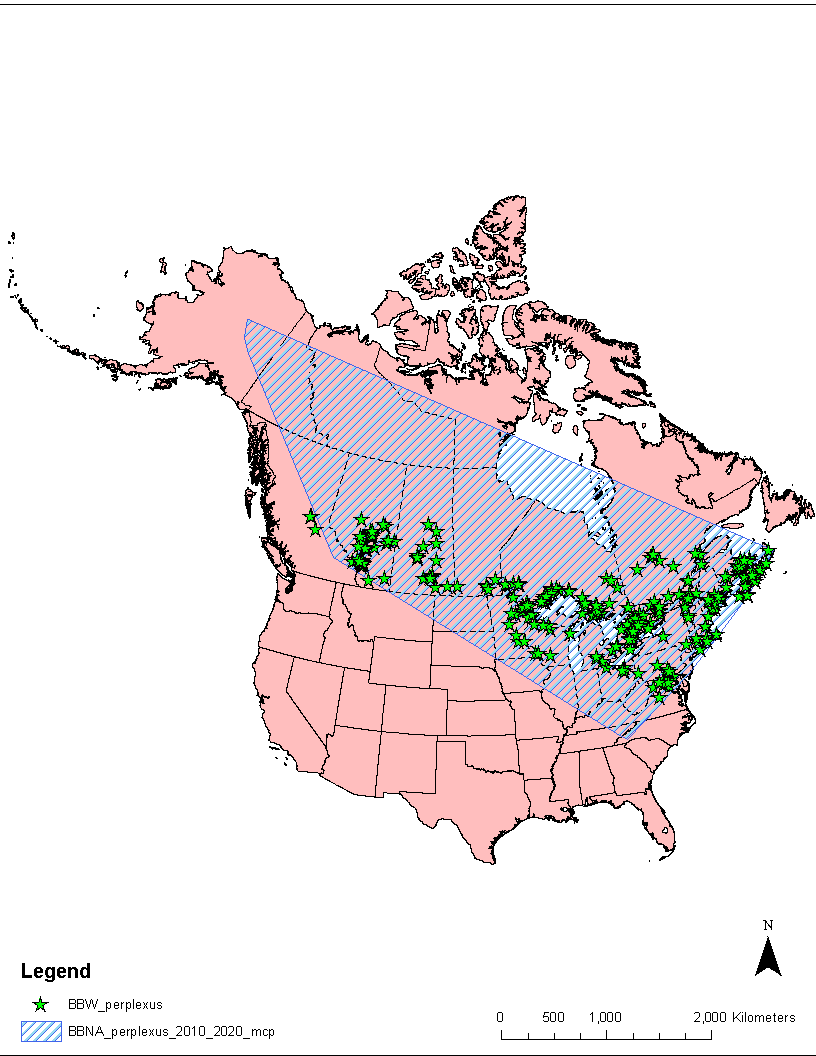
*

ee) *Bombus polaris*

*
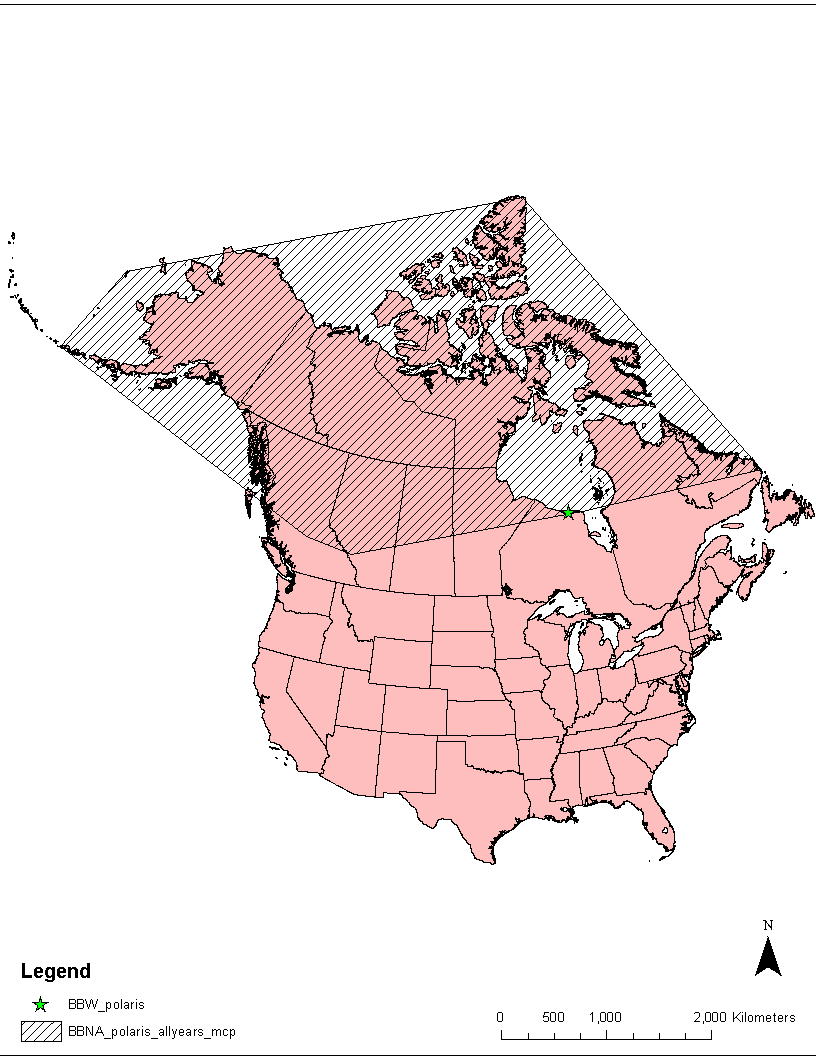

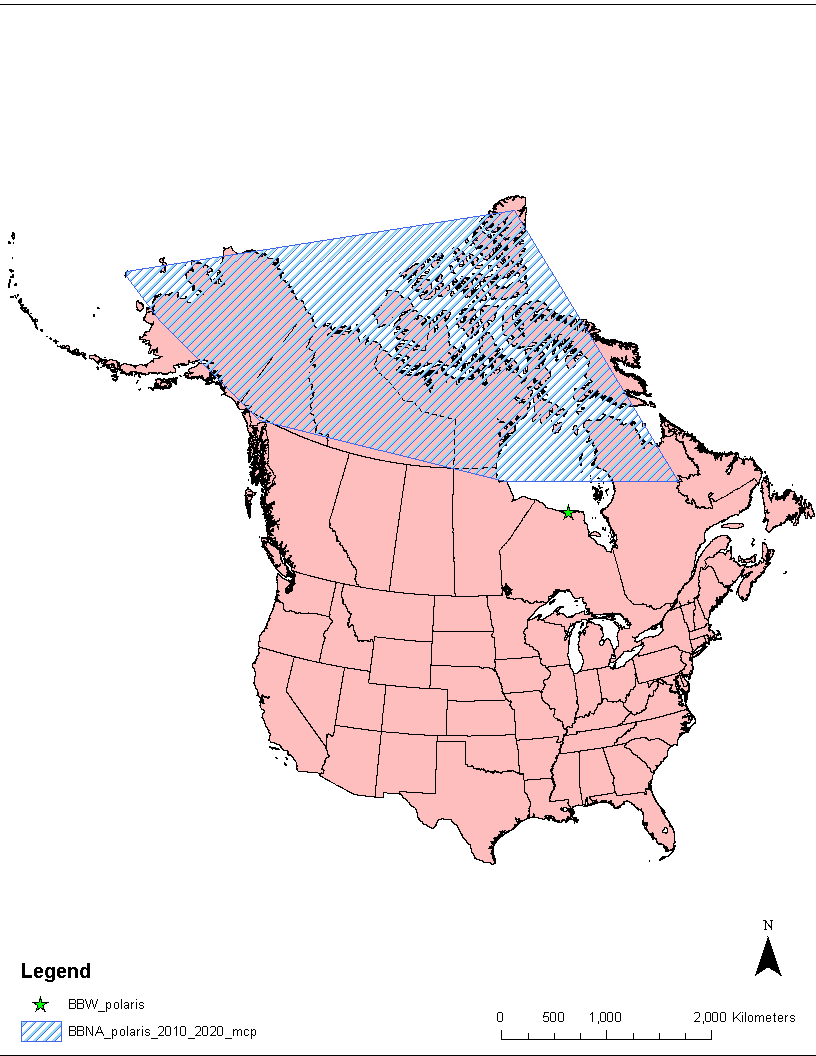
*

ff) *Bombus rufocinctus*

*
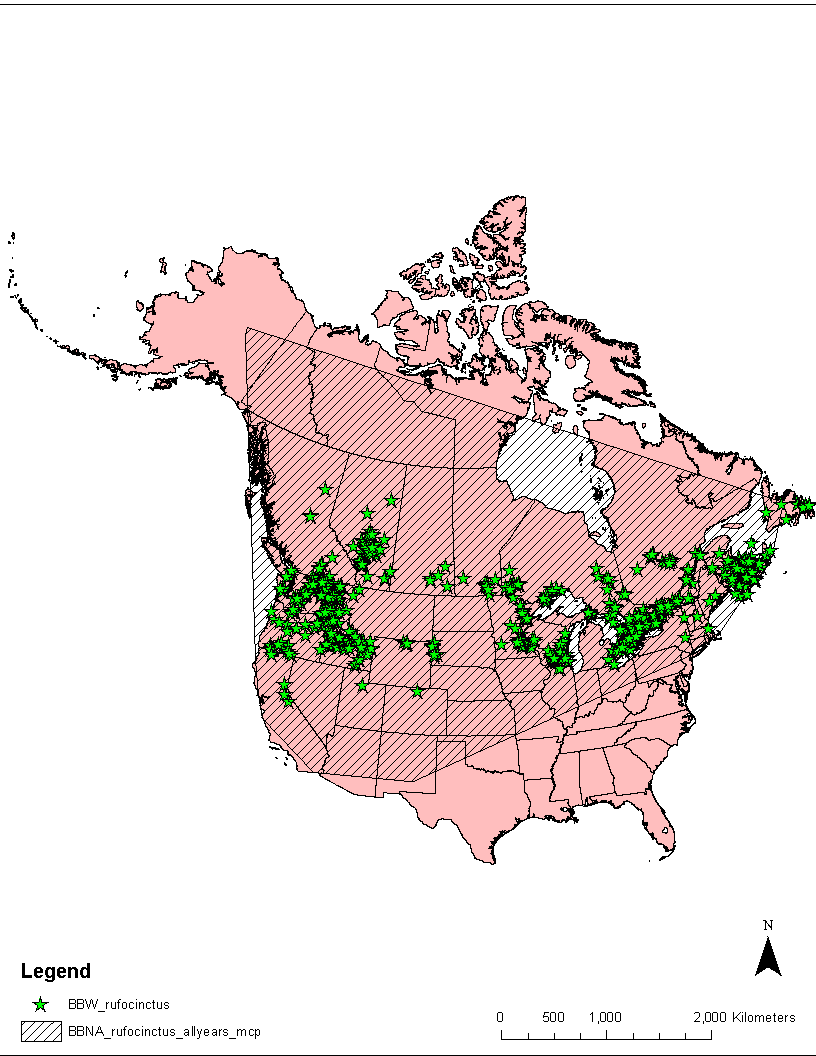

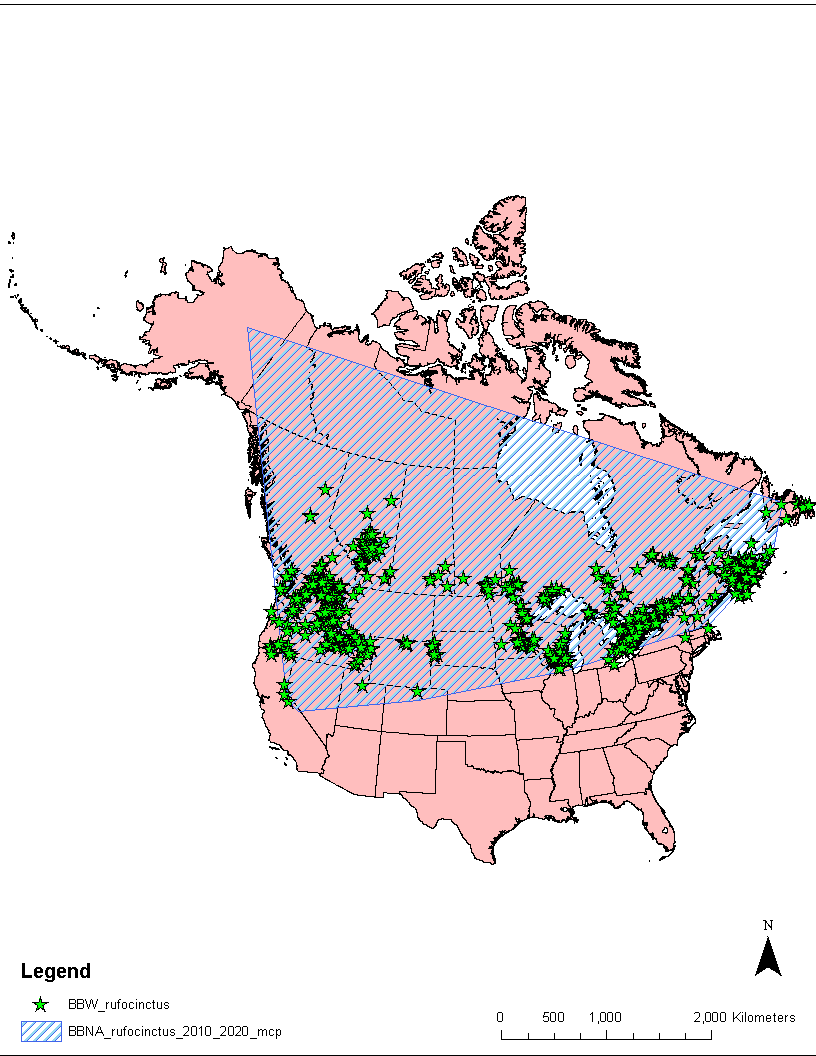
*

gg) *Bombus sandersoni*

*
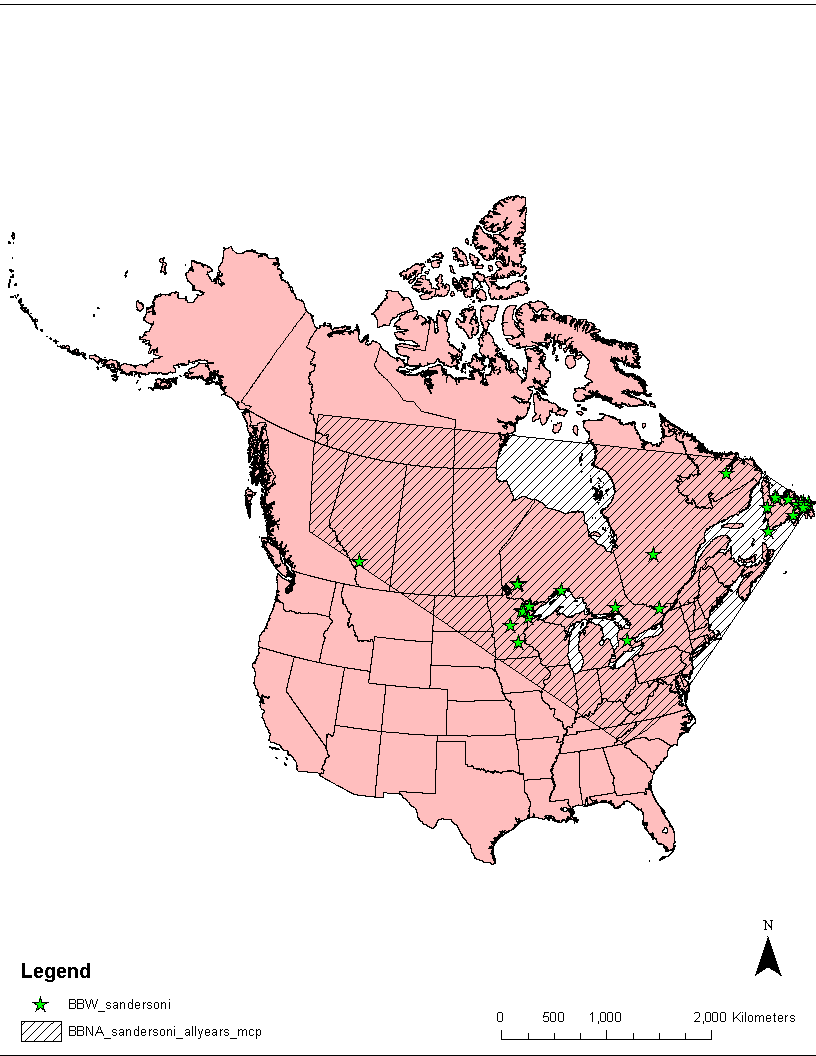

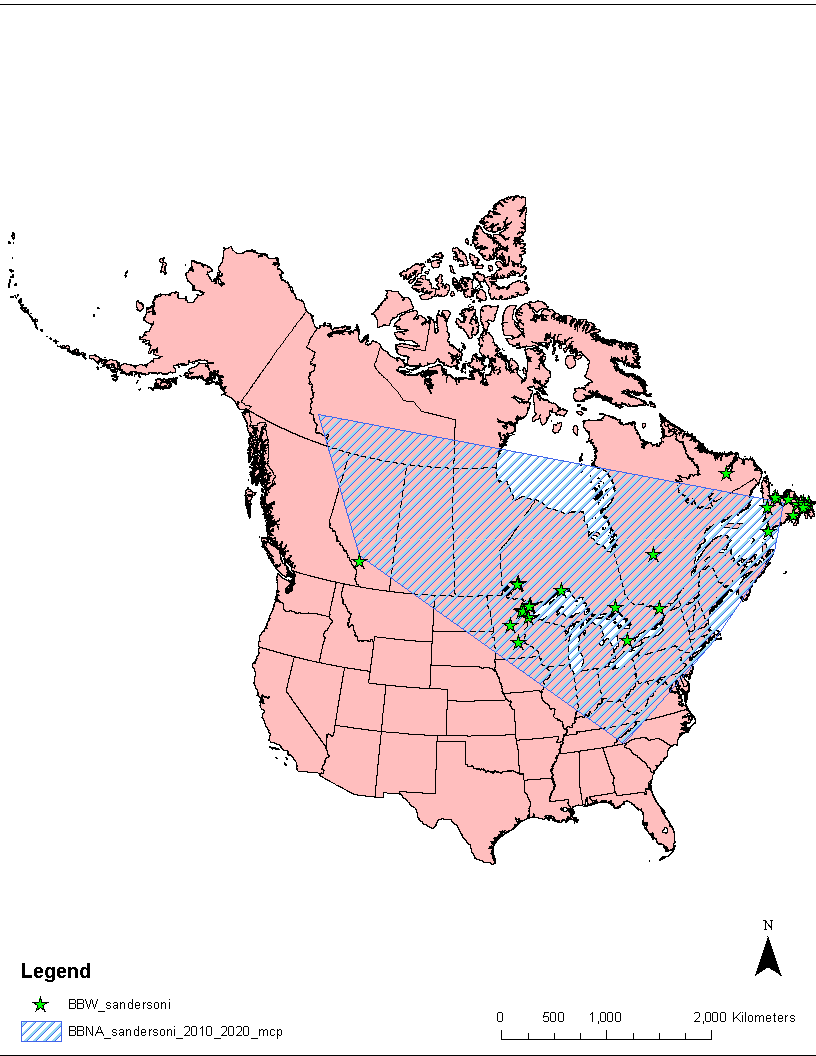
*

hh) *Bombus sitkensis*

*
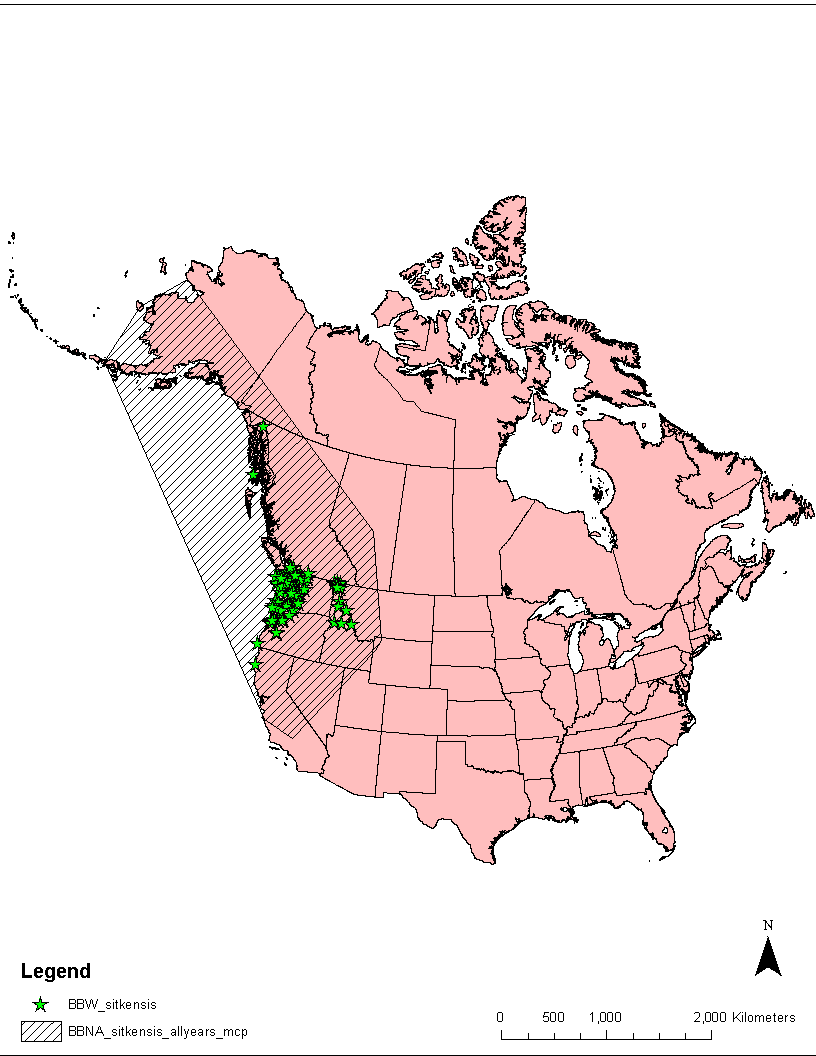

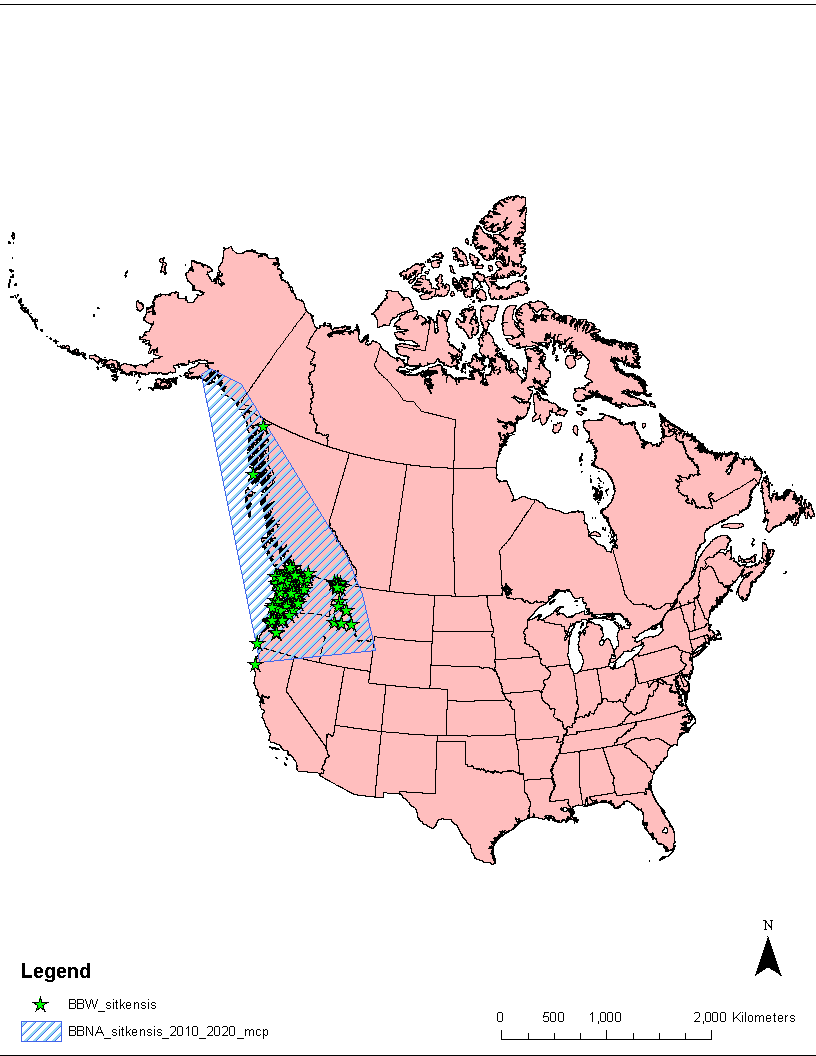
*

ii) *Bombus suckleyi*

*
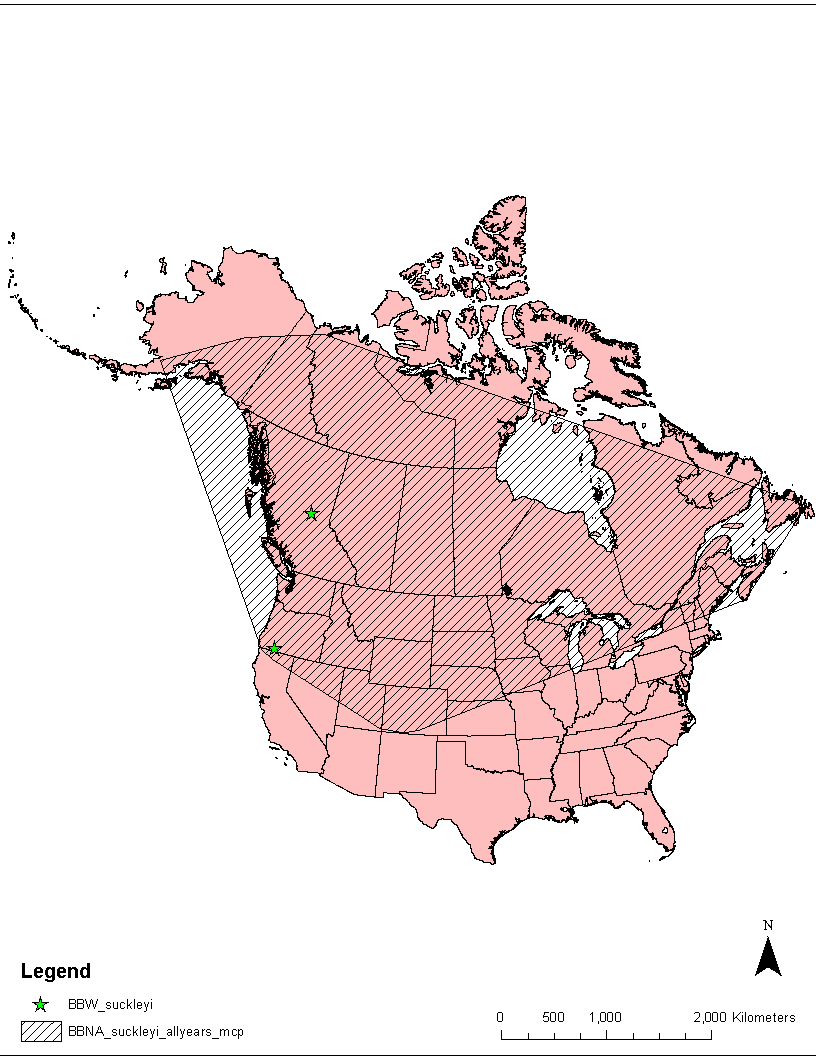

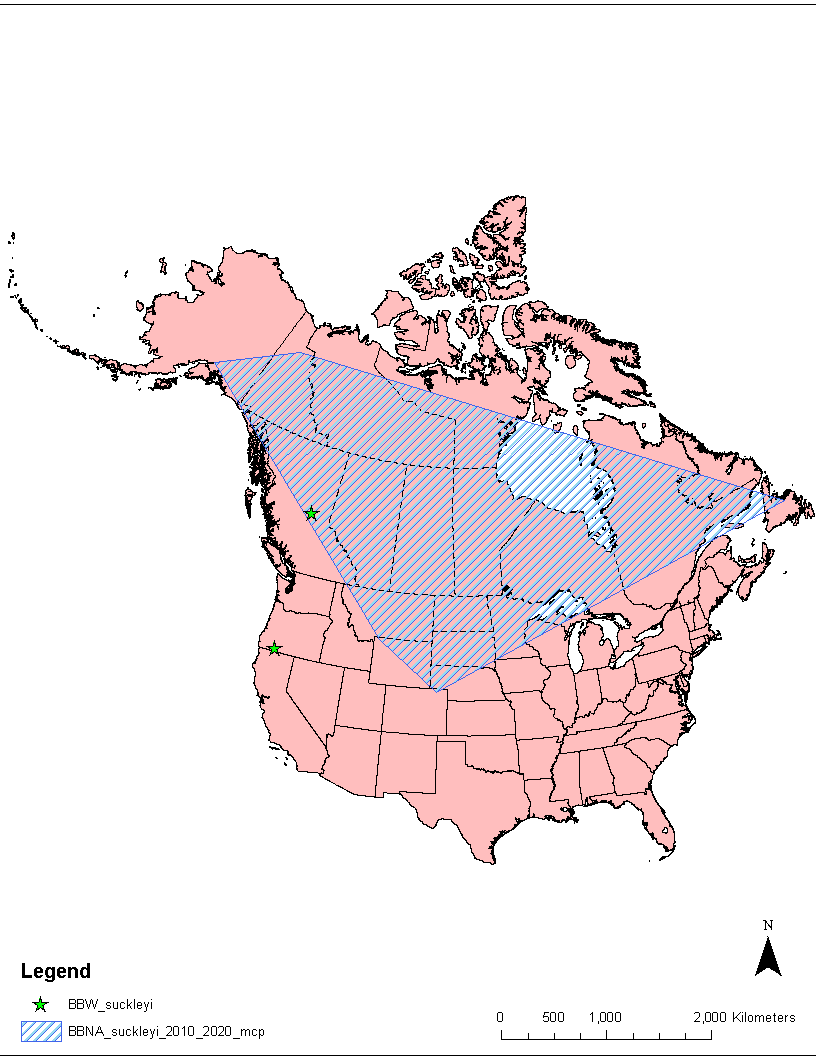
*

jj) *Bombus sylvicola*

*
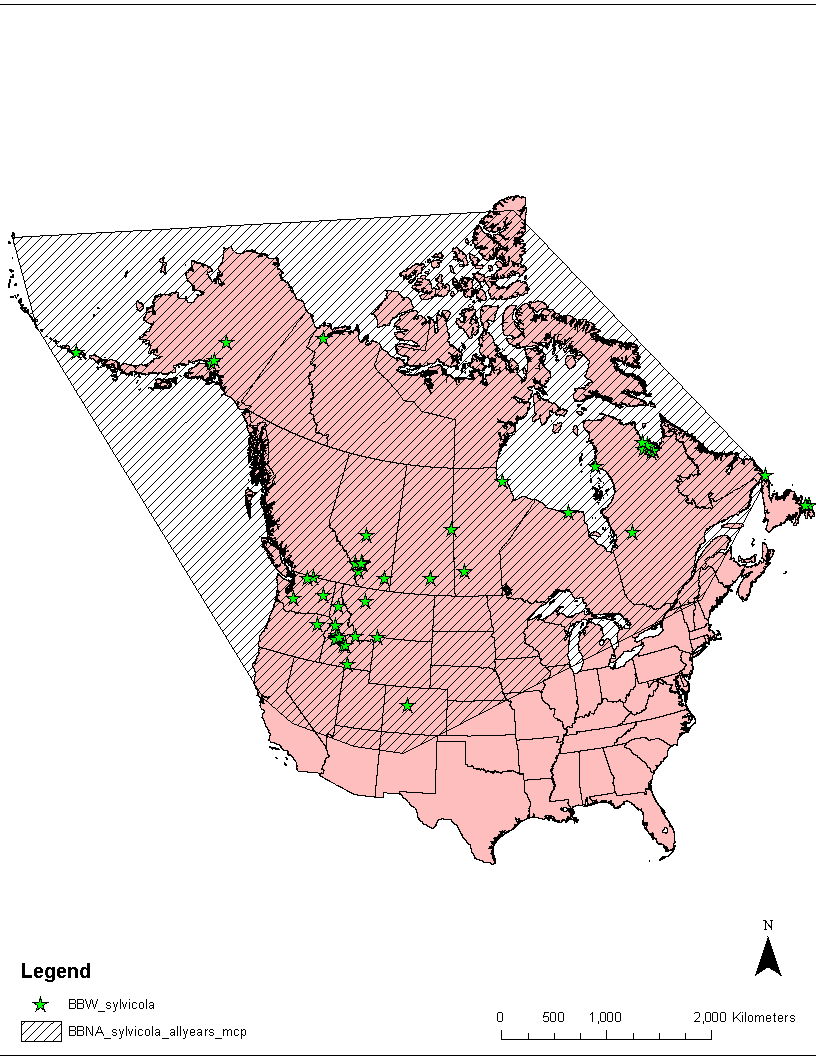

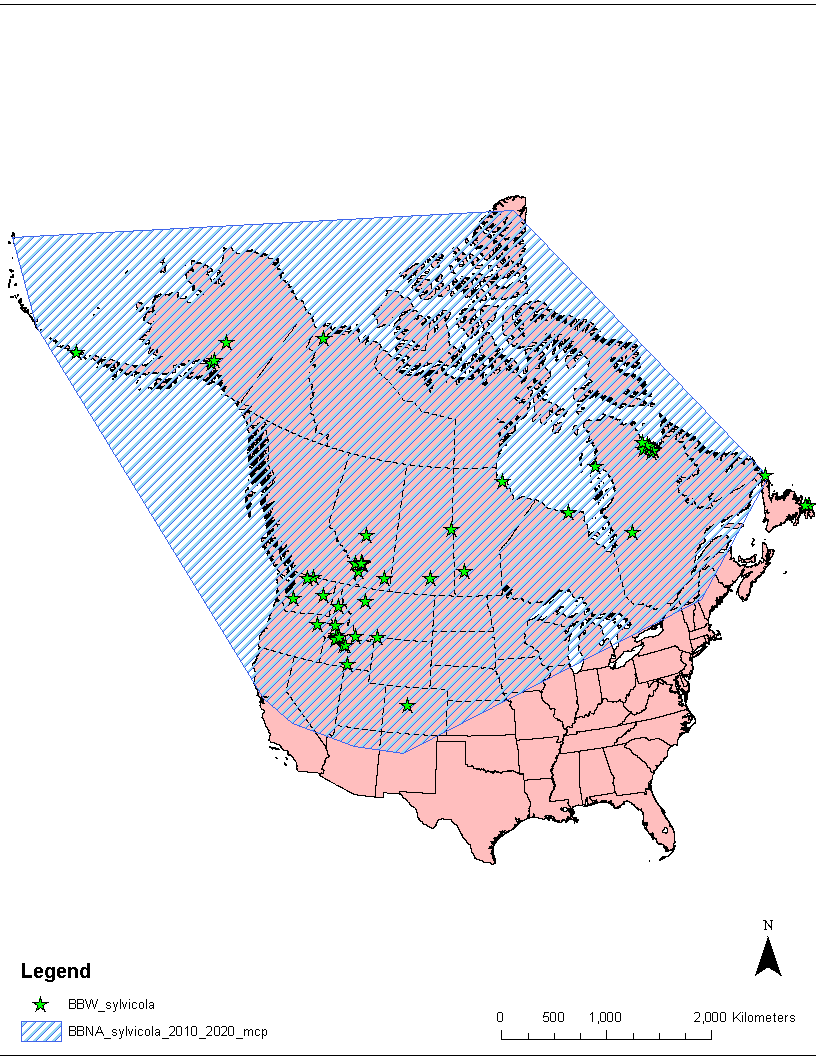
*

kk) *Bombus ternarius*

*
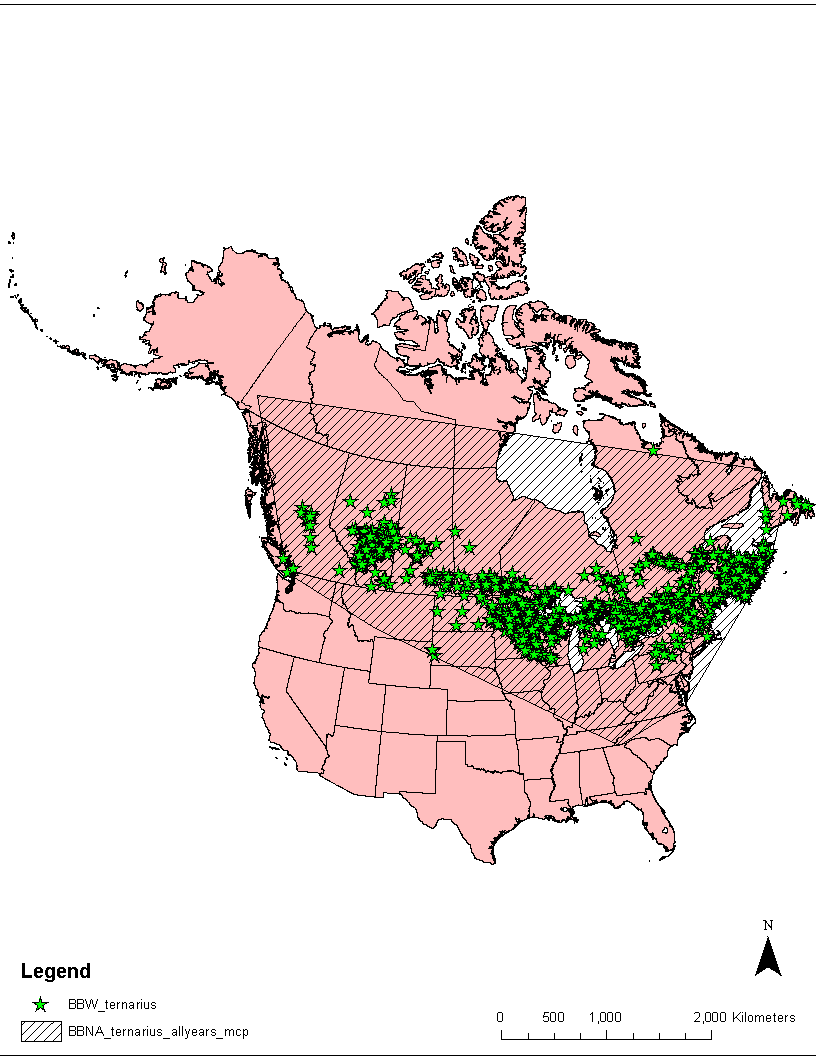

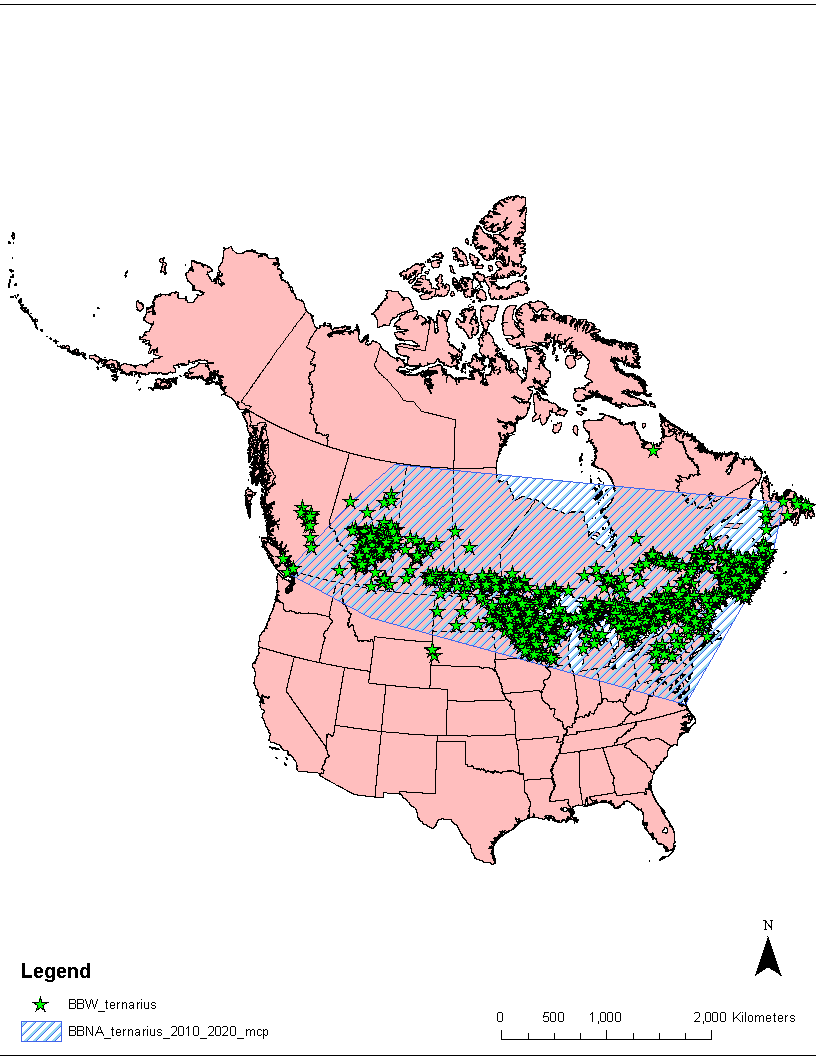
*

ll) *Bombus terricola*

*
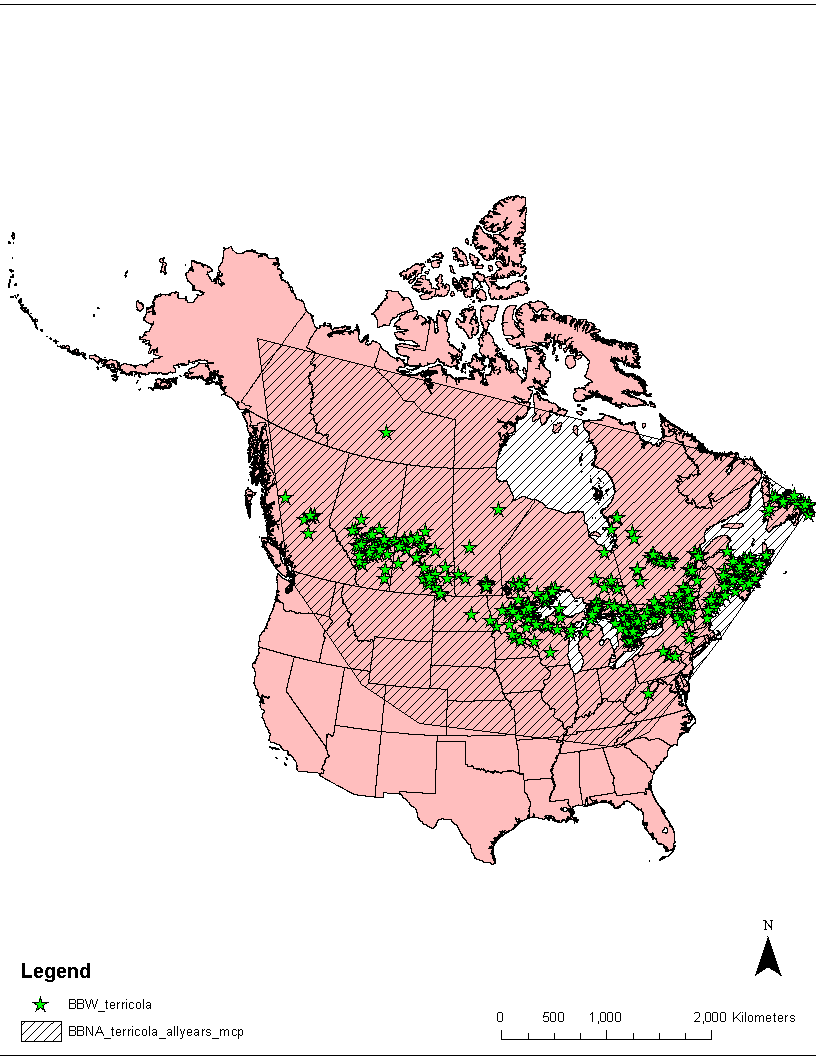

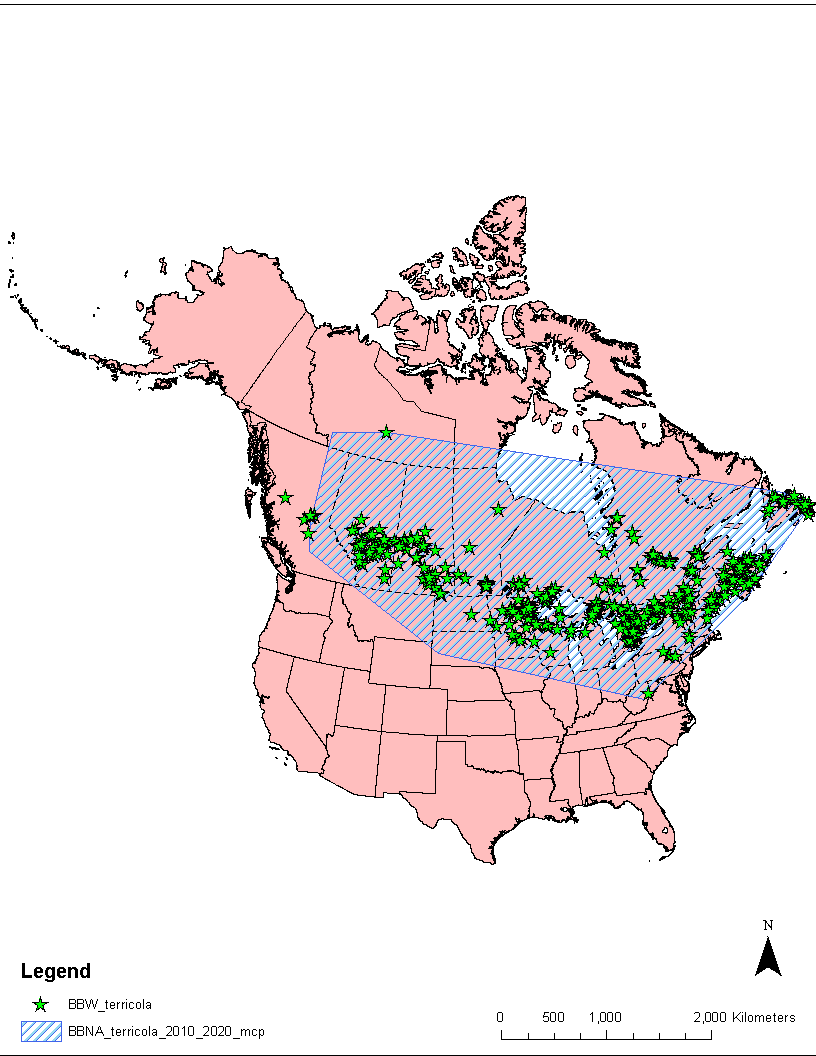
*

mm) *Bombus vagans*

*
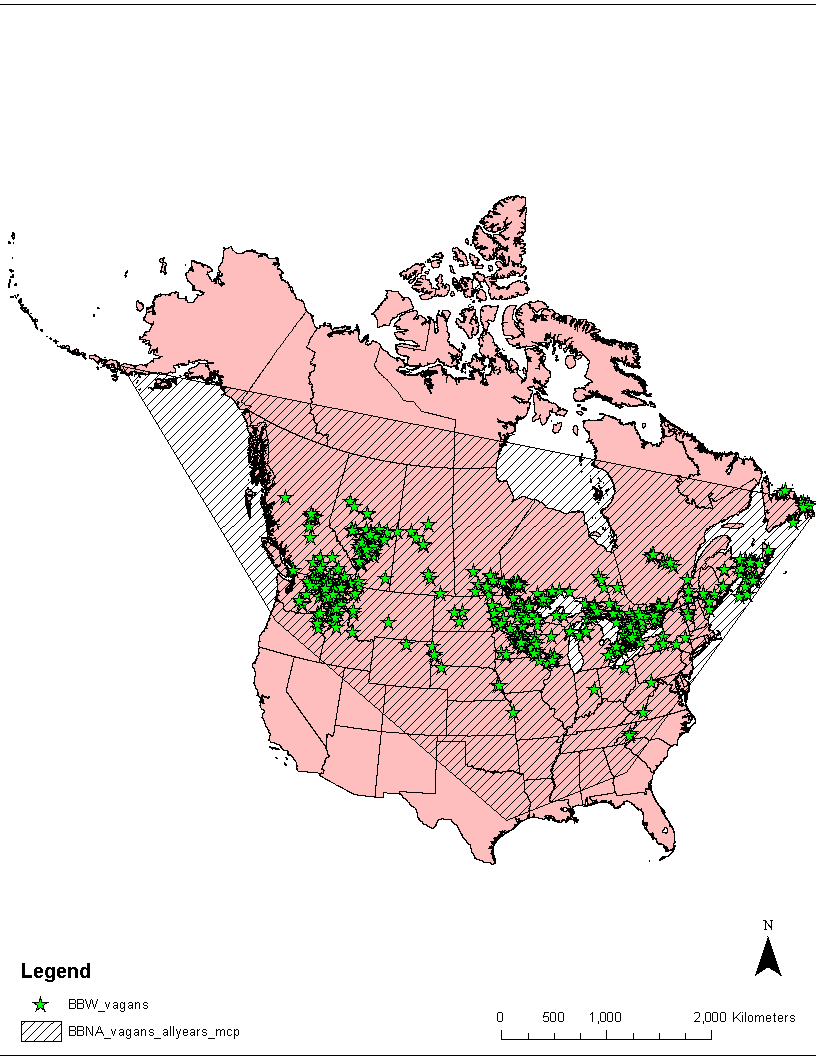

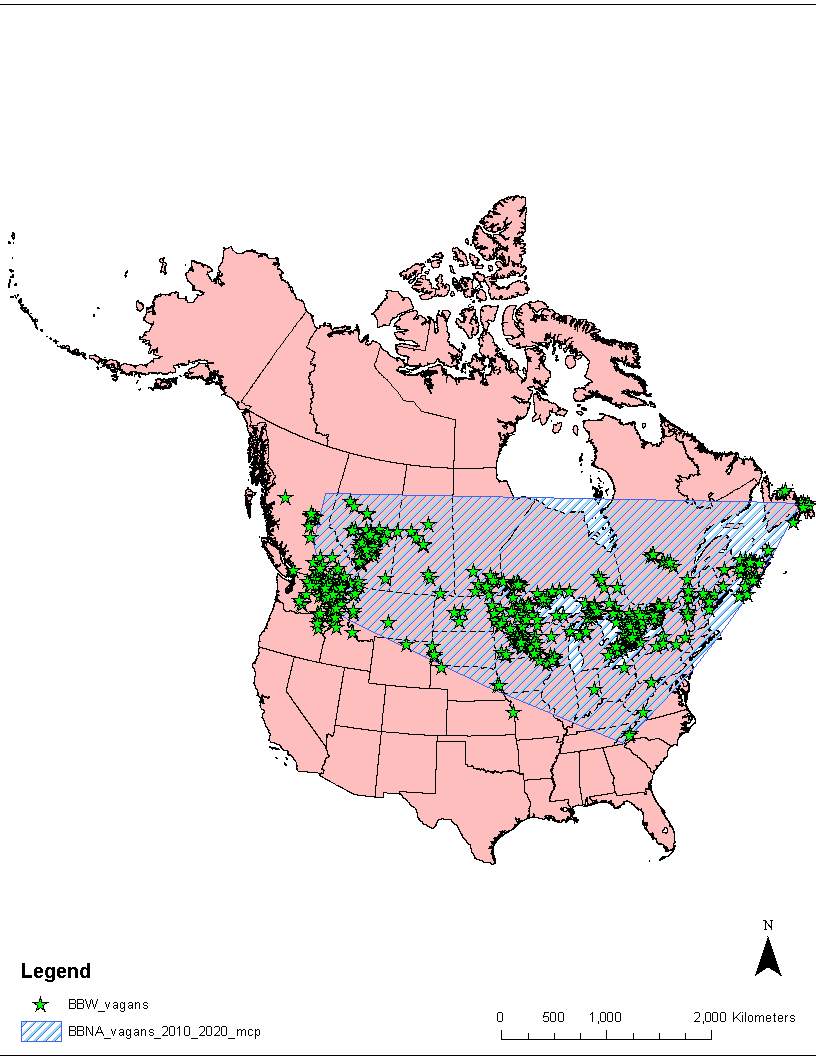
*

nn) *Bombus vandykei*

*
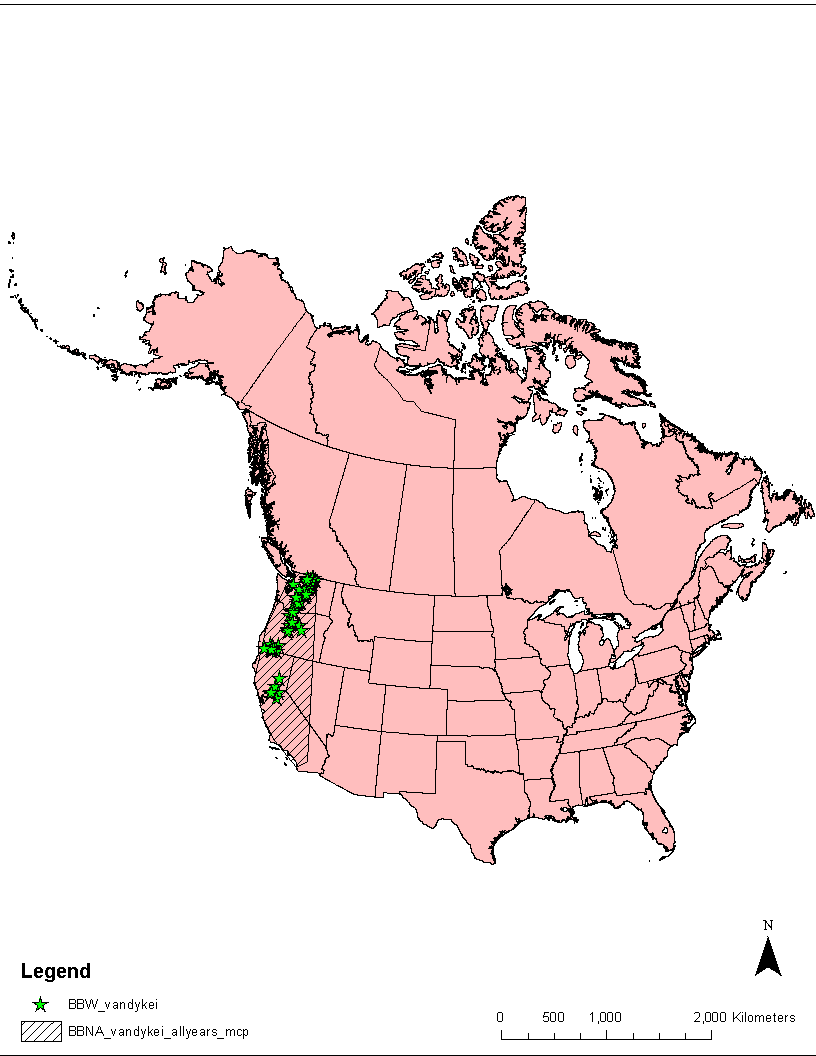

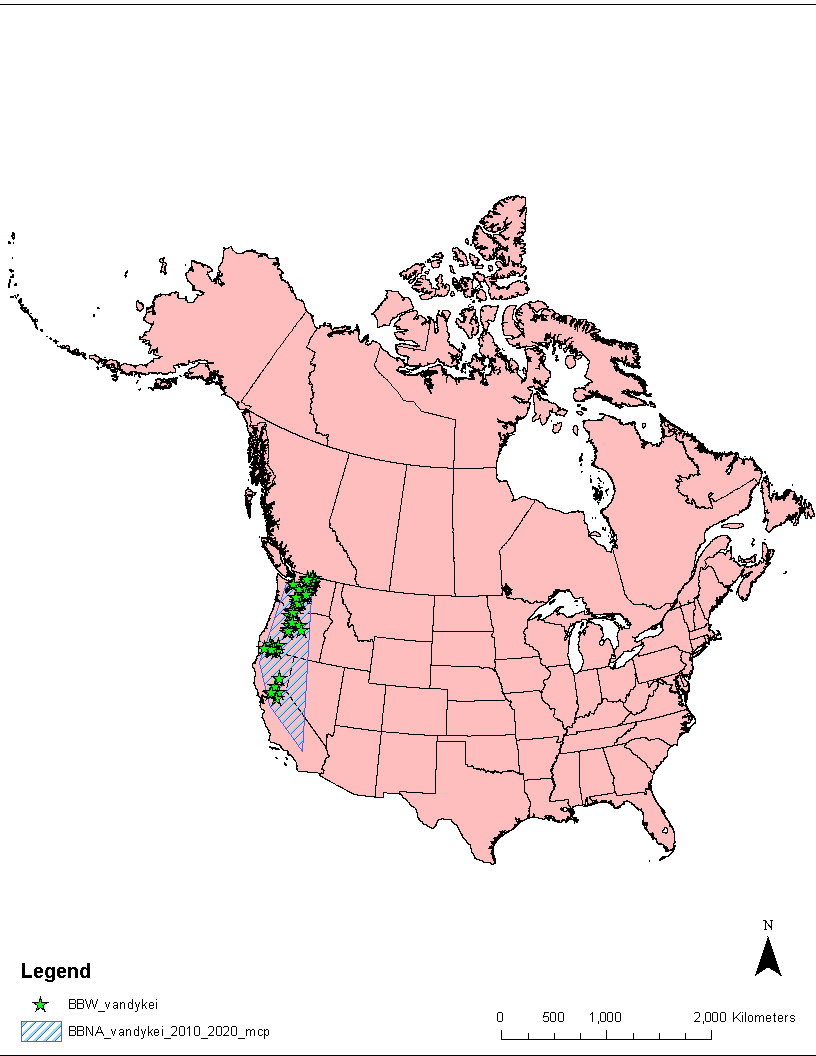
*

oo) *Bombus vosnesenskii*

*
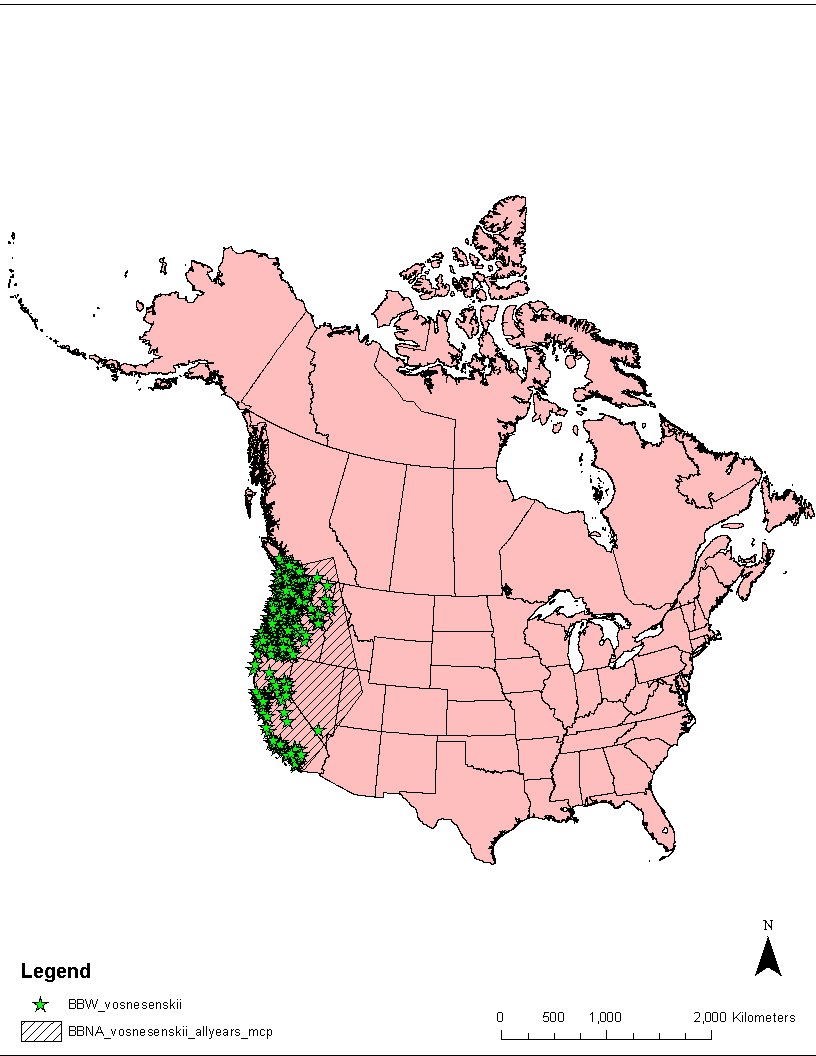

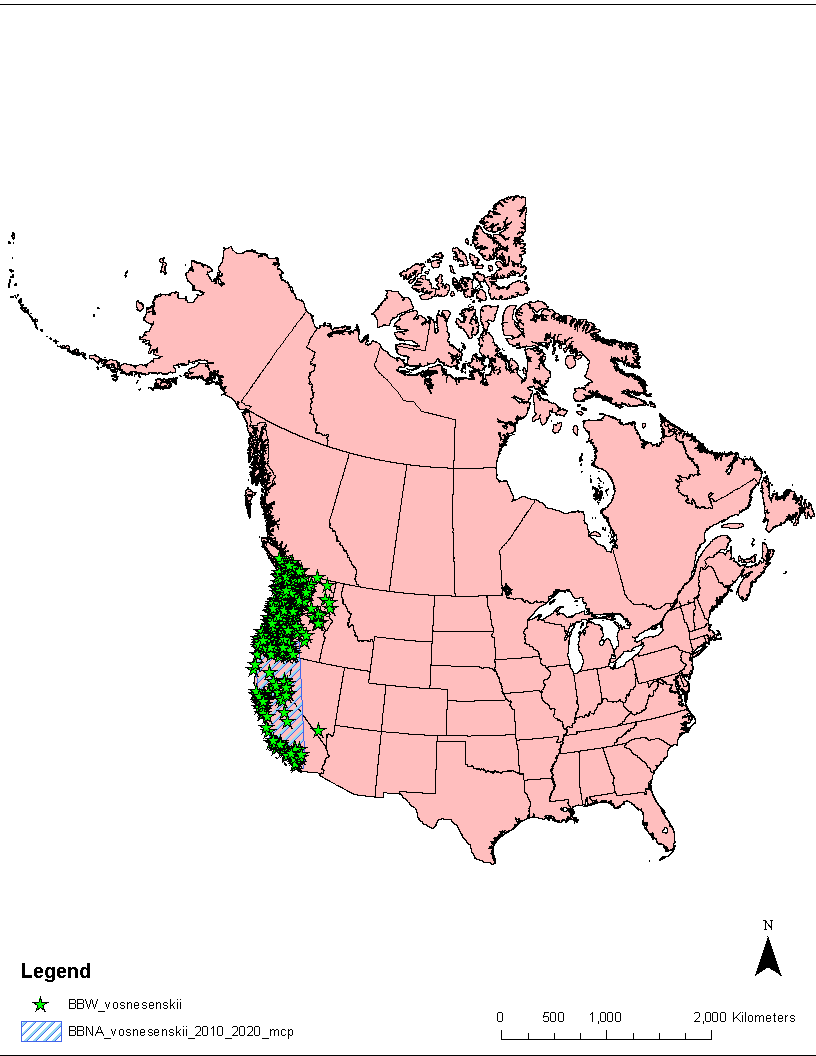
*

pp) *all Bombus species combined*

*
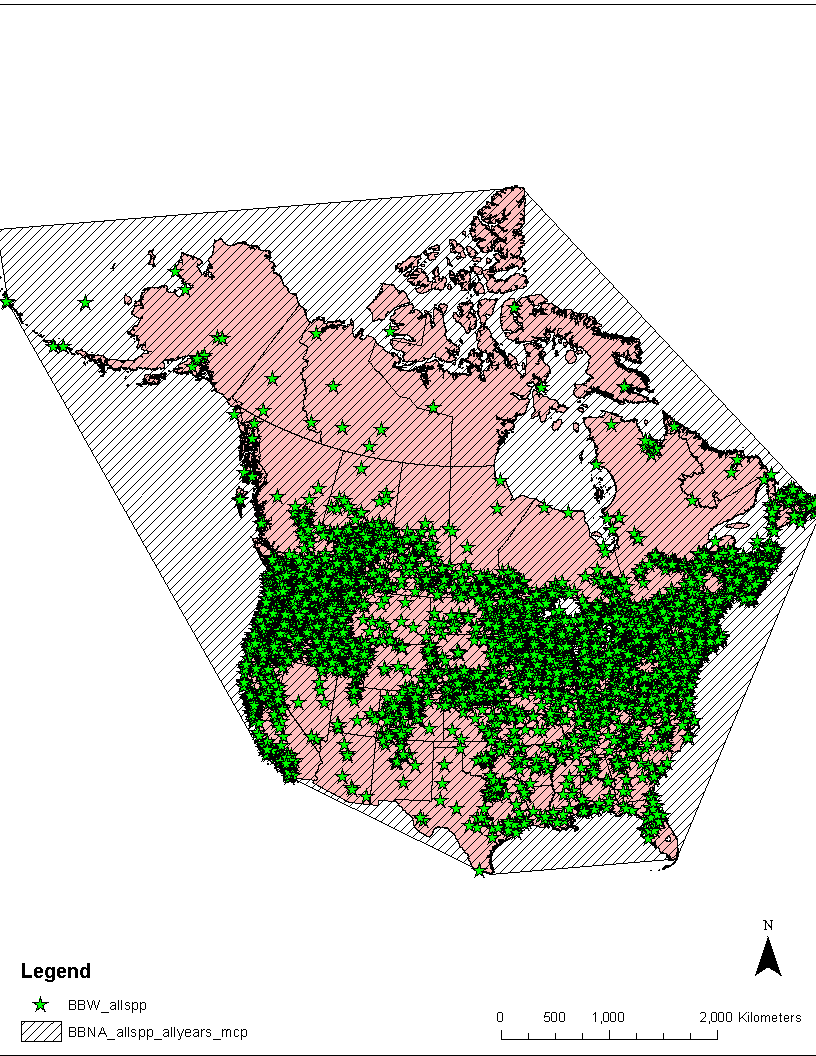

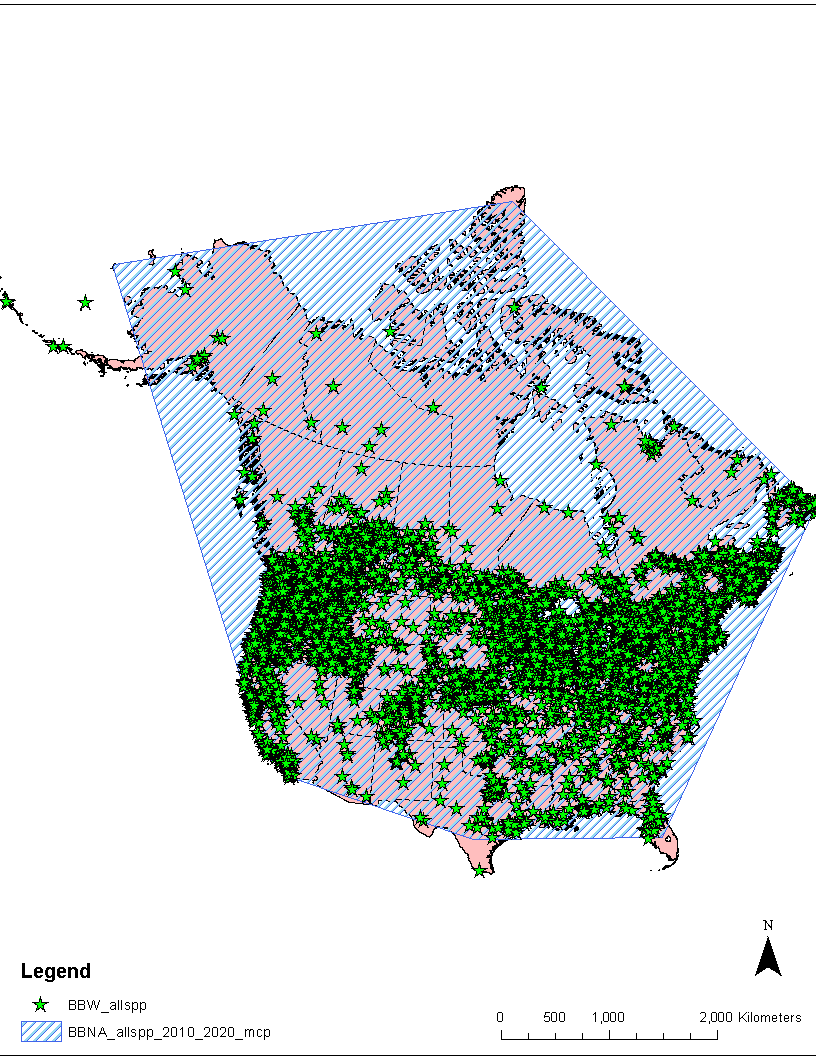
*

**S1 File Fig 1. Location of individual BBW records (green stars) as compared to the Extent of Occurrence area (EOO) for BBNA all years (black grid, left map) and BBNA 2010-2020 (blue grid, right map), for each of the 41 bumble bee (*Bombus*) species (FigsS1:a-pp) that were present in all three datasets**. Note that records located at the same or nearby locations may have overlapping stars, so the number of stars visible may not equal the number of records. The underlying maps contain information [1,2] licensed under the Open Government Licence – Canada [3] and the United States National Weather Service [4] , neither of which are subject to copyright protection.

**S1 File Fig 2** (following page). **A comparison of A) the total number of plant genera recorded per bumble bee species in BBNA all years (black bars), BBNA 2010-2020 (yellow bars), and BBW (green bars) datasets, and B) the percent unique plant genera recorded from BBW as compared to BBNA all years (blue bars) and BBNA 2010-2020 (orange bars).**

**S1 File Fig 3** (following pages). **A comparison of A) the total number of plant species recorded per bumble bee species in BBNA all years (black bars), BBNA 2010-2020 (yellow bars), and BBW (green bars) datasets, and B) the percent unique plant species recorded from BBW as compared to BBNA all years (blue bars) and BBNA 2010-2020 (orange bars).**

S1 File Fig 2.


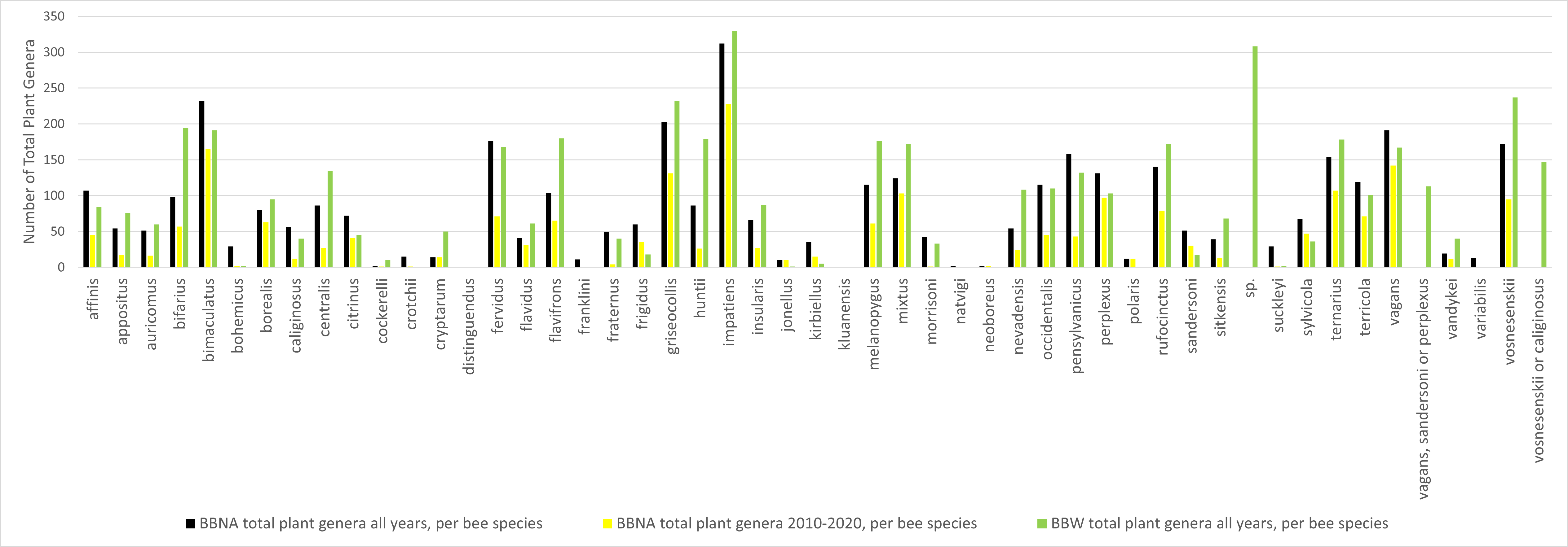


A


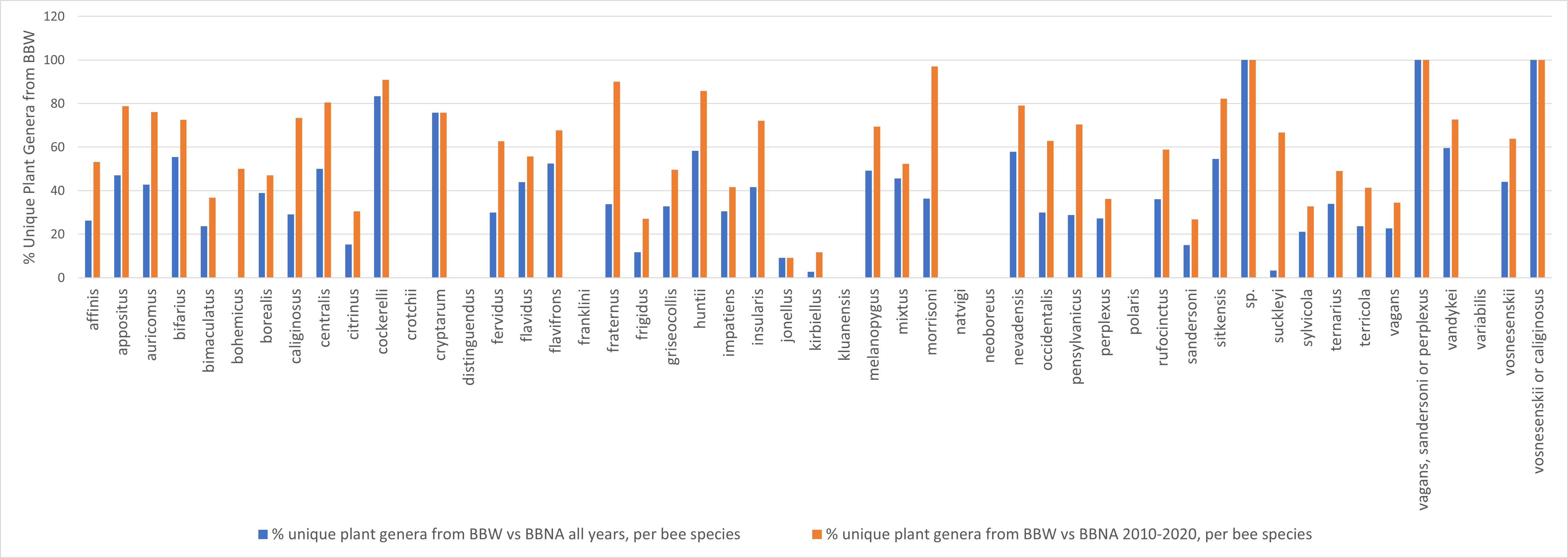


B

S1 File Fig 3.


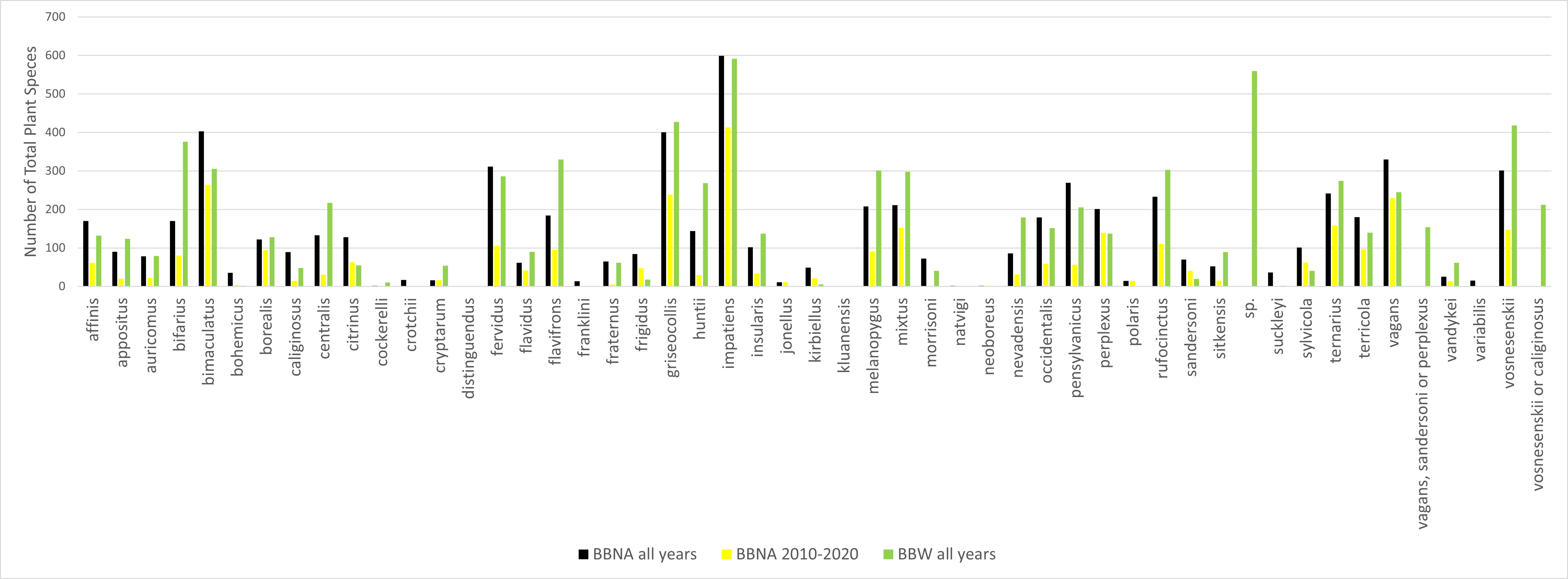


A


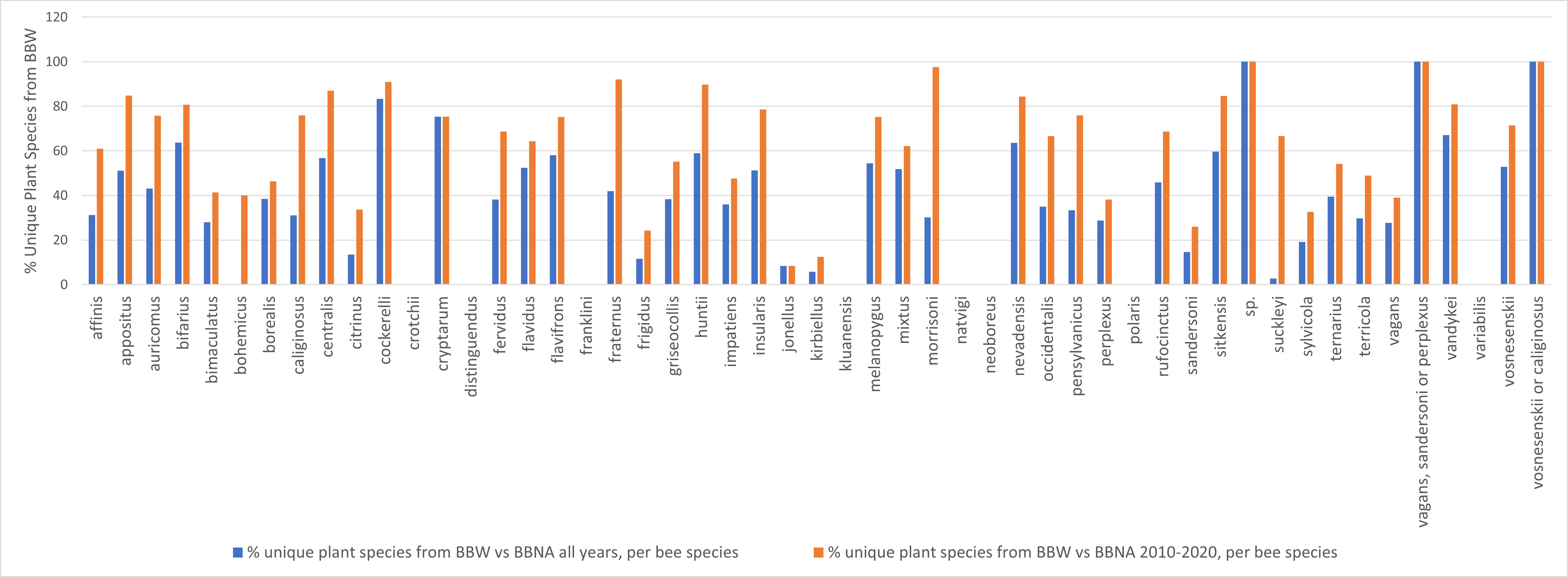


B

This concludes the Supplementary Information File 1 for this Paper

References

1. Statistics Canada. Provinces/territories, cartographic boundary file - 2016 census (2016-11-16). Government of Canada; 2016. Available: https://open.canada.ca/data/en/dataset/a883eb14-0c0e-45c4-b8c4-b54c4a819edb

2. US National Weather Service. U.S. States and Territories - 11 August 2016. US National Oceanic and Atmospheric Administration; 2016. Available: https://www.weather.gov/gis/USStates

3. Government of Canada. Open Government Licence - Canada, v2.0. 2022 [cited 7 Jan 2024]. Available: https://open.canada.ca/en/open-government-licence-canada

4. US National Weather Service. Disclaimer. [cited 4 Jan 2024]. Available: https://www.weather.gov/disclaimer

5. Koch JB, Rodriguez J, Pitts JP, Strange JP. Phylogeny and population genetic analyses reveals cryptic speciation in the Bombus fervidus species complex (Hymenoptera: Apidae). PLoS One. 2018;13: 1–20. doi:10.1371/journal.pone.0207080

6. Yanega D. The status of Cockerell’s bumblebee, Bombus (Pyrobombus) cockerelli Franklin, 1913 (Hymenoptera: Apidae). Southwest Entomol. 2013;38: 517–522.

7. Sheffield CS, Oram R, Heron JM. Bombus (Pyrobombus) johanseni sladen, 1919, a valid North American bumble bee species, with a new synonymy and comparisons to other “red-banded” bumble bee species in north america (Hymenoptera, Apidae, Bombini). Zookeys. 2020;2020: 59–81. doi:10.3897/zookeys.984.55816

8. Martinet B, Lecocq T, Brasero N, Gerard M, Urbanová K, Valterová I, et al. Integrative taxonomy of an arctic bumblebee species complex highlights a new cryptic species (Apidae: Bombus). Zool J Linn Soc. 2019;187: 599–621. doi:10.1093/zoolinnean/zlz041

9. Williams PH, Byvaltsev AM, Cederberg B, Berezin M V, Frode Ø, Rasmussen C, et al. Genes suggest ancestral colour polymorphisms are shared across morphologically cyptic species in arctic bumblebees. PLoS Med. 2015;10: e0144544. doi:10.1371/journal.pone.0144544

10. Williams PH. Not just cryptic, but a barcode bush: PTP re-analysis of global data for the bumblebee subgenus Bombus s. str. supports additional species (Apidae, genus Bombus). J Nat Hist. 2021;55: 271–282. doi:10.1080/00222933.2021.1900444

11. Williams PH, Cannings SG, Sheffield CS. Cryptic subarctic diversity: a new bumblebee species from the Yukon and Alaska (Hymenoptera: Apidae). J Nat Hist. 2016;50: 2881–2893. doi:10.1080/00222933.2016.1214294

12. Ghisbain G, Lozier JD, Rahman SR, Ezray BD, Tian L, Ulmer JM, et al. Substantial genetic divergence and lack of recent gene flow support cryptic speciation in a colour polymorphic bumble bee (Bombus bifarius) species complex. Syst Entomol. 2020;45: 635–652. doi:10.1111/syen.12419

13. NatureServe. NatureServe Explorer [web application]. In: NatureServe, Arlington, Virginia [Internet]. 2021 [cited 24 Jan 2021]. Available: https://explorer.natureserve.org/

14. Committee on the Status of Endangered Wildlife in Canada. COSEWIC assessment and status report on the Gypsy Cuckoo bumble bee Bombus bohemicus in Canada. Ottawa, Ontario; 2014. Available: www.registrelep-sararegistry.gc.ca/default_e.cfm

15. Committee on the Status of Species at Risk in Ontario, COSSARO. Ontario species at risk evaluation report for Gypsy Cuckoo bumble bee (Bombus bohemicus). Minist Nat Resour. 2014; 1–10. Available: https://www.ontario.ca/page/ontario-species-risk-evaluation-report-gypsy-cuckoo-bumble-bee-bombus-bohemicus

16. Williams P, Thorp R, Richardson L, Colla S. Bumble Bees of North America. Princeton, New Jersey: Princeton University Press; 2014.

17. IUCN. The IUCN Red List of threatened species. Version 2020-3. 2020 [cited 24 Jan 2021]. Available: https://www.iucnredlist.org
